# Use of the oral beta blocker bisoprolol to reduce the rate of exacerbation in people with chronic obstructive pulmonary disease (COPD): a randomised controlled trial (BICS)

**DOI:** 10.1186/s13063-022-06226-8

**Published:** 2022-04-14

**Authors:** Seonaidh Cotton, Graham Devereux, Hassan Abbas, Andrew Briggs, Karen Campbell, Rekha Chaudhuri, Gourab Choudhury, Dana Dawson, Anthony De Soyza, Shona Fielding, Simon Gompertz, John Haughney, Chim C. Lang, Amanda J. Lee, Graeme MacLennan, William MacNee, Kirsty McCormack, Nicola McMeekin, Nicholas L. Mills, Alyn Morice, John Norrie, Mark C. Petrie, David Price, Philip Short, Jorgen Vestbo, Paul Walker, Jadwiga Wedzicha, Andrew Wilson, Brian J. Lipworth

**Affiliations:** 1 Centre for Healthcare Randomised Trials (CHaRT), University of Aberdeen, Aberdeen, AB25 2ZD UK; 2 Liverpool School of Tropical Medicine, Liverpool, L3 5QA UK; 3 Division of Applied Medicine, University of Aberdeen, Aberdeen, AB25 2ZD UK; 4 Institute of Health & Wellbeing, University of Glasgow, 1 Lilybank Gardens, Glasgow, G12 8RZ UK; 5 Gartnavel General Hospital, University of Glasgow, Glasgow, G12 0YN UK; 6 Royal Infirmary of Edinburgh, Edinburgh, EH16 4SA UK; 7 University of Newcastle, Medical School, Newcastle Upon Tyne, NE2 4HH UK; 8 Medical Statistics Team, Institute of Applied Health Sciences, University of Aberdeen, Aberdeen, AB25 2ZD UK; 9 Queen Elizabeth Hospital Birmingham, Birmingham, B15 2WB UK; 10 Centre of Academic Primary Care, University of Aberdeen, Aberdeen, AB25 2ZD UK; 11 Ninewells Hospital and Medical School, University of Dundee, Dundee, DD1 9SY UK; 12 MRC Centre for Inflammation Research, University of Edinburgh, Edinburgh, EH16 4TJ UK; 13 BHF Centre for Cardiovascular Science, University of Edinburgh, Edinburgh, EH16 4SB UK; 14 Cardiovascular and Respiratory Studies, Castle Hill Hospital, Hull, HU16 5JQ UK; 15 NINE Edinburgh BioQuarter, University of Edinburgh, 9 Little France Road, Edinburgh, EH16 4UX UK; 16 Glasgow Cardiovascular Research Centre, University of Glasgow, Glasgow, G12 8TD UK; 17 Ninewells Hospital, Dundee, DD1 9SY UK; 18 Division of Infection, Immunity and Respiratory Medicine, University of Manchester, Manchester, M23 9LT UK; 19 Liverpool University Hospitals Foundation NHS Trust, University Hospital Aintree, Lower Lane, Liverpool, L9 7AL UK; 20 National Heart and Lung Institute, Imperial College, London, SW3 6LY UK; 21 Department of Medicine, University of East Anglia, Norwich, NR4 7TJ UK

**Keywords:** COPD, Exacerbation, Randomised controlled trial, Bisoprolol, Beta blocker

## Abstract

**Background:**

Chronic obstructive pulmonary disease (COPD) is associated with significant morbidity, mortality and healthcare costs. Beta blockers are well-established drugs widely used to treat cardiovascular conditions. Observational studies consistently report that beta blocker use in people with COPD is associated with a reduced risk of COPD exacerbations. The bisoprolol in COPD study (BICS) investigates whether adding bisoprolol to routine COPD treatment has clinical and cost-effective benefits. A sub-study will risk stratify participants for heart failure to investigate whether any beneficial effect of bisoprolol is restricted to those with unrecognised heart disease.

**Methods:**

BICS is a pragmatic randomised parallel group double-blind placebo-controlled trial conducted in UK primary and secondary care sites. The major inclusion criteria are an established predominant respiratory diagnosis of COPD (post-bronchodilator FEV_1_ < 80% predicted, FEV_1_/FVC < 0.7), a self-reported history of ≥ 2 exacerbations requiring treatment with antibiotics and/or oral corticosteroids in a 12-month period since March 2019, age ≥ 40 years and a smoking history ≥ 10 pack years. A computerised randomisation system will allocate 1574 participants with equal probability to intervention or control groups, stratified by centre and recruitment in primary/secondary care. The intervention is bisoprolol (1.25 mg tablets) or identical placebo. The dose of bisoprolol/placebo is titrated up to a maximum of 4 tablets a day (5 mg bisoprolol) over 4–7 weeks depending on tolerance to up-dosing of bisoprolol/placebo—these titration assessments are completed by telephone or video call. Participants complete the remainder of the 52-week treatment period on the final titrated dose (1, 2, 3, 4 tablets) and during that time are followed up at 26 and 52 weeks by telephone or video call. The primary outcome is the total number of participant reported COPD exacerbations requiring oral corticosteroids and/or antibiotics during the 52-week treatment period. A sub-study will risk stratify participants for heart failure by echocardiography and measurement of blood biomarkers.

**Discussion:**

The demonstration that bisoprolol reduces the incidence of exacerbations would be relevant not only to patients and clinicians but also to healthcare providers, in the UK and globally.

**Trial registration:**

Current controlled trials ISRCTN10497306. Registered on 16 August 2018

**Supplementary Information:**

The online version contains supplementary material available at 10.1186/s13063-022-06226-8.

## Administrative information

Note: the numbers in curly brackets in this protocol refer to SPIRIT checklist item numbers. The order of the items has been modified to group similar items (see http://www.equator-network.org/reporting-guidelines/spirit-2013-statement-defining-standard-protocol-items-for-clinical-trials/).

**Table:** 

Title {1}	Use of the oral beta blocker bisoprolol to reduce the rate of exacerbation in people with chronic obstructive pulmonary disease (COPD): a randomised controlled trial. (BICS)
Trial registration {2a and 2b}.	Trial registration: ISRCTN10497306 (registered 16 August 2018; first participant recruited 16 October 2018) Adheres to WHO trial registration data set.
Protocol version {3}	Version 7, 14 May 2021
Funding {4}	NIHR Health Technology Assessment (15/130/20) British Heart Foundation (PG/17/64/33205)
Author details {5a}	1. University of Aberdeen, Centre for Healthcare Randomised Trials (CHaRT), Aberdeen. AB25 2ZD. UK. 2. Liverpool School of Tropical Medicine, Liverpool. L3 5QA. UK 3. University of Aberdeen, Division of Applied Medicine, Aberdeen. AB25 2ZD. UK 4. University of Glasgow, Institute of Health & Wellbeing, 1 Lilybank Gardens, Glasgow. G12 8RZ. UK. 5.University of Glasgow, Gartnavel General Hospital, Glasgow. G12 0YN. UK. 6. Royal Infirmary of Edinburgh, Edinburgh. EH16 4SA. UK. 7. University of Newcastle, Medical School, Newcastle Upon Tyne. NE2 4HH. UK. 8. University of Aberdeen, Medical Statistics Team, Institute of Applied Health Sciences, Aberdeen. AB25 2ZD. UK. 9. Queen Elizabeth Hospital Birmingham, Birmingham. B15 2WB. UK. 10. University of Aberdeen, Centre of Academic Primary Care, Aberdeen. AB25 2ZD. UK. 11. University of Dundee, Ninewells Hospital and Medical School, Dundee. DD1 9SY. UK. 12. University of Edinburgh, MRC Centre for Inflammation Research, Edinburgh. EH16 4TJ. UK. 13. University of Edinburgh, BHF Centre for Cardiovascular Science, Edinburgh. EH16 4SB. UK. 14. Cardiovascular and Respiratory Studies, Castle Hill Hospital, Hull. HU16 5JQ. UK. 15. University of Edinburgh, NINE Edinburgh BioQuarter, 9 Little France Road, Edinburgh. EH16 4UX. UK. 16. University of Glasgow, Glasgow Cardiovascular Research Centre, Glasgow. G12 8TD. UK. 17. Ninewells Hospital, Dundee, DD1 9SY 18. University of Manchester, Division of Infection, Immunity and Respiratory Medicine, Manchester. M23 9LT. UK 19. University Hospital Aintree, Liverpool University Hospitals Foundation NHS Trust, Lower Lane, Liverpool. L9 7AL. UK. 20. Imperial College, National Heart and Lung Institute, London. SW3 6LY. UK. 21. Department of Medicine, University of East Anglia, Norwich. NR4 7TJ. UK.
Name and contact information for the trial sponsor {5b}	Co-sponsor 1. University of Aberdeen, Foresterhill House Annexe, Foresterhill, Aberdeen, AB25 2ZB. UK. researchgovernance@abdn.ac.uk Co-sponsor 2. NHS Grampian, Foresterhill House Annexe, Foresterhill, Aberdeen, AB25 2ZB. UK. researchgovernance@abdn.ac.uk
Role of sponsor {5c}	The sponsor played no part in study design; and will play no part in the collection, management, analysis, and interpretation of data; writing of the report; and the decision to submit the report for publication.

## Introduction

### Background and rationale {6a}

Chronic obstructive pulmonary disease (COPD) is a lung disease characterised by progressive airflow obstruction [1]. Globally, 210 million people have moderate to severe COPD and prevalence is increasing [2, 3]. COPD is the fifth leading cause of death globally and by 2030 it is expected to be the fourth, accounting for 8% of deaths [4]. In 2002, COPD was the eleventh leading cause of disability-adjusted life years (DALY)s; by 2030, it is expected to be the seventh [4]. In the UK, the prevalence of diagnosed COPD has increased from about 991,000 in 2004 to 1.2 million in 2012 [5]; it is the fifth leading cause of death, accounting for about 30,000 deaths annually. The progressive airflow limitation of COPD is associated with increasing disability, work absence, long-term morbidity, physical and psychological co-morbidities and premature mortality. People with COPD are more likely to have associated comorbidities, including ischaemic heart disease, hypertension, diabetes and depression, and unrecognised heart failure has been reported in up to 20% of COPD patients [6–12].

Acute deteriorations in symptoms known as exacerbations are an important clinical feature of COPD. They are associated with accelerated lung function decline, reduced physical activity, reduced quality of life and increased mortality [13–16]. COPD is one of the costliest inpatient conditions and exacerbations account for about 60% of the £1 billion NHS expenditure on COPD [1, 17]. Despite advances in management there is still an unmet need for improved pharmacological treatment of COPD particularly the prevention of exacerbations.

Beta blockers are a class of drug with proven benefit in people with heart failure or ischemic heart disease, particularly those with left ventricular impairment. The rationale for repurposing beta blockers for use in COPD comes from the findings of observational cohort studies that beta blocker use in people with COPD is associated with a reduced risk of exacerbation [18–24]. A systematic review of 15 studies of beta blocker use for cardiovascular disease demonstrated that beta blocker use in COPD patients was associated with a 28% (95% CI 17–37) reduction in mortality and a 37% (95% CI 29–43) reduction in exacerbations [18]. The mechanisms by which beta blockers may reduce exacerbations remains uncertain although it is biologically plausible that some of the episodes conventionally diagnosed as acute exacerbations of COPD are in reality cardiac events for which beta blockers have proven benefits [25, 26].

Beta blockers competitively antagonise the effects of catecholamines on beta-adrenoreceptors. Beta1-adrenoreceptors are found only in the heart, whereas beta2-adrenoreceptors are more ubiquitous, being found in the heart and lungs. Bronchodilating beta2-agonists are the mainstay of COPD and asthma treatment and beta blockers that antagonise beta2-agonists could have adverse respiratory effects, and for asthma, a condition with reversible airflow limitation, beta blockers are usually avoided [27]. A systematic review of randomised controlled trials (RCTs) studying the effects of beta1-selective blockers in people with COPD reported that repeat administration of beta1-selective blockers was not associated with an increase in respiratory symptoms, change in lung function or a reduction in response to inhaled beta2-agonist [28]. Clinical evidence is supportive of beta1-blockers such as bisoprolol being safe in COPD and beta1-selective blocker use in COPD patients with heart failure is a guideline recommendation [29, 30].

We describe here the bisoprolol in COPD study (BICS) which tests the hypothesis that adding bisoprolol to routine COPD treatment reduces the rate of exacerbation. A sub-study will test the hypothesis that any beneficial effect of bisoprolol is restricted to those with unrecognised heart disease. The full protocol is available as a supplemental file.

### Objectives {7}

The primary objective is to determine the clinical and cost-effectiveness of adding bisoprolol to usual COPD treatment in patients with COPD at high risk of exacerbation as evidenced by a history of at least two COPD exacerbations in a previous year.

The secondary objectives are to compare the following outcomes between participants treated with bisoprolol and those treated with placebo:

- Hospital admissions with a primary diagnosis of COPD exacerbation
- Time to first exacerbation of COPD
- Total number of emergency hospital admissions
- Total number of major adverse cardiovascular events (MACE)
- Lung function
- Changes in breathlessness during treatment
- All-cause, respiratory and cardiac mortality
- Drug reactions and serious adverse events
- Health-related quality of life
- Disease specific health status
- Health care utilisation
- Incremental cost-per-exacerbation avoided
- Costs to the NHS and patients and lifetime cost-effectiveness based on extrapolation modelling
- Modelled lifetime incremental cost per quality-adjusted life year

The sub-study will risk stratify participants to investigate

o. Treatment effects in participants with and without unrecognised heart disease

### Trial design {8}

**Fig. 1:**
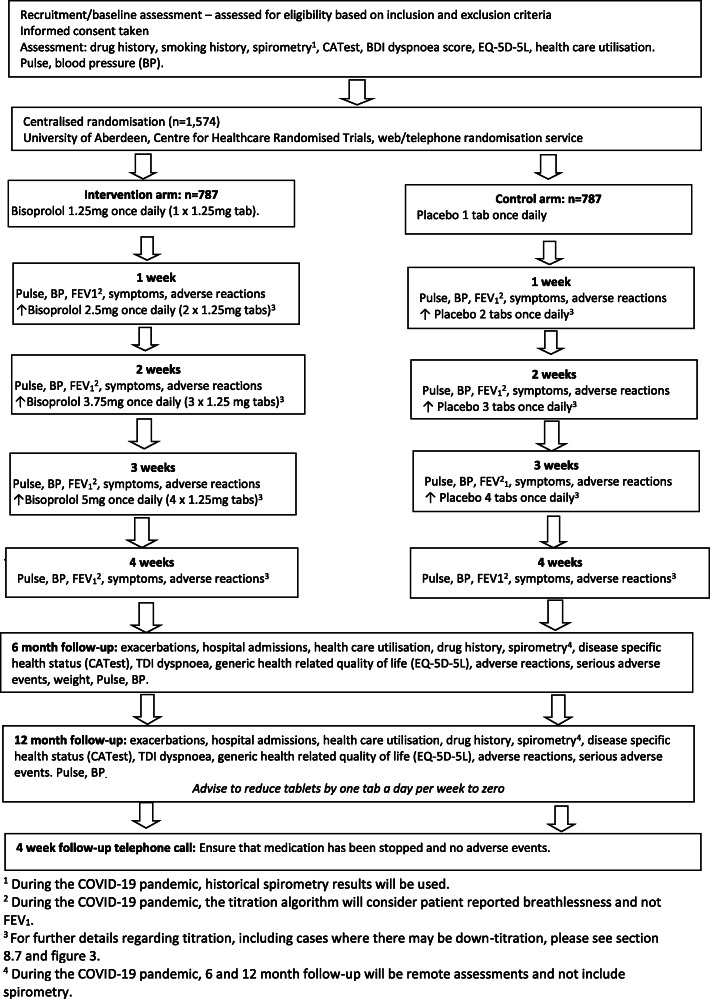
Flow diagram of study design and schedule. BDI, baseline dyspnoea index; BP blood pressure; CATest, COPD (Chronic Obstructive Pulmonary Disease) Assessment Test; EQ-5D-5L, EuroQOL five-dimension questionnaire; FEV_1_, forced expiratory volume 1 second; TDI transition dyspnoea index

BICS is a pragmatic randomised, double-blind, parallel group, placebo-controlled, multicentre clinical trial investigating whether bisoprolol is superior to placebo when added to current COPD therapy for 52 weeks in patients with COPD who have had two or more exacerbations of COPD in a previous year treated with oral corticosteroids (OCS) and/or antibiotics. Figure 1 provides a schematic representation of study design and schedule.

There is a sub-study to the main BICS study—that stratifies BICS participants into those with and without unrecognised heart disease based on blood biomarkers and echocardiogram to investigate whether any beneficial effect of bisoprolol is restricted to those with previously unrecognised heart disease.

## Methods: participants, interventions and outcomes

### Study setting {9}

Participants are being recruited from primary and secondary care sites across the UK. In primary care some General Practices are acting as recruitment sites, whereas others act as participant identification centres (PICs) with identified participants being evaluated in other primary or secondary care recruitment sites. A list of the study sites can be found at https://w3.abdn.ac.uk/hsru/BICS/Public/Public/index.cshtml.

### Eligibility criteria {10}

Patients will be enrolled if they meet all of the following criteria:

- Aged ≥ 40 years
- A smoking history of at least 10 pack years ([average number of cigarettes/day x years smoked]/20)
- An established predominant diagnosis of COPD (NICE Guideline definition: post bronchodilator FEV_1_ < 80% predicted, FEV_1_/FVC < 0.7) [1] receiving treatment as per local guidelines’. Patients with asthma-COPD overlap syndrome (ACOS) will also be eligible
- A history of at least two exacerbations requiring treatment with antibiotics and/or oral corticosteroid use in the previous year, based on patient report OR a history of at least two exacerbations within 12 months of each other requiring treatment with antibiotics and/or oral corticosteroid since March 2019
- Clinically stable with no COPD exacerbation for at least 4 weeks
- Able to swallow study medication
- Able and willing to give informed consent to participate
- Able and willing to participate in the study procedures, complete study questionnaire
- Able and willing to undergo spirometric assessment, able to perform an FEV_1_ manoeuvre as a minimum. During the COVID-19 pandemic, measurement of FEV_1_ is not required as part of the protocol, and therefore, this inclusion criterion does not need to be met.

The main exclusion criteria are a diagnosis of asthma before the age of 40 years, a predominant respiratory disease other than COPD, use of beta blockers, known intolerance to beta blockers, use of drugs contraindicated with beta blockers [31], resting heart rate < 60 beats per min (bpm) , systolic blood pressure < 100 mmHg, severe arterial occlusive disease, severe forms of Raynaud’s syndrome, a history of psoriasis and conditions for which beta blocker use is a guideline recommendation, e.g. heart failure. A detailed list all the exclusion criteria is included in the supplementary file. Concomitant use of drugs advised to be used with caution with beta blockers is permitted. For women, current pregnancy or breast-feeding, or planned pregnancy during the study are exclusion criteria.

### Who will take informed consent? {26a}

Consent is received by a suitably trained member of the research team at the recruitment site.

### Additional consent provisions for collection and use of participant data and biological specimens {26b}

Participants who wish to take part in the cardiac sub-study are asked to provide separate consent for this.

### Interventions

#### Explanation for choice of comparator {6b}

The use of placebo as comparator is acceptable because of clinical equipoise around the use of bisoprolol in people with COPD.

#### Intervention description {11a}

**Fig. 2:**
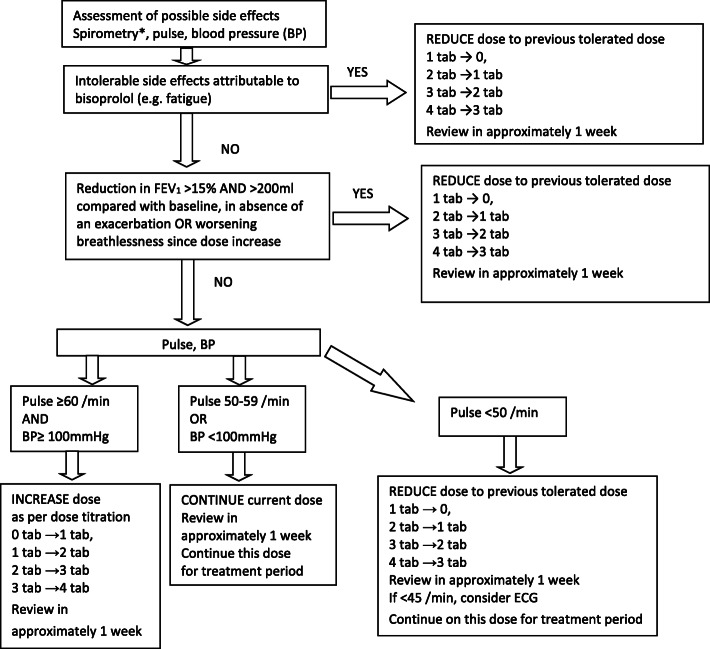
Schematic representation of dose titration decision-making

Participants will take either the cardio-selective beta blocker bisoprolol (1.25 mg tablets) or identical placebo for 52 weeks. Both are manufactured by Tiofarma B.V (Oud-Beijerland, Netherlands) and supplied by Mawdsley Brooks & Co (Doncaster, UK). Supplies of study drugs are couriered to the participants’ homes. To ensure participant safety the starting dose for bisoprolol is one 1.25 mg tablet taken orally daily and participants undergo a weekly dose titration regime (i.e. weekly increments of 1.25 mg→2.5 mg→3.75 mg→5 mg) that results in final doses of 1.25 mg once daily (od) (1 tab), 2.50 mg od (2 tabs), 3.75 mg od (3 tabs) or 5 mg od (4 tabs) depending on tolerance to bisoprolol up dosing. Participants allocated to placebo will undergo an identical dose-titration regime, with a final dose of 1, 2, 3 or 4 tablets a day. Participants complete the remainder of the 52-week treatment period on the final titrated dose. Figure 2 outlines the dose titration algorithm, decisions to increase, reduce or to fix on a dose during the titration period are determined by participant reports of intolerable side effects, heart rate, systolic blood pressure and self-reported changes to breathing. A computerised advisory titration algorithm is available and detailed in the supplementary file. Site staff can follow the titration advice or make an alternative decision about titration; this is documented as part of the case report form.

Following completion of the 52-week treatment period, participants will be weaned off study medication over the following 3 weeks (3-2-1 tablet od), cessation of study medication is confirmed by a telephone contact.

#### Criteria for discontinuing or modifying allocated interventions {11b}

During the 52-week treatment period the dose of study medication may be reduced at participant request or the development of possible adverse reactions. Participants may be withdrawn from treatment if consent for treatment is withdrawn, they develop unacceptable adverse reactions, they develop a condition for which beta blockers are clinically indicated, e.g. acute coronary syndrome, or a clinician wishes to prescribe a medication contraindicated with bisoprolol. Participants who withdraw consent are advised to wean down the study medication, participants withdrawn from treatment for other clinical reasons (i.e. adverse reactions) are advised to stop study treatment with immediate effect. Participants discontinuing study medication are invited to remain in the study and followed up in accordance with the trial protocol.

#### Strategies to improve adherence to interventions {11c}

Adherence with study treatment is assessed at each titration assessment and at the 26 and 52-week follow-up assessments by asking participants to estimate their adherence [32]. Participant’s preferences are incorporated into the titration process, so for example, if they do not wish to increase dose at any time, their preference is accommodated.

#### Relevant concomitant care permitted or prohibited during the trial {11d}

Participants remain on their usual COPD medications throughout the study period and clinicians are advised to manage participants in the usual manner subject to the caveats outlined above.

#### Provisions for post-trial care {30}

At the end of the study, participants and their GPs will be informed of the study results and their allocation status. If at the end of their involvement a participant wishes to take bisoprolol, the participant’s GP will be advised of this by letter. This letter will indicate that this would be off-label use of bisoprolol that the patient may have been on placebo or bisoprolol and that dose-titration would be required.

#### Outcomes {12}

The primary outcome measure is the total number of exacerbations of COPD necessitating changes in management (minimum management change—use of oral corticosteroids or antibiotics) during the 52-week treatment period, as reported by the participant. This clinically important outcome will be aggregated as mean events per year. The primary economic outcome measure is cost-per-quality-adjusted life year (QALY) gained during the 52-week treatment period; this will be aggregated as a mean value.

The secondary outcomes will be quantified for the 52-week treatment period and are: number of participant reported COPD exacerbations requiring hospital admission; number of participant reported emergency hospital admissions (all causes); number of major adverse cardiovascular events (MACE) [33]; all-cause, respiratory and cardiac mortality; and utilisation of primary or secondary health care services for respiratory events. These outcomes will be expressed as a rate—number of events during the year of treatment. Additional secondary outcomes during the 52-week treatment period are as follows: time to first exacerbation of COPD; post bronchodilator lung function (FEV_1_, FVC) (although because of COVID-19 this outcome will not be available for many participants); breathlessness using Baseline and Transition Dyspnoea Indices (BDI & TDI) [34]; serious adverse events, adverse reactions; health-related quality of life using EuroQoL 5D (EQ-5D-5L) Index [35]; disease specific health status using the COPD Assessment Test (CAT) [36]; and modelled lifetime incremental cost per QALY.

#### Participant timeline {13}

Potential participants usually receive a postal invitation to participate that includes a participant information sheet and details on how to contact the local study team.

**Fig. 3:**
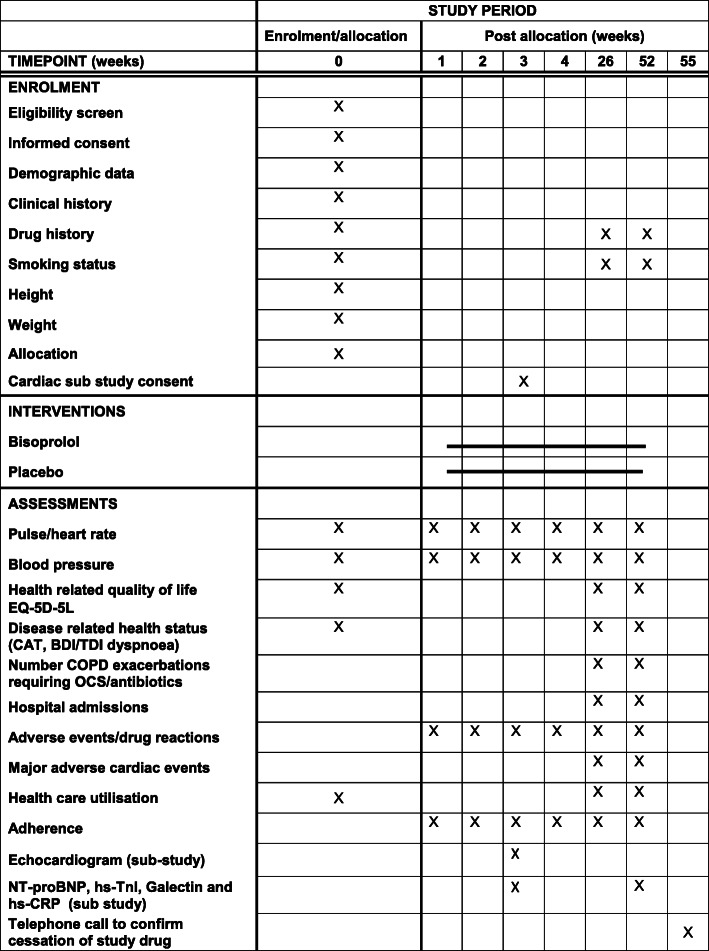
Schedule of enrolment, interventions, and assessments. BDI, baseline dyspnoea index; CATest, COPD (Chronic Obstructive Pulmonary Disease) Assessment Test; EQ-5D-5L, EuroQOL five-dimension questionnaire; hs-cTnI high-sensitivity cardiac troponin I; NTproBNP N-terminal pro-Brain Natriuretic PeptideN; OCS oral corticosteroids; TDI transition dyspnoea index

The participant timeline is outlined in Fig. 3. Participants are recruited and consented at a baseline face to face or telephone/video assessment. Participants are then reviewed at weekly telephone/video assessments for dose titration. The titration assessments are a minimum of one week apart, the usual time available (determined by first supply of study medication) for dose titration is 7 weeks, this reflects ‘real life’ by accommodating participant’s needs and wishes; however, this period can be extended if required (for example if the participant has an exacerbation of their COPD during the titration period). Further follow-up telephone/video assessments take place at 26 and 52 weeks into the treatment period. After the 52-week treatment period, participants are contacted by telephone after an appropriate number of weeks (1 week per tablet) to confirm that they have weaned off the study medication.

Participants recruited in secondary care sites contributing to the cardiac sub-study are invited to participate in the sub-study during the titration phase, a separate consent is obtained covering echocardiography and collection and analysis of blood samples.

#### Sample size {14}

The multicentre Evaluation of COPD Longitudinally to Identify Predictive Surrogate Endpoints (ECLIPSE) study reported the frequency of COPD exacerbation in 2138 patients [37]. For patients identical to our target population (≥ 2 self-reported COPD exacerbations in a year requiring antibiotics and/or OCSs), the mean (SD) number of COPD exacerbations within the subsequent 1 year was 2.22 (1.86). The Cardiac Insufficiency Bisoprolol Study II (CIBIS -II) reported that about 15% of participants stopped taking study medication in a trial of bisoprolol vs placebo [26].

Assuming a similar rate in the placebo arm, 669 participants are needed in each arm of the trial to detect a clinically important reduction in COPD exacerbations of 15% (i.e. from an average of 2.22 to 1.89) with 90% power at the two-sided 5% significance level. Allowing for an estimated 15% withdrawal from study treatment 787 participants are required in each study group (i.e. 1,574 in total).

#### Recruitment {15}

Potential participants will be recruited from both primary and secondary care sites across the UK, the aim being to recruit the majority of participants (> 50%) in primary care sites. Recruitment strategies will differ between centres depending on local geographic and NHS organisational factors.

##### Primary care

Recruitment in General Practices will be conducted in conjunction with the NIHR Clinical Research Network (CRN) in England and the NHS Research Scotland Primary Care Network in Scotland. In primary care, potential participants are identified from General Practice databases that are searched for the major inclusion and exclusion criteria. Preliminary lists of potential participants are reviewed by a medical practitioner. In some centres, community-based respiratory services, e.g. pulmonary rehabilitation, respiratory nurses are available and are used to identify potential participants. Recruitment in primary care is supplemented by posters located in General Practice waiting areas and Community Pharmacies.

##### Secondary care

In secondary care, potential participants are identified from patients attending hospital respiratory out-patient clinics, spirometry services, smoking cessation services and early supported discharge services. Some trial centres also have access to Volunteer Databases/Registries. Potential participants in the cardiac sub-study are participants who have been recruited in secondary care sites contributing to the sub-study.

### Assignment of interventions: allocation

#### Sequence generation {16a}

Participants will be enrolled and consented by a member of the site research team who then use a computerised web based randomisation service to allocate the participant to a treatment group. The randomisation service is administered by the Centre for Healthcare Randomised Trials (CHaRT), University of Aberdeen. The randomisation is stratified by trial centre (or area for primary care sites), and recruitment setting (primary or secondary care) and participants are randomised with equal probability (1:1) to the intervention and control groups. The random allocation sequence has been generated using permuted blocks to provide randomly generated blocks of entries of varying sizes permuted for each combination of region and recruitment setting.

#### Concealment mechanism {16b}

The web-based randomisation system ensures allocation concealment.

#### Implementation {16c}

The randomisation system is embedded in the trial website. A member of the research team at the recruitment site randomises a participant after consent.

### Assignment of interventions: blinding

#### Who will be blinded {17a}

To ensure double blinding, bisoprolol and placebo tablets are identical in appearance, taste, touch and smell and dispensed in identically labelled containers. All trial participants, care providers, outcome assessors, trials managers and data analysts remain blinded to allocation status until database lockdown. Unblinding of allocation status is permitted to enable treatment of severe adverse event/s, overdose or to enable SUSAR reporting. The trial database includes an unblinding function with access limited to those permitted to unblind in and out of office hours. At the end of the 12 month follow-up period, if a participant requests information as to their allocation within the trial, for example to plan future treatment, the sponsor can agree to unblinding. In such cases, the CI and trial office team remain blind to the treatment allocation. Where possible the site team also remain blind to the treatment allocation.

#### Procedure for unblinding if needed {17b}

All sites have access to an automated telephone unblinding system that can be used to unblind a participant if necessary. In addition, participants are given an emergency card that includes an emergency contact number. The CI and their team have access to a web-based automated unblinding system in addition to the automated telephone unblinding system).

### Data collection and management

#### Plans for assessment and collection of outcomes {18a}

The schedule for data collection is outlined in Fig. 3. The following data are collected:

##### Drug history

Regular use of prescription drugs is recorded at recruitment and the 26- and 52-week assessments.

##### Smoking history

Smoking history (age commenced, age ceased, average cigarettes smoked per day) is recorded at recruitment and at the 26 and 52 week assessments. Pack year consumption is computed at recruitment.

##### Height and weight

Height and weight are measured at recruitment.

##### Heart rate and blood pressure

Resting heart rate and blood pressure are measured at recruitment and the dose titration assessments at weeks 1, 2, 3 and 4 and the 26- and 52-week assessments.

##### Number of COPD exacerbations

The primary outcome measure of the total number COPD exacerbations requiring antibiotics/oral corticosteroids whilst on study medication will be ascertained by asking the participants at the 26- and 52-week assessments. Participants are encouraged to record any exacerbations on a provided ‘reminder card’ and to have this available during their follow-up assessments. The American Thoracic Society/European Respiratory Society guideline definition of COPD exacerbation will be used: a worsening of patient’s dyspnoea, cough and/or sputum beyond day-to-day variability sufficient to warrant a change in management [38]. The minimum management change will be treatment with antibiotics and/or oral corticosteroids. A minimum of 2 weeks between consecutive hospitalisations/start of new therapy is necessary to consider events as separate. A modified ATS/ERS operational classification of exacerbation severity will be used for each exacerbation: level I, increased use of short acting β2 agonist; level II, use of oral corticosteroids or antibiotics; level III, care by services to prevent hospitalisation; and level IV, admitted to hospital [38].

##### Hospital admissions

The number of unscheduled hospital admissions whilst on study medication is ascertained at the 26- and 52-week assessments. Emergency COPD admissions will also be identified.

##### Major adverse cardiovascular events (MACE)

MACE as defined by cardiovascular death, hospitalisation for myocardial infarction, heart failure, or stroke, percutaneous coronary intervention or coronary artery bypass grafting will be ascertained at the 26- and 52-week assessments [33].

##### Health-related quality of life

Health-related quality of life data will be captured at recruitment, 26 and 52 weeks using EuroQoL 5D-5L (EQ-5D-5L) Index that has been used widely in studies of COPD [35]. EQ-5D-5L was developed as a utility questionnaire and addresses mobility, self-care, usual activities, pain/discomfort and anxiety/depression. The completed instrument can be translated into quality of life utilities suitable for calculation of QALYs through the published UK tariffs [39].

##### Disease-related health status

Disease-related health status will be ascertained at recruitment and at the 26- and 52-week assessments by questionnaire using the COPD Assessment Test (CAT) [36]. The CAT is an 8-item unidimensional measure of health status impairment in COPD. The score ranges from 0 to 40; it correlates very closely with health status measured using the St George Respiratory Questionnaire and is reliable and responsive. The CAT score is preferred since it provides a more comprehensive assessment of the symptomatic impact of COPD [40, 41].

The Baseline Dyspnoea Index (BDI) questionnaire will be administered at the recruitment assessment and the Transitional Dyspnoea Index (TDI) will be administered at the 26- and 52-week assessments [42]. BDI and TDI were developed in order to obtain a comprehensive understanding of patients’ severity of breathlessness and are based on three components: functional impairment, magnitude of task and magnitude of effort. BDI is a discriminative instrument used to quantify the severity of dyspnoea at an initial or baseline state, whereas TDI is an evaluative instrument used to quantify the changes in dyspnoea from the initial or baseline state [34].

##### Post bronchodilator lung function

Post bronchodilator lung function will be measured by spirometry performed to ATS/ERS standards, at recruitment, titration visits (weeks 1, 2, 3 and 4) and 26 and 52 weeks [43]. However, because of COVID-19, it will not be possible for spirometry to be performed, and this outcome will not be available for many participants.

##### Health care utilisation

Health care utilisation during the previous 6 months is recorded at recruitment and at the 26- and 52-week assessments using a modified version of the Client Service Receipt Inventory (CSRI) [44]. The CSRI is a research questionnaire for retrospectively collecting cost-related information about participant’s use of health and social care services.

##### Adverse reactions and serious adverse events

Adverse reactions and serious adverse events are recorded at each titration assessment, the 26- and 52-week assessments and the end of weaning phone call. Participants are notified of recognised adverse reactions and encouraged to contact the local study centre if they experience these.

##### Mortality

Deaths during the follow-up period are recorded and reported as serious adverse events.

##### Adherence

Adherence with study treatment is assessed at each assessment by asking participants to estimate their adherence.

##### Echocardiography

Participants in the cardiac sub-study undergo echocardiography as early as possible during the titration period subject to logistics and the processes of informed consent. Echocardiography is performed according to standard protocols on GE or Philips systems, inclusive of diastolic function, speckle and tissue Doppler imaging for offline analysis [45]. Apical 2, 3 and 4 chamber views are acquired for accurate computation of ejection fraction, atrial volumes, left ventricular wall thickness and estimated pulmonary artery pressure. The images are promptly sent to the University of Aberdeen, Department of Cardiology, for analysis. Any significant or clinically relevant findings that are likely to significantly impact on the patient’s health or future prognosis will be provided to the participant’s GP (with the participant’s agreement).

##### Blood assays

Participants in the cardiac sub-study have a venous blood sample taken as early as possible during the titration period and at the final 52-week assessment. These samples will be assayed for high-sensitivity cardiac troponin I (hs-cTnI), N-terminal pro-Brain Natriuretic Peptide (NT-proBNP), Galectin and high-sensitivity C-reactive protein (hs-CRP) by laboratories at the Universities of Dundee and Edinburgh. Hs-cTnI is a biomarker of myocardial damage and NT-proBNP is released in response to changes in intra cardiac pressure [44].

#### Plans to promote participant retention and complete follow-up {18b}

To promote retention, participants unable to take part in the 26- and 52-week assessments will be sent the questionnaire to complete at home. Participants who cease taking the study medication will be invited to participate in the 26- and 52-week assessments. For participants who do not participate in the 52-week assessment and who do not complete the questionnaire, attempts will be made to identify the number of exacerbations in the appropriate time period by examining GP records; if possible, MACE outcomes will be determined at the same time.

#### Data management {19}

At baseline and at follow-up assessments, study data can be entered directly into the study website maintained by CHaRT and held on a secure server; the study website has a full audit trail. Sites that directly enter data into the website are encouraged to print or save a copy of the electronic data in order to maintain a copy of the data independent of that held by the sponsor. Study data collected on hard copy case report forms are subsequently entered into the study website. The central trials team monitor data entry and ensure that missing or implausible data are addressed as soon as possible after detection. All study documentation will be archived for at least 25 years after publication of the study data.

#### Confidentiality {27}

All investigators and study site staff involved comply with the requirements of GCP and the UK Data Protection Acts 2018. Data will be stored for at least 25 years after publication. Publications will not contain any personal data that could allow identification of individual participants.

#### Plans for collection, laboratory evaluation and storage of biological specimens for genetic or molecular analysis in this trial/future use {33}

Details about the collection of the blood samples and echocardiography are described in the ‘Plans for assessment and collection of outcomes {18a}’ section. Any residual material will be retained in an approved tissue bank.

### Statistical methods

#### Statistical methods for primary and secondary outcomes {20a}

Statistical analyses will be conducted in accordance with the intention-to-treat principle with a per-protocol analysis performed as a sensitivity. The per-protocol analysis will exclude participants who were not compliant (at less than 70%) with their study medication. All analyses will be governed by a comprehensive statistical analysis plan that will be finalised before the data lock.

The primary clinical outcome of number of COPD exacerbations will be compared between randomised groups using negative binomial regression with length of time in the study as an offset. Estimates will be adjusted for centre and other baseline covariates known to be related to outcome (e.g. age, smoking, COPD hospitalisations in year prior to study, medications). An over dispersion parameter will be used to adjust for between patient variability. To assess the impact of death (estimated at around 6%) [46], a sensitivity analysis will be undertaken by excluding those subjects who have died. For participants that are lost to follow-up (estimated to be around 15%) [46], their information will be included in the statistical models up to the point that they are lost to follow-up. Sensitivity analyses will be undertaken using multiple imputation (assuming data are missing at random), and, if necessary, and the data permit, specify the mechanism of missing data via a pattern mixture model assuming informative missingness.

The secondary outcomes—total number of COPD exacerbations requiring hospital admission and total number of emergency hospital admissions (all causes)—will be analysed in the same way as for the primary outcome. Mixed effects models will be used to compare the secondary outcomes, CAT, EQ5D-5 L, FEV_1_ and FVC, by randomisation group adjusted for centre, patient characteristics and/or baseline clinical variables. All-cause mortality rate and time to first COPD exacerbation will be compared between randomised group using a log-rank test and Kaplan-Meier survival curves. Adjustment for potential covariates will be undertaken using Cox proportional hazards regression. Major adverse cardiovascular events (MACE) in randomisation groups will be compared using the chi-square test.

A National Health Service perspective will be adopted in keeping with the NICE reference case for health technology assessments [47]. The two-stage economic evaluation will comprise an analysis of treatment cost-effectiveness within trial period and then extrapolated to lifetime using cost-effectiveness modelling. The within trial analysis will use health care resource use data (translated to a cost-per-patient using unit costs standard reference sources), the exacerbation rate associated with the treatment groups and the quality of life effects estimated from the EQ-5D-5L combined with length of life to calculate QALYs. Non-parametric bootstrapping will be used to capture sampling uncertainty in the observed data and results will be presented as cost-per-exacerbation avoided and cost-per-QALY gained within the trial period. The extrapolation analysis will make use of regression estimates of exacerbation on cost and quality of life from the trial, as well as previously published models of COPD, to guide the extrapolation to patient lifetimes. In addition to sampling uncertainty, extensive sensitivity, analysis will be performed to understand the importance of alternative modelling assumptions for the extrapolated results.

#### Interim analyses {21b}

There are no planned interim analyses.

#### Methods for additional analyses {20b}

A sub-group analysis of participants with clinically diagnosed heart disease to determine whether any beneficial effects of bisoprolol are limited to those with clinically diagnosed heart disease was performed. An additional sub-group analysis of the primary outcome is planned (including by sex and smoking status).

#### Methods in analysis to handle protocol non-adherence and any statistical methods to handle missing data {20c}

The primary analysis will use the intention-to-treat principle. A per-protocol analysis will include those patients who took 70% or more of their study medication. Sensitivity analysis to handle missing data is described above [20a].

#### Plans to give access to the full protocol, participant level-data and statistical code {31c}

The full protocol is included as supplementary material. Requests for access to participant level data and/or the statistical code should be made in writing to the chief investigator Graham Devereux (graham.devereux@lstmed.ac.uk). Data will be provided after review, approval by investigators and a data transfer agreement has been signed.

### Oversight and monitoring

#### Composition of the coordinating centre and trial steering committee {5d}

The immediate trial team based in the coordinating centre (CI, trial manager, data coordinator) meet weekly initially. The frequency of these meetings is likely to reduce as the trial progresses, but can revert to weekly meetings as and when required. On a monthly basis, the immediate team is joined by a wider team (statistician, health economist). A Project Management Group (PMG) and Trial Steering Group (TSC) oversee the project. The PMG meets approximately every three months and comprises the chief investigators, grant holders (including clinical, methodological, statistical, health economic and qualitative expertise) and the trial office staff. The TSC meet approximately every 6 months and includes an independent chair, clinical and methodological expertise and lay representatives.

#### Composition of the data monitoring committee, its role and reporting structure {21a}

The independent Data Monitoring Committee (DMC) comprises an independent chair and independent members with clinical, statistical and methodological expertise. The DMC meets every 6 months or more frequently if required. The DMC is independent of the sponsor and competing interests and reports to the chair of the Trial Steering Committee.

#### Adverse event reporting and harms {22}

The trial complies with the UK National Health Service National Research Ethics Service guidelines for reporting adverse events [48]. Adverse reactions (ARs) and serious adverse events (SAEs) are recorded from the time a participant consents to join the study until the end of the weaning period after the 52-week follow-up. Participants who withdraw from taking the study drug during the 52-week treatment period have ARs and SAEs recorded from consent until 28 days after ceasing study medication. Exacerbations of COPD or hospital admissions as a consequence of exacerbations of COPD are not considered as ARs or SAEs because they are primary and secondary outcomes for the trial. The Reference Safety Information used in the assessment of SAEs/ARs is based on the SmPC for bisoprolol and is located on the study website [31].

#### Frequency and plans for auditing trial conduct {23}

The trial office monitor aspects of the study on an ongoing basis as described in the study monitoring plan prepared at the outset of the study. The trial is also monitored and audited by the Sponsor. Individual sites may be monitored by their local Research and Development department.

#### Plans for communicating important protocol amendments to relevant parties {25}

Changes to the protocol require the trial office to seek permission from the funder, sponsor, REC and NHS R&D offices.

#### Dissemination plans {31a}

A summary of the study findings will be sent to surviving trial participants, their GPs and investigators. The clinical study report will be used for publication and presentation at scientific meetings and at patient and clinical interest group events. The publication policy is included in the protocol supplementary file.

## Discussion

There is a pressing need to identify interventions that reduce the impact of COPD, a common disease that continues to be associated with high morbidity, mortality and healthcare costs. BICS is a pragmatic randomised double-blind placebo-controlled trial that tests the hypothesis that adding the beta blocker bisoprolol to routine COPD treatment reduces the rate of exacerbation. The cardiac sub-study tests the hypothesis that any beneficial effect of bisoprolol is restricted to those with unrecognised heart disease.

Recruitment to the BICS trial was paused in March 2020 due to the COVID-19 pandemic and remained paused until July 2021 when the sponsor and funder agreed that the study could re-open to recruitment. As part of the plans for re-opening, a number of revisions were made to the protocol.

Firstly, we reduced the number of face to face assessments. When the study first opened to recruitment in 2018, there were up to 7 face-to-face assessments (baseline, four titration visits, follow-up at 26 and 52 weeks). In response to COVID-19, the number of face-to-face assessments for the main BICS study was reduced to one baseline assessment; although if the site or participant prefer to carry out the baseline assessments remotely (by telephone or videocall), this is permitted. Those participants who wish to take part in the sub-study will have two additional face-to-face assessments, one soon after recruitment for the blood samples and echocardiogram, and one around the 52-week follow-up for blood samples.

Secondly, we amended the inclusion criteria. When the study first opened to recruitment, one of the inclusion criteria was “at least two exacerbations requiring treatment with antibiotics and/or oral corticosteroid use in the previous year, based on patient report,” and this was revised so that the two exacerbations could have occurred since March 2019, providing they were within 12 months of each other. The number of exacerbations experienced by people with COPD declined during the COVID-19 pandemic probably because of COVID-19 mitigation measures, e.g. shielding, social distancing measures and face coverings [49]. The ECLIPSE study has demonstrated that the frequent-exacerbation phenotype is relatively stable over a 3-year period, and the strongest predictor for further exacerbations is a history of exacerbations [37]. The change in inclusion criteria regarding the timing of previous exacerbations enables the identification of the people who remained at high risk of exacerbation even during the time of reduced exacerbation risk. We also amended the inclusion criteria to remove the requirement for spirometry (which was classed as an aerosol generating procedure and thus restricted within the NHS). When the study first opened to recruitment, the use of current or historical evidence of FEV_1_/FVC < 0.7 was permitted but FEV_1_ < 80% predicted needed to be demonstrated at baseline. The inclusion criteria were amended so that both FEV_1_/FVC < 0.7 and FEV_1_ < 80% predicted are based on historical readings recorded in the medical records.

Thirdly, we amended the titration schedule within the study. When the study first opened to recruitment, the titration schedule included FEV_1_. Again, because of the limitations on spirometry in the NHS, the titration schedule was revised so that FEV_1_ was replaced with participant self-report of their breathing—with a particular focus on deterioration since the study medication was started or the dose of study medication was increased.

Beta blockers are predominantly used in the management of cardiovascular conditions e.g. hypertension, heart failure; however, it has been observed that beta blocker use in people with COPD is associated with a reduced risk of exacerbation [18–24]. A systematic review of observational cohort studies of beta blocker use for cardiovascular conditions demonstrated that beta blocker use in people with COPD was associated with reduced mortality and exacerbation rate [18]. The BICS trial is one of the first to investigate whether commencing people on a beta blocker for their COPD is beneficial. The cardiac sub-study will investigate whether any beneficial effect of bisoprolol is limited to those people with COPD with unrecognised heart disease as determined by risk stratification based on echocardiography and blood concentrations of Galectin, hs-cTnI, hs-CRP and NTproBNP [45].

The sample size for current study was based on the ECLIPSE study reporting that for people with COPD with two or more exacerbations in a year, the mean (SD) number of COPD exacerbations within the subsequent one year was 2.22 (1.86). In our trial of low-dose theophylline in COPD (TWICS), for people with COPD with two or more exacerbations in the previous year, the mean (95% CI) number of exacerbations in the subsequent year was 2.23 (2.09, 2.37). The TWICS study also confirmed reports that patient recall of COPD exacerbations is reliable over a year with 79% concordance between participant and GP clinical records of exacerbation in a validation exercise [46, 50].

BICS is one of several trials investigating the role of beta blockers in people with COPD. BLOCK COPD was a multicentre randomised double blind placebo controlled trial of metoprolol in people with moderate/severe COPD (FEV_1_ < 80% predicted) conducted in the USA (protocol NCT02587351) [51]. Participants were randomised 1:1 to extended release metoprolol succinate or placebo with a 6-week dose titration phase resulting in final doses of 25 mg, 50 mg or 100 mg od, the total treatment period was 52 weeks. The inclusion criteria included ≥ 1 exacerbation in the previous year treated with antibiotics and/or systemic corticosteroids or, to be using, or have been prescribed supplemental home oxygen for at least 12 h a day. The primary outcome was the time to the first moderate/severe COPD exacerbation, the study sample size of 1028 was powered to detect a 15% reduction in the probability of an exacerbation in the year treatment period. This trial was terminated prematurely after recruitment of 532 participants because of futility with respect to the primary end point and safety concerns. There was no significant between group difference in the median time to first exacerbation (202 days metoprolol, 222 days placebo group). Although metoprolol was associated with a higher risk of exacerbation leading to hospitalisation (hazard ratio, 1.91; 95% CI, 1.29 to 2.83), in the year prior to commencing study drug, the metoprolol group were more likely to have a COPD exacerbation leading to emergency department visit or hospitalisation (62.7% vs 50.4%, *p* = 0.004). The BICS trial differs from BLOCK COPD in several important respects. In BICS the beta blocker bisoprolol is being tested because, unlike metoprolol, it is licensed for use in heart failure in the UK and has a higher beta1:beta2 receptor selectivity ratio (14:1) than metoprolol (2:1) [52]. In BLOCK COPD, potential participants had an electrocardiograph (ECG) recording and were rendered ineligible by prespecified ECG abnormalities. The pragmatic design of BICS replicates the routine practice of commencing beta blockers in UK primary care settings without an ECG; thus, when compared with BLOCK COPD, the participants in BICS are likely to represent COPD patients in the UK NHS, to have more unrecognised heart disease and benefit from beta blocker therapy. A study of bisoprolol in preventing adverse cardiac events in COPD in Australia and New Zealand (protocol NCT03917914) commenced recruitment in June 2020. In this multicentre randomised double-blind placebo controlled trial, 1164 people with COPD (FEV_1_ < 70% predicted) with ≥ 1 exacerbation in the previous year will be randomised 1:1 to bisoprolol or placebo, the treatment period is 2 years and the doses of bisoprolol will be 1.25 mg, 2.5 mg or 5 mg od. The primary outcomes include all-cause mortality, hospitalisation for COPD exacerbation, hospitalisation for primary cardiac cause and major adverse cardiovascular events. A single-arm open label trial of bisoprolol in people with moderate/severe COPD (FEV_1_ < 80% predicted) and hypertension conducted in Japan (protocol UMIN000024712) appears to have ceased recruitment [53]. A total of 35 participants were to be recruited and commenced on bisoprolol 1.25 mg od, increasing monthly to 2.5 mg od and 5 mg od if tolerated; the primary outcome was the rate of moderate-to-severe COPD exacerbation during the 2-year treatment period. The study sample size was powered to detect a 25% reduction in rate of exacerbation. BRONCHOLE is a multicentre randomised open label controlled trial of metoprolol in people with COPD being conducted in the Sweden (protocol NCT03566667) [54]. All participants will be randomised 1:1 to metoprolol or standard care, the target metoprolol dose is 100 mg od and the treatment period is 52 weeks. There are no exacerbation inclusion criteria and potential participants are excluded if their records contain any International Classification of Diseases (ICD) codes for cardiovascular diseases. The primary outcome is a composite of all-cause mortality, COPD exacerbations and cardiovascular events, the study sample size of 1700 is powered to detect a 25% reduction in the primary outcome; however, this outcome is dominated by exacerbations. This trial is due to finish recruitment in December 2021.

It is almost certain that a substantial proportion of the participants in BICS will have severe lung disease and will have limited exercise tolerance. When the study first opened to recruitment, allowances were made in the trial design to facilitate participation by this group of patients: at site discretion, participants could be recruited and dose titrated home using portable spirometers and sphygmomanometers, and those unable to attend for follow-up assessment visits would be assessed by telephone review and postal collection of quality of life questionnaires. We hope that the revisions made to the protocol to allow restart have made the study even more accessible to this group of patients. Study medication is to be couriered directly to the homes of participants, thus avoiding travel to study centres to collect supplies.

The dose each participant takes for the duration of the study is determined by titrating the dose of study drug during the first 4 weeks of the treatment period; however, to increase flexibility and to replicate ‘real life’, there is sufficient medication in the first batch of medication for the dose titration to be conducted over 7 weeks. The study drug is provided as 1.25 mg bisoprolol tablets; the dose titration results in final doses of 1, 2, 3 or 4 tablets a day. A single tablet was chosen to avoid the complications of different tablet strengths and doses. The dose titration schedule is a conservative interpretation of the ‘start low, go slow’ advice provided in heart failure guidelines designed for use by appropriately trained nurses in primary care settings [29, 30]. Decisions to increase, reduce or to fix on a dose during the titration period are determined by participant reports of intolerable side effects, heart rate, systolic blood pressure and changes in breathing (replaced the pre COVID-19 lung function criterion). The algorithm is included in the study website and requires research staff to enter these parameters, and the programme provides an advisory recommendation; at all times, clinicians are free to ignore the advice and to make their own clinical decision.

Bisoprolol is a relatively cheap drug familiar to many clinicians whose use is established in people with heart failure and COPD. The demonstration that the addition of bisoprolol to routine COPD treatment reduces the likelihood of exacerbation will be relevant not only to patients and clinicians but also to healthcare providers, both in the UK, and globally.

## Trial status

The current protocol is version 7, 14 May 2021. The first participant was recruited on 16 October 2018. The trial was paused to recruitment in March 2020 due to the COVID-19 pandemic. It remained paused to recruitment until July 2021 when the sponsor and funder agreed that the study could re-open to recruitment. BICS is currently recruiting patients until 31 March 2022 by which point discussions with the funder regarding study viability in relation to COVID-19 and recruitment will have been completed.

## Supplementary Information

**Additional file 1.**

## Abbreviations

BDI: Baseline dyspnoea index
BICS: Bisoprolol in COPD study
CAT: COPD Assessment Test
CHaRT: Centre for Healthcare Randomised Trials
COPD: Chronic Obstructive Pulmonary Disease
FEV_1_: Forced expiratory volume in first second
FVC: Forced vital capacity
hs-cTnI: High-sensitivity cardiac troponin I
NICE: National Institute for Health and Care excellence
NTproBNP: N-terminal pro-Brain Natriuretic Peptide
QALY: Quality-adjusted life year
TDI: Transition dyspnoea index
£: UK pound sterling
