# Supplementary material for: Use of the oral beta blocker bisoprolol to reduce the rate of exacerbation in people with chronic obstructive pulmonary disease (COPD): a randomised controlled trial (BICS)
Source: Trials. 2022 Apr 14;23:307. doi: 10.1186/s13063-022-06226-8 (PMC9009490; doi:10.1186/s13063-022-06226-8)
Supplement: Supplementary file 1 — Additional file 1. [file 13063_2022_6226_MOESM1_ESM.pdf]

**A randomised, double-blind placebo controlled trial of the effectiveness of the beta-blocker bisoprolol in preventing exacerbations of chronic obstructive pulmonary disease.**

BICS

Bisoprolol in COPD Study

| | |
|---|---|
| **Chief Investigator:** | Professor Graham Devereux |
| **Sponsor:** | University of Aberdeen / NHS Grampian |
| **Sponsor Reference Number:** | 3.089.17 |
| **IRAS Number:** | 242190 |
| **REC Reference:** | 18/SS/0033 |
| **EudraCT Number:** | 2017-002779-24 |
| **ISRCTN Number:** | 10497306 |
| **Funder:** | NIHR Health Technology Assessment |
| **Funding Reference Number:** | 15/130/20 |
| **BICS trial office:** | Email: bics@abdn.ac.uk<br>Telephone: 01224 438178 |
| **Version number:** | Version 7, 14 May 2021 |

This protocol has regard for the HRA guidance and order of content (based on HRA version 1.2, March 2016)

## SIGNATURE PAGE

The undersigned confirm that the following protocol has been agreed and accepted and that the Chief Investigator agrees to conduct the trial in compliance with the approved protocol and will adhere to the principles outlined in the Medicines for Human Use (Clinical Trials) Regulations 2004 (SI 2004/1031), amended regulations (SI 2006/1928) and any subsequent amendments of the clinical trial regulations, GCP guidelines, the Sponsor's (and any other relevant) SOPs, and other regulatory requirements as amended.

I agree to ensure that the confidential information contained in this document will not be used for any other purpose other than the evaluation or conduct of the clinical investigation without the prior written consent of the Sponsor

I also confirm that I will make the findings of the trial publicly available through publication or other dissemination tools without any unnecessary delay and that an honest accurate and transparent account of the trial will be given; and that any discrepancies and serious breaches of GCP from the trial as planned in this protocol will be explained.

**For and on behalf of the Trial Sponsor:**

Signature: ..........................[redacted]..................... Date: 16/07/2021

Name (please pri[redacted]  Position:

Louise King  Research Governance Manager

**Chief Investigator:**

Signature: ........................................................................................ Date: ....../....../......

Name: (please print): ........................................................................................

Position: .....................................................................

**Statistician:**

Signature: ......................[redacted].................... Date: 21/07/2021

Name: (please print):  Position:

Amanda Lee  Professor of Medical Statistics

**Clinical Trials Pharmacist:**

Signature: ........................................................................................ Date: ....../....../......

Name (please print): ........................................................................................

Position: .....................................................................

## SIGNATURE PAGE

The undersigned confirm that the following protocol has been agreed and accepted and that the Chief Investigator agrees to conduct the trial in compliance with the approved protocol and will adhere to the principles outlined in the Medicines for Human Use (Clinical Trials) Regulations 2004 (SI 2004/1031), amended regulations (SI 2006/1928) and any subsequent amendments of the clinical trial regulations, GCP guidelines, the Sponsor's (and any other relevant) SOPs, and other regulatory requirements as amended.

I agree to ensure that the confidential information contained in this document will not be used for any other purpose other than the evaluation or conduct of the clinical investigation without the prior written consent of the Sponsor

I also confirm that I will make the findings of the trial publicly available through publication or other dissemination tools without any unnecessary delay and that an honest accurate and transparent account of the trial will be given; and that any discrepancies and serious breaches of GCP from the trial as planned in this protocol will be explained.

**For and on behalf of the Trial Sponsor:**

Signature: ............................................................ Date: ....../....../......

Name (please print): ............................................................ Position: ............................................................

**Chief Investigator:**

Signature: [redacted] Date: 16/ 7/ 21

Name: (please print): GRAHAM DEVEREUX Position: CHIEF INVESTIGATOR.

**Statistician:**

Signature: ............................................................ Date: ....../....../......

Name: (please print): ............................................................ Position: ............................................................

**Clinical Trials Pharmacist:**

Signature: ............................................................ Date: ....../....../......

Name (please print): ............................................................ Position: ............................................................

**SIGNATURE PAGE**

The undersigned confirm that the following protocol has been agreed and accepted and that the Chief Investigator agrees to conduct the trial in compliance with the approved protocol and will adhere to the principles outlined in the Medicines for Human Use (Clinical Trials) Regulations 2004 (SI 2004/1031), amended regulations (SI 2006/1928) and any subsequent amendments of the clinical trial regulations, GCP guidelines, the Sponsor's (and any other relevant) SOPs, and other regulatory requirements as amended.

I agree to ensure that the confidential information contained in this document will not be used for any other purpose other than the evaluation or conduct of the clinical investigation without the prior written consent of the Sponsor

I also confirm that I will make the findings of the trial publicly available through publication or other dissemination tools without any unnecessary delay and that an honest accurate and transparent account of the trial will be given; and that any discrepancies and serious breaches of GCP from the trial as planned in this protocol will be explained.

**For and on behalf of the Trial Sponsor:**

Signature: ............................................................................... Date: ....../....../......

Name (please print): ............................................................................... Position: ...............................................................................

**Chief Investigator:**

Signature: ............................................................................... Date: ....../....../......

Name: (please print): ............................................................................... Position: ...............................................................................

**Statistician:**

Signature: ............................................................................... Date: ....../....../......

Name: (please print): ............................................................................... Position: ...............................................................................

**Clinical Trials Pharmacist:**

Signature: ..........[redacted]............................................ Date: 27/07/2021

Name (please print): PATRICIA COOPER............................................ Position: LEAD CLINICAL TRIALS PHARMACIST



coopep1 27/07/2021 09:07

## KEY TRIAL CONTACTS

| | |
|---|---|
| Chief Investigator | Dr Graham Devereux<br>Professor of Respiratory Medicine,<br>c/o CHaRT, 3rd Floor, Health Sciences Building,<br>Foresterhill, Aberdeen. AB25 2ZD<br>Telephone: 01224 438178<br>E-mail: g.devereux@abdn.ac.uk |
| Trial Manager | Dr Seonaidh Cotton<br>Trials Manager,<br>CHaRT, 3rd Floor, Health Sciences Building,<br>Foresterhill, Aberdeen. AB25 2ZD<br>Telephone: 01224 438178<br>E-mail: s.c.cotton@abdn.ac.uk |
| Joint-sponsor(s)/co-sponsor(s) | University of Aberdeen (Dr Rattray)<br>NHS Grampian (Professor Cruickshank)<br><br>Sponsor contact:<br>Ms Louise King<br>Research Governance Manager,<br>Polwarth Building, Foresterhill, Aberdeen. AB25 2ZD<br>Telephone: 01224437221<br>E-mail: researchgovernance@abdn.ac.uk |
| Funder(s) | National Institute for Health Research, Health Technology Assessment |
| Clinical Trials Unit | Centre for Healthcare Randomised Trials (CHaRT)<br>3rd Floor, HSRU, Health Sciences Building,<br>Foresterhill, Aberdeen. AB25 2ZD<br>Telephone 01224 438140 |

| | |
|---|---|
| Key Protocol Contributors | Dr Brian Lipworth<br>Professor of Respiratory Medicine,<br>Scottish Centre for Respiratory Research,<br>Ninewells Hospital, Dundee. DD2 1UB<br>Telephone 01382 383188<br>E-mail: b.j.lipworth@dundee.ac.uk<br><br>Prof Andy Briggs<br>Chair in Health Policy & Economic Evaluation,<br>Health Economics & Health Technology Assessment,<br>Centre for Population & Health Sciences,<br>1 Lilybank Gardens, Glasgow. G12 8RZ<br>Telephone: 0141 330 5017<br>E-mail: Andrew.Briggs@glasgow.ac.uk<br><br>Prof Rekha Chaudhuri<br>Associate Professor and Associate Specialist,<br>University of Glasgow, Gartnavel General Hospital,<br>1053 Great Western Road, Glasgow. G12 0YN<br>Telephone: 0141 211 1673<br>E-mail: Rekha.Chaudhuri@ggc.scot.nhs.uk<br><br>Dr Seonaidh Cotton<br>Trials Manager,<br>CHaRT, 3rd Floor, Health Sciences Building,<br>Foresterhill, Aberdeen. AB25 2ZD<br>Telephone: 01224 438178<br>E-mail: s.c.cotton@abdn.ac.uk |

| | |
|---|---|
| | Dr Anthony De Soyza<br>HEFCE Academic Clinical Senior Lecturer,<br>Medical School, Framlington Place,<br>Newcastle Upon Tyne. NE2 4HH<br>Telephone: 0191 213 7468<br>E-mail: anthony.de-soyza@newcastle.ac.uk<br><br>Dr Shona Fielding<br>Institute of Applied Health Sciences,<br>Polwarth Building, Foresterhill, Aberdeen. AB25 2ZD<br>Telephone 01224 437107<br>E-mail: s.fielding@abdn.ac.uk<br><br>Dr Simon Gompertz<br>Department of Respiratory Medicine,<br>Queen Elizabeth Hospital, Birmingham, B15 2WB.<br>Telephone: 01213714826<br>E-mail: Simon.Gompertz@uhb.nhs.uk<br><br>Dr John Haughney<br>Research Fellow,<br>Centre of Academic Primary Care, Polwarth Building,<br>Foresterhill, Aberdeen. AB25 2ZD<br>Telephone: 01224 553066<br>E-mail: john.haughney@btinternet.com<br><br>Prof Amanda Lee<br>Professor of Medical Statistics,<br>Institute of Applied Health Sciences,<br>Polwarth Building, Foresterhill, Aberdeen. AB25 2ZD<br>Telephone 01224 437111<br>E-mail: a.j.lee@abdn.ac.uk<br><br>Mr Alister Laird,<br>c/o CHaRT, 3rd Floor, Health Sciences Building,<br>Foresterhill, Aberdeen. AB25 2ZD<br>Telephone: 01224 438178<br><br>Prof William MacNee<br>MRC Centre for Inflammation Research,<br>The Queen's Medical Research Institute,<br>47 Little France Crescent, Edinburgh. EH16 4TJ<br>Telephone: 0131 2426581<br>E-mail: w.macnee@ed.ac.uk<br><br>Prof Alyn Morice,<br>Head of Cardiovascular and Respiratory Studies,<br>Castle Hill Hospital, Hull. HU16 5JQ<br>Telephone: 01482 624066<br>E-mail: A.H.Morice@hull.ac.uk<br><br>Prof John Norrie<br>Director, Edinburgh Clinical Trials Unit,<br>University of Edinburgh, Western General Hospital,<br>Crewe Road South, Edinburgh. EH4 2XU<br>Telephone: 0131 537 3850<br>E-mail: j.norrie@ed.ac.uk |

| | |
|---|---|
| | Prof David Price<br>Professor of Primary Care Respiratory Medicine,<br>Respiratory Medicine,<br>Centre of Academic Primary Care, Polwarth Building,<br>Foresterhill, Aberdeen. AB25 2ZD<br>Telephone: 02081233923<br>E-mail: david@respiratoryresearch.org<br><br>Dr Philip Short<br>Consultant Respiratory Physician<br>Honorary Senior Lecturer (University of Dundee)<br>Perth Royal Infirmary, Perth. PH1 1NX<br>Telephone: 01382 660111.<br>E-mail: P.Short@dundee.ac.uk<br><br>Prof Jorgen Vestbo<br>Professor of Respiratory Medicine<br>Division of Infection, Immunity and Respiratory Medicine University of Manchester Education and Research Centre University Hospital of South Manchester, Manchester. M23 9LT<br>Telephone: 0161 291 5869<br>E-mail: Jorgen.Vestbo@manchester.ac.uk<br><br>Dr Paul Walker,<br>Consultant Physician,<br>Aintree Chest Centre, University Hospital Aintree,<br>Lower Lane, Liverpool, L9 7AL<br>Telephone: 0151 529 3796<br>E-mail: ppwalker@liverpool.ac.uk<br><br>Professor Jadwiga Wedzicha,<br>Professor of Respiratory Medicine,<br>Airways Disease Section, National Heart and Lung Institute, Imperial College London, Guy Scadding Building,<br>Dovehouse Street, London SW3 6LY<br>Telephone: 0207 594 7947<br>E-mail: j.wedzicha@imperial.ac.uk<br><br>Prof Andrew Wilson,<br>Professor of Respiratory Medicine,<br>Airway Inflammation Research Group, Biomedical Research Centre,<br>School of Medicine, University of East Anglia, Norwich. NR4 7TJ<br>Telephone: 01603 591257<br>E-mail: A.M.Wilson@uea.ac.uk |
| Statistician | Dr Shona Fielding,<br>Institute of Applied Health Sciences,<br>Polwarth Building, Foresterhill, Aberdeen. AB25 2ZD<br>Telephone 01224 437107<br>E-mail s.fielding@abdn.ac.uk |
| Trials Pharmacist | Mrs Patricia Cooper<br>Clinical Trials Pharmacy<br>Aberdeen Royal Infirmary<br>Aberdeen. AB25 2ZN<br>E-mail: patricia.cooper@nhs.scot<br>Tel 01224 553624<br>Fax 01224 551061 |

| | |
|---|---|
| Committees | **Trial Steering Committee**<br>Chair: Prof Edwin Chilvers<br>Head of the National Heart and Lung Institute (NHLI)<br>Imperial College London<br>Room 540, Hammersmith Campus<br>Du Cane Road<br>London, W12 0NN<br><br><br>Dr Matthew Sydes<br>Reader in Clinical Trials<br>MRC Clinical Trials Unit @ UCL<br>Aviation House, 125 Kingsway<br>London. WC2B 6NH<br>E-mail: m.sydes@ucl.ac.uk<br><br>Dr Rod Lawson<br>Consultant in Respiratory Medicine<br>Clinical Lead for Chronic Obstructive Pulmonary Disease<br>Northern General Hospital Herries Road, Sheffield, S5 7AU<br>E-mail: Rod.Lawson@sth.nhs.uk<br><br>Note: Dave Bertin is no longer employed by CHSS. Alister Laird passed away in July 2020. We are seeking new PPI input into the study.<br><br><br>**Data Monitoring Committee**<br>Chair: Prof Mike Thomas<br>Professor of Primary Care Research<br>University of Southampton,<br>Aldermoor Health Centre, Aldermoor Close, Southampton SO16 5ST<br>E-mail: D.M.Thomas@soton.ac.uk<br><br>Dr Stephen Gerry<br>Centre for Statistics in Medicine University of Oxford Botnar Research Centre Windmill Road, Oxford. OX3 7LD<br>E-mail: stephen.gerry@csm.ox.ac.uk<br><br>Prof Michael Steiner<br>Department of Infection, Immunity and Inflammation<br>Institute for Lung Health<br>Department of Respiratory Medicine<br>Glenfield Hospital, Groby Road, Leicester. LE3 9QP<br>E-mail: michael.steiner@uhl-tr.nhs.uk |

## i. LIST of CONTENTS

**SIGNATURE PAGE** ........ 2
**KEY TRIAL CONTACTS** ........ 3
**i. LIST of CONTENTS** ........ 7
**ii. LIST OF ABBREVIATIONS** ........ 10
**iii. TRIAL SUMMARIES** ........ 12
**Synopsis** ........ 12
**Lay summary** ........ 15
**Scientific summary** ........ 16
**iv. FUNDING AND SUPPORT IN KIND** ........ 18
**v. ROLE OF TRIAL SPONSOR AND FUNDER** ........ 18
**vi. ROLES AND RESPONSIBILITIES OF TRIAL MANAGEMENT COMMITEES/GROUPS & INDIVIDUALS** ........ 18
**vii. PROTOCOL CONTRIBUTORS** ........ 18
**viii. KEY WORDS:** ........ 18
**ix. TRIAL FLOW CHART** ........ 19
**1 BACKGROUND** ........ 20
**2 RATIONALE** ........ 20
**2.1 Assessment and management of risk** ........ 22
**3 OBJECTIVES AND OUTCOME MEASURES/ENDPOINTS** ........ 23
**3.1 Primary objective** ........ 23
**3.2 Secondary objectives** ........ 23
**3.3 Primary endpoint/outcome** ........ 23
**3.4 Secondary endpoints/outcomes** ........ 24
**4 TRIAL DESIGN** ........ 24
**5 TRIAL SETTING** ........ 25
**6 PARTICIPANT ELIGIBILITY CRITERIA** ........ 25
**6.1 Inclusion criteria** ........ 25
**6.2 Exclusion criteria** ........ 26
**7 TRIAL PROCEDURES** ........ 27
**7.1 Participant identification and Recruitment** ........ 27
**7.1.2 Screening** ........ 28
**7.1.3 Payment** ........ 29
**7.2 Consent** ........ 29
**7.2.1 Additional consent provisions for collection and use of participant data and biological specimens in ancillary studies, if applicable** ........ 30
**7.2.2 Participant safety** ........ 30
**7.3 Randomisation** ........ 30
**7.3.1 Method of implementing the randomisation/allocation sequence** ........ 31
**7.4 Blinding** ........ 31
**7.5 Emergency unblinding** ........ 31
**7.6 Trial assessments** ........ 32
**7.8 Long term follow-up assessments** ........ 36
**7.9 Qualitative assessments** ........ 36
**7.10 Withdrawal criteria** ........ 36
**7.11 Storage and analysis of clinical samples** ........ 37
**7.12 End of trial** ........ 37
**8 TRIAL TREATMENTS** ........ 37
**8.1 Name and description of investigational medicinal product(s)** ........ 37
**8.2 Regulatory status of the drug** ........ 37
**8.3 Product Characteristics** ........ 38

**8.4 Drug storage and supply** ........ 38
**8.5 Preparation and labelling of Investigational Medicinal Product** ........ 39
**8.6 Dosage schedules** ........ 39
**8.7 Dosage modifications** ........ 40
**8.7.1 Dose titration** ........ 40
**8.7.2 Dose reduction at end of follow-up** ........ 41
**8.8 Known drug reactions and interaction with other therapies** ........ 43
**8.9 Concomitant medication** ........ 44
**8.10 Trial restrictions** ........ 44
**8.11 Assessment of compliance with treatment** ........ 44
**8.12 Name and description of each Non-Investigational Medicinal Product (NIMP)** ........ 44
**9 PHARMACOVIGILANCE** ........ 45
**9.1 Definitions** ........ 45
**9.2 Operational definitions for (S)AEs** ........ 46
**9.3 Recording and reporting of SAEs, SARs AND SUSARs** ........ 46
**9.4 Responsibilities** ........ 47
**9.5 Notification of deaths** ........ 48
**9.6 Pregnancy reporting** ........ 48
**9.6.1 Contraceptive requirements and pregnancy testing** ........ 48
**9.7 Overdose** ........ 49
**9.8 Reporting urgent safety measures** ........ 49
**9.9 The type and duration of the follow-up of participants after adverse reactions.** ........ 49
**9.10 Development safety update reports** ........ 50
**9.11 Incidental findings** ........ 50
**10 STATISTICS AND DATA ANALYSIS** ........ 50
**10.1 Sample size calculation** ........ 50
**10.2 Planned recruitment rate** ........ 50
**10.3 Statistical analysis plan** ........ 51
**10.4 Interim analysis and criteria for the premature termination of the trial** ........ 52
**10.5 Economic evaluation** ........ 52
**11 DATA MANAGEMENT** ........ 53
**11.1 Data collection tools and source document identification** ........ 53
**11.2 Data handling and record keeping** ........ 53
**11.3 Access to Data** ........ 54
**11.4 Archiving** ........ 54
**12 MONITORING, AUDIT & INSPECTION** ........ 54
**13 ETHICAL AND REGULATORY CONSIDERATIONS** ........ 54
**13.1 Research Ethics Committee (REC) review & reports** ........ 54
**13.2 Peer review** ........ 55
**13.3 Public and Patient Involvement** ........ 55
**13.4 Regulatory Compliance** ........ 55
**13.5 Protocol compliance** ........ 55
**13.6 Notification of Serious Breaches to GCP and/or the protocol** ........ 55
**13.7 Data protection and patient confidentiality** ........ 56
**13.8 Financial and other competing interests for the chief investigator, PIs at each site and committee members for the overall trial management** ........ 56
**13.9 Indemnity** ........ 56
**13.10 Amendments** ........ 56
**13.11 Post trial care** ........ 57
**13.12 Access to the final trial dataset** ........ 57
**14 DISSEMINATION POLICY** ........ 57

**14.1 Dissemination policy** ... 57
**14.2 Authorship eligibility guidelines and any intended use of professional writers** ... 57
**15 REFERENCES** ... 57
**APPENDICIES** ... 60
**Appendix 1 – Risk** ... 60
**Appendix 2 – Authorisation of participating sites** ... 61
**Appendix 3 – Safety Reporting Flow Chart** ... 62
**Appendix 4 – Authorship policy for the BICS study** ... 63
**Appendix 5 – Amendment History** ... 65
**APPENDIX 6: Protocol for a cardiac sub-study embedded within BICS** ... 68
**APPENDIX 7: Contingency arrangements for COVID-19 (March 2020)** ... 75
**APPENDIX 8: Contingency arrangements for COVID-19 (restart of recruitment)** ... 76

## ii. LIST OF ABBREVIATIONS

| | |
|---|---|
| ACOS | Asthma COPD overlap syndrome |
| AE | Adverse Event |
| AIC | Akaike's Information Criterion |
| AR | Adverse Reaction |
| ATS/ERS | American Thoracic Society / European Respiratory Society |
| BDI | Bassline Dyspnoea Index |
| BLF | British Lung Foundation |
| BP | Blood Pressure |
| bpm | Beats per minute |
| CAT | COPD Assessment Test |
| CHaRT | Centre for Healthcare Randomised Trials |
| CHSS | Chest Heart and Stroke Scotland |
| CI | Chief Investigator |
| COPD | Chronic Obstructive Pulmonary Disease |
| CRF | Case Reporting Form |
| CRN | Clinical Research Network |
| CSRI | The Client Service Receipt Inventory |
| CTA | Clinical Trial Authorisation |
| CTIMP | Clinical Trial of an Investigational Medicinal Product |
| DMC | Data Monitoring Committee |
| DSUR | Development Safety Update Report |
| EC | European Commission |
| EQ-5D-5L | EuroQoL 5 dimensions, 5 level questionnaire |
| EudraCT | European Union Drug Regulating Authorities Clinical Trials |
| FEV1 | Forced expiratory volume-one second |
| FVC | Forced vital capacity |
| GCP | Good Clinical Practice |
| HARQ | The Hull Airways Reflux Questionnaire |
| hs-CRP | high sensitivity C-reactive protein |
| ICS | Inhaled corticosteroids |
| IMP | Investigational Medicinal Product |
| ISF | Investigator Site File (This forms part of the TMF) |
| ISRCTN | International Standard Randomised Controlled Trials Number |
| IVRS | Interactive Voice Randomisation System |
| LABA | Long Acting Beta$_2$ Agonists |
| LAMA | Long Acting Muscrinic Antagonists |
| MACE | Major Adverse Cardiovascular Events |
| MD | Mean Difference |
| mg | milligrams |
| MHRA | Medicines and Healthcare products Regulatory Agency |
| ml | millilitres |
| NHS | National Health Service |
| NHS R&D | National Health Service Research & Development |
| NICE | National Institute for Health and Care Excellence |
| NIHR HTA | National Institute of Health Research Health Technology Assessment |
| NIMP | Non-Investigational Medicinal Product |
| od | Once Daily |
| PI | Principal Investigator |
| PIC | Participant Identification Centre |
| PIL | Patient Information Leaflet |
| QALY | Quality-Adjusted Life-Year |
| R&D | Research and Development |

| | |
|---|---|
| RCT | Randomised Control Trial |
| REC | Research Ethics Committee |
| SABA | Short-acting beta2-agonist |
| SAE | Serious Adverse Event |
| SAP | Statistical analysis plan |
| SAR | Serious Adverse Reaction |
| SmPC | Summary of Product Characteristics |
| SOP | Standard Operating Procedure |
| SUSAR | Suspected Unexpected Serious Adverse Reaction |
| TDI | Transition Dyspnoea Index |
| TMF | Trial Master File |
| TMG | Trial Management Group |
| TSC | Trial Steering Committee |

## iii. TRIAL SUMMARIES

### Synopsis

| | |
|---|---|
| Title | A randomised, double-blind placebo controlled trial of the effectiveness of the beta-blocker bisoprolol in preventing exacerbations of chronic obstructive pulmonary disease. |
| Short title & Acronym | Bisoprolol in COPD study BICS |
| Chief Investigator | Professor Graham Devereux |
| Rationale | Observational studies consistently report that beta-blocker use in people with COPD is associated with a reduced risk of COPD exacerbations. This beneficial association with exacerbations extends to people with COPD without cardiovascular disease and appears to be class specific. This pragmatic prospective double blind randomised trial will formally test whether a beta-blocker reduces the incidence of exacerbations in people with COPD. |
| Objectives | To determine whether the addition of bisoprolol (maximal dose 5mg once a day, or maximum tolerated dose) to usual COPD therapies in patients with COPD at high risk of exacerbation because of a history of at least two COPD exacerbations in a previous year:<br>• Reduces the risk of exacerbation.<br>• Is cost effective to the NHS.<br>• Improves quality of life.<br>• Improves lung function.<br>• Reduces health care use.<br>• Reduces mortality.<br>• Is only beneficial in patients with known heart disease. |
| Trial Configuration | A pragmatic randomised, double-blind, placebo-controlled, multicentre clinical trial. |
| Setting | Both Primary & Secondary Care |
| Sample size estimate | With 669 subjects in each arm of the trial, the trial will be able to detect a 15% reduction in the number of exacerbations in the year of treatment (i.e. from an average of 2.22 to 1.89) with 90% power at the two-sided 5% significance level. Allowing for an estimated 15% withdrawal from treatment with study drug 787 subjects will be recruited in each trial arm: 1,574 in total. |
| Number of participants | 1,574 participants. More than 50% to be recruited from primary care. |
| Eligibility criteria | Inclusion criteria:<br>• Aged ≥ 40 years.<br>• A smoking history of at least 10 pack years ([average number of cigarettes/day x years smoked]/20).<br>• An established diagnosis of COPD (NICE Guideline definition: post bronchodilator $FEV_1$<80% predicted, $FEV_1/FVC$<0.7) receiving treatment as per local guidelines'. During the COVID-19 pandemic, this will be based on historic evidence of $FEV_1$<80% predicted, $FEV_1/FVC$<0.7.<br>• A history of at least two exacerbations requiring treatment with antibiotics and/or oral corticosteroid use in the previous year, based on patient report OR a history of at least two exacerbations within 12 months of each other requiring treatment with antibiotics and/or oral corticosteroid since March 2019.<br>• Clinically stable with no COPD exacerbation for at least 4 weeks. |

<table>
<tr><td></td><td>
<ul>
<li>Able to swallow study medication.</li>
<li>Able and willing to give informed consent to participate.</li>
<li>Able and willing to participate in the study procedures; undergo spirometric assessment (see notes in section 6.1 about cases where spirometry may be contraindicated), complete study questionnaire. During the COVID-19 pandemic, measurement of $FEV_1$ is not required as part of the protocol, and therefore this inclusion criteria does not need to be met.</li>
</ul>
Exclusion Criteria:
<ul>
<li>A current sole respiratory diagnosis of asthma.</li>
<li>Any diagnosis of asthma before the age of 40 years</li>
<li>A predominant respiratory disease other than COPD.</li>
<li>Any significant disease/disorder which, in the investigator’s opinion, either puts the patient at risk because of study participation or may influence the results of the study or the patient's ability to participate in the study.</li>
<li>Previous allocation of a randomisation code in the study or current participation in another interventional study (CTIMP or non-CTIMP).</li>
<li>Current beta-blocker use.</li>
<li>Known or suspected hypersensitivity to beta-blocker.</li>
<li>For women, current pregnancy or breast-feeding, or planned pregnancy during the study.</li>
<li>Heart rate <60 bpm.</li>
<li>Systolic blood pressure <100mmHg.</li>
<li>2nd, 3rd degree heart block.</li>
<li>Diagnosed left heart, heart failure, or within the last year: myocardial infarction, acute coronary syndrome.</li>
<li>Treatment with interacting drugs:<br><u>heart rate limiting drugs:</u> calcium channel blockers (e.g. diltiazem, verapamil), ivabradine,<br><u>class-I antiarrhythmic drugs</u> (e.g. quinidine, disopyramide; lidocaine, phenytoin; flecainide, propafenone),<br><u>centrally-acting antihypertensive drugs</u> (e.g. clonidine methyldopa, moxonidine, rilmenidine).</li>
<li>Severe peripheral arterial disease, severe forms of Raynauds syndrome, myasthenia gravis, periodic hypokalaemic paralysis, pheochromocytoma, thyrotoxicosis and psoriasis.</li>
<li>People without capacity.</li>
</ul>
</td></tr>
<tr><td>Description of interventions</td><td>
Participants will be randomised to either bisoprolol (1.25mg tablets) or an identical placebo.
Starting bisoprolol dose will be one 1.25mg tablet a day. The dose will be increased approximately weekly 1.25mg→2.5mg→3.75mg→5mg resulting in final doses of 1.25mg od (1 tablet), 2.50 mg od (2 tablets), 3.75mg od (3 tablets), or 5mg od (4 tablets) depending on tolerance to bisoprolol up-dosing.
Starting placebo dose will be one tablet a day. The dose will be increased approximately weekly 1 tablet→2 tablets→3 tablets→4 tablets resulting in final doses of 1, 2, 3 or 4 tablets a day depending on tolerance to up-dosing. Further details on titration and tolerance is given in section 8.7.1.
During dose titration, decisions to increase, maintain or reduce the study drug dose will be based on monitoring heart rate, blood pressure, lung function (during the
</td></tr>
</table>

| | |
|---|---|
| | COVID-19 pandemic, lung function will not be assessed, but will be replaced by patient reported changes in breathlessness since dose changed) and clinical status (symptoms, including respiratory symptoms, fatigue).<br>Participants will remain on final titrated dose for remaining 48 weeks.<br>Following completion of the 12 month dosing period participants will be weaned off study drug over following 3 weeks (3-2-1 tablet od). |
| Duration of study | This study will last for 60 months. Each participant will be involved in the study for 12 months. |
| Randomisation and blinding | Determined by a computerised web-based randomisation system created by the University of Aberdeen's Centre for Healthcare Randomised Trials (CHaRT). Participants will be allocated with equal probability to intervention or control arms, stratified by centre (secondary care site, primary care area), and where participants were identified (primary care, secondary care) |
| Outcome measures | Primary outcomes<br>• Total number of exacerbations of COPD necessitating changes in management (use of oral corticosteroids and/or antibiotics) during the one year treatment period.<br>• Cost-per-QALY gained during the one year treatment period.<br><br>Secondary outcomes:<br>• Number of hospital admissions with a primary diagnosis of exacerbation of COPD.<br>• Time to first exacerbation of COPD.<br>• The total number of emergency hospital admissions.<br>• Total number of major adverse cardiovascular events (MACE) (defined by cardiovascular death, hospitalisation for myocardial infarction, heart failure, or stroke), percutaneous coronary intervention or coronary artery bypass grafting.<br>• Adverse events/drug reactions.<br>• Disease related health status using the COPD Assessment Test (CAT).<br>• Health related quality of life using the EQ-5D-5L.<br>• Breathlessness using the Baseline and Transition Dyspnoea Indices (BDI and TDI).<br>• Lung function using spirometry (during the COVID-19 pandemic, lung function cannot be assessed easily and safely and so this outcome will not be available on all participants).<br>• All-cause, respiratory and cardiac mortality.<br>• Incremental cost per exacerbation avoided.<br>• Costs to the NHS and patients and lifetime cost-effectiveness based on extrapolation modelling. |
| Statistical methods | Baseline characteristics will be described for both treatment groups. The primary outcome of number of COPD exacerbations will be compared between randomised groups using negative binomial regression with length of time in the study as an offset. Estimates will then be adjusted for centre and other baseline covariates that are known to be strongly related to the outcome. Sub-group analysis of participants with clinically diagnosed heart disease to determine whether any beneficial effects of bisoprolol are limited to those with clinically diagnosed heart disease. |

## Lay summary

Chronic obstructive pulmonary disease (COPD) is a long term lung disease that slowly gets worse. Although COPD cannot be cured it can be treated. COPD is more common in people from less well-off backgrounds and is strongly linked with smoking. COPD is getting more common, currently about 1.2 million people in the UK are diagnosed with COPD. COPD is the fifth leading cause of death in the UK causing 30,000 deaths a year. Sudden deteriorations in symptoms known as flare ups/exacerbations are an important feature of the disease. During flare ups people get more breathless, more wheezy and start to cough more. Flare ups shorten life expectancy and reduce peoples' ability to get on with their lives. Moreover 60% of the £1billion a year the NHS spends on COPD is taken up treating flare ups.

Beta-blockers are drugs widely used to treat blood pressure and heart disease. Non-heart specialists are very often unwilling to start people with COPD on beta-blockers because older beta-blockers had lung side effects. However, new evidence shows that newer beta-blockers targeting the heart, e.g. bisoprolol, are safe in people with COPD. In addition, there is now also evidence that people with COPD who take beta-blockers are less likely to have flare ups even if they have no heart problems. This unexpected benefit of beta-blockers has not been tested in patients with COPD who are not known to need beta-blockers.

The aim of this large randomised controlled trial is to see if starting people with COPD on the beta-blocker bisoprolol reduces flare ups. In this five-year study we will see if adding bisoprolol to usual COPD medicines reduces the number of COPD flare ups and makes patients feel better. Over a three year period we will recruit 1,574 patients with COPD who have recently had flare ups (at least two within a year). Potential participants will be identified from general practice and hospital chest clinics. We will recruit in 160 UK centres. Over 50% of subjects will be recruited from General Practice. Half of the 1,574 subjects will take a bisoprolol tablet for a year. The other half will take an identical dummy pill. To work out the best dose of bisoprolol (and dummy pill) for each patient we will slowly build up the dose over 4 weeks whilst checking pulse and blood pressure. This is known to be safe.

In response to COVID-19 we have made the study as COVID-19 safe as possible. When we started the study, people taking part were seen 7 times: baseline, 1, 2, 3, 4, 26 and 52 weeks. To try and minimise the chances of catching COVID-19, we have reduced the number of face-to-face appointments and replaced them with a telephone or video call.

Some people will still have a face-to-face first baseline visit, and the rest of the assessments will be by telephone or video call. Other people may have all of their assessments by telephone or video call including the first baseline visit. The dose of bisoprolol (and the dummy pill) will be increased at the baseline, 1, 2 and 3 week assessments. At baseline, 26 and 52 weeks we will ask the participants to count the number of flare ups, hospital episodes, ability to get on with their lives, side effects and heart problems. When we started the study, people did standard breathing tests to measure how well the lungs were working. It is not possible to do breathing tests during COVID-19, so we will not do these while the pandemic is ongoing to reduce the risk of infection. We will also count how many times patients have seen a doctor/nurse to see if bisoprolol reduces NHS costs. We will ask GPs for relevant information about exacerbations on those who leave the study early.

At the end of the study we will compare the number of flare ups in the group taking bisoprolol against the group taking the dummy pill. If we show that bisoprolol reduces the risk of COPD flare ups then it will be possible to include the finding in advice given to doctors on how to treat people with COPD. Bisoprolol is cheap (4p/day) and if it works it will improve the lives of people with COPD and reduce the costs of COPD to the NHS.

## Scientific summary

***Rationale:*** Chronic Obstructive Pulmonary Disease (COPD) is a progressive lung disease characterised by progressive airflow limitation. The prevalence of diagnosed COPD in the UK has increased from about 991,000 in 2004 to 1.2 million in 2012. COPD is the fifth leading cause of death in the UK and costs the NHS approximately £1 billion annually. Exacerbations of COPD account for 60% of NHS COPD costs and are associated with accelerated rate of lung function decline, reduced physical activity, reduced quality of life, increased mortality and increased risk of comorbidities such as acute myocardial infarction and stroke. In the UK emergency hospital admissions for exacerbations of COPD have steadily increased as a percentage of all admissions from 0.5% in 1991 to 1% in 2000 to 1.5% in 2008/9.

Beta-blockers are a well-established therapy in cardiovascular medicine because of beneficial effects on morbidity and mortality in people with ischaemic heart disease. There is now a substantial body of evidence from observational studies demonstrating that beta-blocker use in people with COPD is associated with a reduced risk of COPD exacerbations, moreover the beneficial association with exacerbations extends to people with COPD without cardiovascular disease and appears to be restricted to beta-blockers and not evident with other cardioactive drugs e.g. ACE inhibitors.

This study will investigate the effectiveness of repurposing the beta-blocker bisoprolol from a drug with recognised cardiovascular benefits to a drug that also has benefits in preventing COPD exacerbations.

As a consequence of the COVID-19 pandemic, changes have been implemented to minimise risk to participants, to conform with COVID-19 measures and to mitigate the impact on the study.

***Objectives:*** To determine the clinical effectiveness and cost-effectiveness of adding bisoprolol (maximal dose 5mg once a day, or maximum tolerated dose) to usual COPD therapies in patients with COPD at high risk of exacerbation because of a history of at least two COPD exacerbations in a previous year:

The primary clinical outcome is the number of participant reported COPD exacerbations necessitating a change in management (minimum change treatment with antibiotics and/or oral corticosteroids) during the one year treatment period.

The primary economic outcome is the cost-per-QALY gained during the one year treatment period.

***Method:*** A pragmatic randomised, double-blind, placebo-controlled, multicentre clinical trial.

***Setting:*** Primary and Secondary care; General Practice and NHS hospitals.

***Target population:*** 1,574 patients with diagnosed COPD (post bronchodilator $FEV_1$<80% predicted, $FEV_1$/FVC<0.7), and with a history of ≥2 COPD exacerbations in the previous year requiring treatment with oral corticosteroids and/or antibiotics OR a history of at least two exacerbations within 12 months of each other requiring treatment with antibiotics and/or oral corticosteroid since March 2019.

***Main exclusion criteria:*** patients currently using beta-blockers, a current sole respiratory diagnosis of asthma, a diagnosis of asthma before the age of 40 years, diagnosed heart failure and within the previous 12 months myocardial infarction/acute coronary syndrome, drugs that interact with beta-blockers.

**Randomisation:** When clinically stable subjects will be assessed, recruited and randomised with equal probability to intervention or control group.

***Intervention:*** Intervention and control groups will receive usual NHS care. The intervention will be either bisoprolol (1.25mg tablets) or an identical placebo. The starting dose of bisoprolol will be one 1.25mg tablet a day, this dose will be increased approximately weekly 1.25mg→2.5mg→3.75mg→5mg resulting in final doses of 1.25mg od (1 tablet), 2.50 mg od (2 tablets), 3.75mg od (3 tablets), or 5mg od (4 tablets) depending on tolerance to bisoprolol up-dosing.

The dose of placebo will be one tablet a day. The dose will be increased approximately weekly 1 tablet→2 tablets→3 tablets→4 tablets resulting in final doses of 1, 2, 3 or 4 tablets a day depending on tolerance to up-dosing.

During dose titration, decisions to increase, maintain or reduce the study drug dose will be based on monitoring heart rate, blood pressure, lung function (during the COVID-19 pandemic, changes in patient-reported breathlessness will replace measurement of lung function) and clinical status (symptoms, including respiratory symptoms, fatigue).

Participants will remain on final titrated dose for the remaining 48 weeks.

Following completion of the 12 month dosing period, participants will be weaned off study drug over following 3 weeks (3-2-1 tablet od).

***Assessment:*** Participants will be reviewed on 7 occasions during the 1 year study: after recruitment/ randomisation participants will be reviewed at 1, 2, 3, 4, 26 and 52 weeks. During COVID-19, the recruitment/baseline visit may be face-to-face or carried out by telephone/video call; all subsequent assessments will be by telephone or video call.

The assessments at 1, 2, 3, 4 weeks are necessary for guideline compliant beta-blocker dose titration.

The assessments at recruitment/randomisation and at 26 and 52 weeks will be for the collection of the following outcome data: history of exacerbations, health care utilisation, disease-related quality of life status (COPD assessment test [CAT]), breathlessness using the Baseline and Transition Dyspnoea Indices (BDI and TDI), health related quality of life (EQ-5D-5L), lung function (FEV1, FVC – not done during COVID-19 pandemic), adverse reactions and serious adverse events. The Hull Airways Reflux Questionnaire (HARQ) will be used at some sites to collect data on respiratory and gastrointestinal symptoms.

***Clinical relevance:*** Bisoprolol is a cheap (4p/day) readily available generic drug and most clinicians are familiar with its use in heart disease in primary and secondary care. If shown to be effective it will improve the quality of life of COPD patients and reduce the burden of COPD on the NHS.

## iv. FUNDING AND SUPPORT IN KIND

| FUNDER(S) | FINANCIAL AND NON FINANCIALSUPPORT GIVEN |
|---|---|
| National Institute for Health Research, Health Technology Assessment | £2,467,469.00 |

## v. ROLE OF TRIAL SPONSOR AND FUNDER

The Sponsor (co-sponsor) has responsibility for the initiation and management of the trial as defined by the UK Policy Framework for Health and Social Care Research v3.3 07/11/17. This is further defined within a co-sponsorship agreement outlining the roles and responsibilities of the parties involved in the research. Specific responsibilities delegated to another party are formally agreed and documented by the Sponsor.

The funder has oversight of the study through regular reports from the trial office.  The funder appoints the independent members of the Data Monitoring and Trial Steering Committees and receives minutes from these. The funder is made aware of all outputs from the study but does not have a role in the decision to publish results from the study.  In any publications, the funder is acknowledged, and appropriate disclaimer used to indicate that the views expressed are those of the author(s) and not necessarily those of the NHS, the NIHR or the Department of Health.

## vi. ROLES AND RESPONSIBILITIES OF TRIAL MANAGEMENT COMMITEES/GROUPS & INDIVIDUALS

The trial will be coordinated by a Project Management Group, consisting of the grantholders (Chief Investigator and other grant holders as appropriate), the Trial Manager and other senior members of the Trials Unit.

A Trial Manager will oversee the study and will be accountable to the Chief Investigator.  The Trial Manager will be based in the Central Trial Office which will provide support to each site.

A Trial Steering Committee (TSC), with independent members, will be established to oversee the conduct and progress of the trial. The membership and terms of reference of the Trial Steering Committee will be filed in the Trial Master File.

An independent Data Monitoring Committee (DMC) will be established to oversee the safety of subjects in the trial. The membership and terms of reference of the DMC will be filed in the Trial Master File.

## vii. PROTOCOL CONTRIBUTORS

All those listed above as key contributors to the protocol have actively contributed to the protocol in accordance with their individual skills and expertise.

## viii. KEY WORDS:

COPD, bisoprolol, exacerbations, randomised trial, placebo controlled, pragmatic

## ix. TRIAL FLOW CHART

Figure 1 – trial summary

Recruitment/baseline assessment – assessed for eligibility based on inclusion and exclusion criteria
Informed consent taken
Assessment: drug history, smoking history, spirometry[1], CATest, BDI dyspnoea score, EQ-5D-5L, health care utilisation.
Pulse, blood pressure (BP).

↓

Centralised randomisation (n=1,574)
University of Aberdeen, Centre for Healthcare Randomised Trials, web/telephone randomisation service

↓

| Intervention arm | Control arm |
|---|---|
| **Intervention arm: n=787**<br>Bisoprolol 1.25mg once daily (1 x 1.25mg tab). | **Control arm: n=787**<br>Placebo 1 tab once daily |
| ↓ | ↓ |
| **1 week**<br>Pulse, BP, FEV1[2], symptoms, adverse reactions<br>↑Bisoprolol 2.5mg once daily (2 x 1.25mg tabs)[3] | **1 week**<br>Pulse, BP, FEV$_1$[2], symptoms, adverse reactions<br>↑ Placebo 2 tabs once daily[3] |
| ↓ | ↓ |
| **2 weeks**<br>Pulse, BP, FEV$_1$[2], symptoms, adverse reactions<br>↑Bisoprolol 3.75mg once daily (3 x 1.25 mg tabs)[3] | **2 weeks**<br>Pulse, BP, FEV$_1$[2], symptoms, adverse reactions<br>↑ Placebo 3 tabs once daily[3] |
| ↓ | ↓ |
| **3 weeks**<br>Pulse, BP, FEV$_1$[2], symptoms, adverse reactions<br>↑Bisoprolol 5mg once daily (4 x 1.25mg tabs)[3] | **3 weeks**<br>Pulse, BP, FEV[2]$_1$, symptoms, adverse reactions<br>↑ Placebo 4 tabs once daily[3] |
| ↓ | ↓ |
| **4 weeks**<br>Pulse, BP, FEV$_1$[2], symptoms, adverse reactions[3] | **4 weeks**<br>Pulse, BP, FEV1[2], symptoms, adverse reactions[3] |

↓

**6 month follow-up:** exacerbations, hospital admissions, health care utilisation, drug history, spirometry[4], disease specific health status (CATest), TDI dyspnoea, generic health related quality of life (EQ-5D-5L), adverse reactions, serious adverse events, weight, Pulse, BP.

↓

**12 month follow-up:** exacerbations, hospital admissions, health care utilisation, drug history, spirometry[4], disease specific health status (CATest), TDI dyspnoea, generic health related quality of life (EQ-5D-5L), adverse reactions, serious adverse events. Pulse, BP.

*Advise to reduce tablets by one tab a day per week to zero*

↓

**4 week follow-up telephone call:** Ensure that medication has been stopped and no adverse events.

[1] During the COVID-19 pandemic, historical spirometry results will be used.
[2] During the COVID-19 pandemic, the titration algorithm will consider patient reported breathlessness and not FEV$_1$.
[3] For further details regarding titration, including cases where there may be down-titration, please see section 8.7 and figure 3.
[4] During the COVID-19 pandemic, 6 and 12 month follow-up will be remote assessments and not include spirometry.

# 1 BACKGROUND

COPD is a common lung disease [1,2]. In the UK the prevalence of diagnosed COPD has increased from about 991,000 in 2004 to 1.2 million in 2012 [3], it is the fifth leading cause of death in the UK, accounting for about 5% of all deaths (~30,000 deaths in 2014). The progressive airflow limitation of COPD is associated with increasing disability, work absence, long-term morbidity, common physical and psychological co-morbidities, and premature mortality. People with COPD are more likely to have associated comorbidities [4], including ischaemic heart disease [5], hypertension [6], heart failure, diabetes [7], metabolic syndrome, osteoporosis [8], depression [9] and lung cancer [10], which increase morbidity and complicate its management [11]. Unrecognised heart failure has been reported in up to 20% of COPD patients [12,13].

Acute deteriorations in symptoms known as exacerbations are an important clinical feature of COPD. These are usually precipitated by viral/bacterial infection and/or air pollution and are characterised by increasing breathlessness, and/or cough, sputum expectoration and malaise. Exacerbations are associated with accelerated rate of lung function decline [14], reduced physical activity [15], reduced quality of life [16], increased mortality [17] and increased risk of comorbidities such as acute myocardial infarction and stroke [18]. It has been estimated that exacerbations account for about 60% of the £1billion NHS expenditure on COPD [19]. Emergency hospital admissions for exacerbations of COPD have steadily increased as a percentage of all admissions from 0.5% in 1991 to 1% in 2000 to 1.5% in 2008/9 [20]. In 2008/9, COPD exacerbations resulted in 164,000 hospital admissions in the UK with an average length of stay of 7.8 days, accounting for 1.3 million bed days. COPD is the second leading cause of emergency admission to hospital in the UK and is one of the most costly inpatient conditions treated by the NHS [1,2]. Over 30% of patients admitted to hospital with an exacerbation of COPD are readmitted within 30 days and an average of 12% of COPD patients die in the year following admission to hospital [17].

Despite advances in management there is still an unmet need for improved pharmacological treatment of COPD particularly the prevention of exacerbations.

# 2 RATIONALE

**Rationale for investigating bisoprolol**

It is well established that beta-blockers reduce morbidity and mortality in people with ischaemic heart disease. However, there is now a substantial body of evidence from observational studies that beta-blocker use in people with COPD is associated with a reduced risk of COPD exacerbations, moreover the beneficial association with exacerbations extends to people with COPD without cardiovascular disease and appears to be class specific [21-27].

A systematic review of observational studies of beta-blockers in people with COPD identified 15 studies with a total 121,956 COPD patients followed up for between 1 and 7 years. Meta-analysis demonstrated that beta-blocker use in COPD patients was associated with 28% (95% CI 17-37) reduced mortality, and 37% (95% CI 29-43) reduced exacerbations [21]. Subsequent to this review Bhatt et al reported that in the observational multicentre COPDGene cohort of 3464 people with COPD followed up for a median of 2.1 years, beta-blocker use was associated with a reduced risk of COPD exacerbation (incidence risk ratio IRR 0.71, 95% CI 0.58-0.87) [23]. Use of CT imaging to quantify coronary artery calcification enabled investigators to identify COPD patients with and without coronary artery disease. Beta-blocker use was associated with a reduced risk of COPD exacerbation in COPD patients with CT evidence of coronary artery disease (IRR 0.67, 95% CI 0.48-0.93) and in the 82% of their COPD patients without coronary artery disease on CT imaging (IRR 0.76, 95% CI 0.58-0.99). This study also noted that the beneficial effect of beta-blockers was evident in patients requiring long term oxygen therapy and was class specific, with the use of other cardio-active drugs used to treat heart disease (e.g. calcium channel blockers, ACE inhibitors, AR blockers) having no demonstrable effect on the risk of COPD exacerbation.

**Possible mechanisms by which beta-blockers could reduce COPD exacerbations**

The mechanisms by which beta-blockers may reduce exacerbations remain uncertain though several explanations are biologically plausible.

1. Beta-blockers reduce the activity of the sympathetic nervous system that is known to be markedly increased in COPD and adversely associated with morbidity, mortality and inflammatory indices [28,29].
2. Beta-blockers have some direct anti-inflammatory properties potentially relevant to COPD exacerbations [30,31].
3. COPD and cardiovascular disease share common risk factors and are frequently present together [32]. It is plausible that some of the episodes conventionally diagnosed as acute exacerbations of COPD are in fact cardiac events such as subclinical coronary ischemia for which beta-blockers have proven benefits [18].

Irrespective of the mechanism, demonstrating that beta-blockers reduce the risk of COPD exacerbation will have major beneficial effects for people with COPD, their families and the NHS.

**Hypothesis**

The hypothesis being tested is that the repurposing of a well-known and inexpensive drug bisoprolol added to usual COPD treatment reduces the risk of COPD exacerbation requiring treatment with antibiotics and/or oral corticosteroids during the year of treatment, delivers quality of life improvements, is safe and cost-effective.

**Drug and dosing**

The interventions will be the cardio-selective beta-blocker bisoprolol (1.25mg tablets) and identical placebo. Bisoprolol will be used because it is licensed for use in heart failure in the UK and is a low cost intervention readily adoptable into routine practice/COPD guidelines. Bisoprolol has a high β1:β2 receptor selectivity ratio of 14:1, which is higher than either atenolol (5:1) or metoprolol (2:1) [33].

To ensure patient safety bisoprolol will be started at the lowest possible dose of 1.25mg once a day and up-titrated at weekly intervals up to the maximum tolerated dose or 5mg a day, whichever is the lower. The dose titration schedule is a conservative interpretation of the advice provided in Heart Failure guidelines, the SmPC for bisoprolol and NHS Grampian heart failure guidelines designed for use by appropriately trained nurses in primary care settings [34-38].

The starting dose for bisoprolol is one 1.25mg tablet taken orally daily and participants will undergo a weekly dose titration regime (i.e. weekly increments of 1.25mg→2.5mg→3.75mg→5mg) as outlined below which will result in final doses of 1.25mg once daily (od) (1 tab), 2.50 mg od (2 tabs), 3.75mg od (3 tabs), or 5mg od (4 tabs) depending on tolerance to bisoprolol up dosing. Participants allocated to placebo will undergo an identical dose-titration regime, with a final dose of 1, 2, 3 or 4 tablets a day.

Following completion of the 12 month dosing period, participants will be weaned off study drug over the following 3 weeks (3-2-1 tablet od) in order to avoid possible rebound myocardial ischemia.

The maximum bisoprolol dose of 5mg, whilst less than the maximal dose (10mg) usually advocated in heart failure [34,36,38], maximises β1-blockade whilst minimising any potential adverse broncho-constricting effects on the airways. It is our experience that 5mg a day is the most commonly tolerated dose in real life practice in elderly patients with COPD. Although bisoprolol has a high β1:β2 receptor selectivity ratio of 14:1 [33] the dose response characteristics of β1 and β2-blockade differ. β1-blockade with bisoprolol is maximal at 5mg od, whereas potentially adverse β2-blockade increases markedly above 5mg od [39].

## 2.1 Assessment and management of risk

**Cardioselective beta-blockers are safe to use in people with COPD**

The beta-adrenergic system contains ß1 and ß2 adrenoreceptors, ß1 adrenoreceptors are found only in the heart, while ß2 adrenoreceptors are more ubiquitous, being found in the heart and lungs. Bronchodilating ß2-agonists are the mainstay of COPD and asthma treatment. Beta-blockers that antagonise the effects of ß2-agonists could have adverse respiratory effects, indeed for asthma, a condition with reversible airflow limitation, beta-blockers are absolutely contraindicated [40].

ß1-selective blockers such as bisoprolol have 14 times more affinity for ß1 adrenoreceptors than for ß2 adrenoreceptors [33] and should not cause bronchoconstriction. Clinical evidence is supportive of bisoprolol being safe in COPD and ß1-selective blocker use in COPD patients with heart failure is a guideline recommendation [34,36].

A systematic review of RCTs studying effects of ß1-selective blockers on lung function ($FEV_1$) and respiratory symptoms in people with COPD using ß2-agonists identified 11 single dose and 11 repeated dose trials [41]. Administration of single doses of ß1-selective blockers to 131 patients was not associated with a change in $FEV_1$ compared to placebo or to baseline controls, (mean difference (MD) -2.08%, 95% CI -5.25 to 1.09), in absolute terms the MD was 33ml of $FEV_1$. Administration of single doses of ß1-selective blockers was not associated with an increase in respiratory symptoms or change in the response to inhaled ß2-agonist after treatment and after placebo (MD -1.21%; 95% CI -10.97 to 8.56). Administration of repeated doses of ß1-selective blockers to 185 patients for a total of 1,436 patient weeks (range 2 days to 16 weeks) was not associated with a change in $FEV_1$ (MD -2.73%; 95% CI -6.03 to 0.57), in absolute terms the MD was 49ml of $FEV_1$. Repeated dosing was not associated with an increase in respiratory symptoms or a change in the response to inhaled ß2-agonist after treatment and after placebo (MD -0.70%; 95% CI -5.02 to 3.63). Limiting the meta-analyses to patients with severe COPD revealed that use of ß1-selective blockers was not associated with a change in $FEV_1$, respiratory symptoms or response to inhaled ß2-agonist treatment.

Most recently a randomized cross-over study of 18 patients with moderate to severe COPD comparing bisoprolol (5mg once daily) and carvedilol (12.5mg twice daily) each for 6 weeks in conjunction with sequential inhaled step down therapy has been completed. This trial demonstrates that addition of bisoprolol to COPD therapy combinations of inhaled corticosteroids (ICS), long acting ß2 agonists (LABA) and long acting antimuscrinic agents (LAMA), e.g. ICS/LABA, ICS/LABA/LAMA significantly reduces heart rate but there were no significant effects on parameters of lung function (including $FEV_1$, FVC), blood pressure, quality of life or breathlessness [42].

**Potential risks and benefits**

Bisoprolol is an inexpensive generic drug licensed for use in heart disease in the UK. It is familiar to most practising clinicians involved in primary and secondary care in the UK in the context of heart disease; its pharmacokinetics and side effect profile are well characterised. Although current Heart Failure Guidelines advocate the use of bisoprolol for people with heart failure even in the presence of COPD, the current study will be using bisoprolol off label outwith its licensed indication.

The main anticipated risk to participants is that they suffer an undesirable side effect (e.g. dizziness, fatigue, headache). However, by using the 'start low and slow' dose titration schedule recommended in heart failure guidelines the risk of this will be minimised, moreover the maximal dose of bisoprolol chosen (5mg) is less than the maximal dose recommended for heart failure (10mg). This dose has been chosen based on clinical experience of its use in people with COPD and consideration of pharmacodynamics [39]: 5mg represents a balance between beneficial β1 blockade and adverse β2 blockade. As discussed above the perceived adverse effect of beta-blockers on the airways of people with COPD is unfounded. People with asthma will be excluded from the study.

It is anticipated that trial participants allocated to bisoprolol will benefit because of reduced rate of exacerbations of COPD with consequent improvement in health status, participants allocated to placebo will have an unchanged rate of exacerbation.

The potential benefits to society arise from the widespread use of a cheap familiar generic drug (bisoprolol) in the management of COPD. Exacerbations of COPD are associated with an adverse prognosis, increased mortality, and a reduced quality of life, moreover COPD exacerbations are the most costly aspect of COPD to the NHS, accounting for 60% of total NHS COPD direct costs and significant winter bed pressures disrupting other NHS users.

**COVID-19**

During the conduct of this trial the COVID-19 pandemic and new risks emerged. The participants in this trial are considered vulnerable to COVID-19 because of they all have COPD and at high risk of exacerbation. Furthermore, spirometry is classified as an aerosol generating procedure and effectively not possible to perform easily and safely in the context of a pragmatic trial. To mitigate these risks the number of face to face assessments have been reduced to a maximum of one, national PPE/social distancing/hand hygiene guidelines will be followed and spirometry has been replaced.

This trial is categorised as:

- Type B = Somewhat higher than the risk of standard medical care

## 3 OBJECTIVES AND OUTCOME MEASURES/ENDPOINTS

### 3.1 Primary objective

The primary objective of this trial is to determine the clinical (in terms of number of exacerbations requiring change in management) and cost-effectiveness of adding bisoprolol (maximal dose 5mg once a day, or maximum tolerated dose) to usual COPD therapies in patients with COPD at high risk of exacerbation because of a history of at least two COPD exacerbations in a previous year.

### 3.2 Secondary objectives

The secondary objectives are to compare the following outcomes between participants treated with bisoprolol, and those treated with placebo:

- Hospital admissions with a primary diagnosis of COPD exacerbation.
- Total number of emergency hospital admissions.
- Total number of major adverse cardiovascular events (MACE).
- Lung function (during the COVID-19 pandemic, lung function cannot be assessed in participants).
- Changes in breathlessness during treatment.
- All-cause, respiratory and cardiac mortality.
- Drug reactions and serious adverse events.
- Health related quality of life.
- Disease specific health status.
- Health care utilisation.
- Incremental cost-per-exacerbation avoided.
- Costs to the NHS and patients and lifetime cost-effectiveness based on extrapolation modelling.
- Modelled lifetime incremental cost per Quality Adjusted Life Year.
- Treatment effects in participants with and without clinically diagnosed heart disease.

### 3.3 Primary endpoint/outcome

The primary outcome measure will be the total number of exacerbations of COPD necessitating changes in management (minimum management change - use of oral corticosteroids and/or antibiotics) during the one year treatment period, as reported by the participant.

The primary economic outcome measure will be cost-per-QALY gained during the one year treatment period.

### 3.4 Secondary endpoints/outcomes

- Total number of COPD exacerbations requiring hospital admission*.
- Time to first exacerbation of COPD.
- Total number of emergency hospital admissions (all causes)*.
- Total number of major adverse cardiovascular events (MACE) (defined by cardiovascular death, hospitalisation for myocardial infarction, heart failure, or stroke), percutaneous coronary intervention or coronary artery bypass grafting*.
- Lung function ($FEV_1$, FVC) post bronchodilator using spirometry performed to ATS/ERS standards (during the COVID-19 pandemic, lung function cannot be assessed safely and so this outcome will not be available on all participants).
- Breathlessness using Baseline and Transition Dyspnoea Indices (BDI & TDI).
- All-cause, respiratory and cardiac mortality*.
- Serious adverse events, adverse reactions.
- Health related quality of life using EuroQoL 5D (EQ-5D-5L) Index.
- Disease specific health status using the COPD Assessment Test (CAT).
- Utilisation of primary or secondary health care for respiratory events*.
- Change in disease associated symptoms using the Hull Airways Reflux Questionnaire (HARQ) [*this will only be done at some recruitment sites*].
- Modelled lifetime incremental cost per Quality Adjusted Life Year.

*The time period will be the year of treatment, i.e. number events per year of treatment.

## 4 TRIAL DESIGN

The study is a pragmatic double-blind randomised, placebo-controlled, UK multicentre clinical trial comparing the addition of bisoprolol or placebo for one year to current COPD therapy, in patients with COPD who have had two or more exacerbations of COPD in a previous year treated with oral corticosteroids and/or antibiotics. Figure 2 provides a schematic representation of study design.

*Figure 2: Study design*

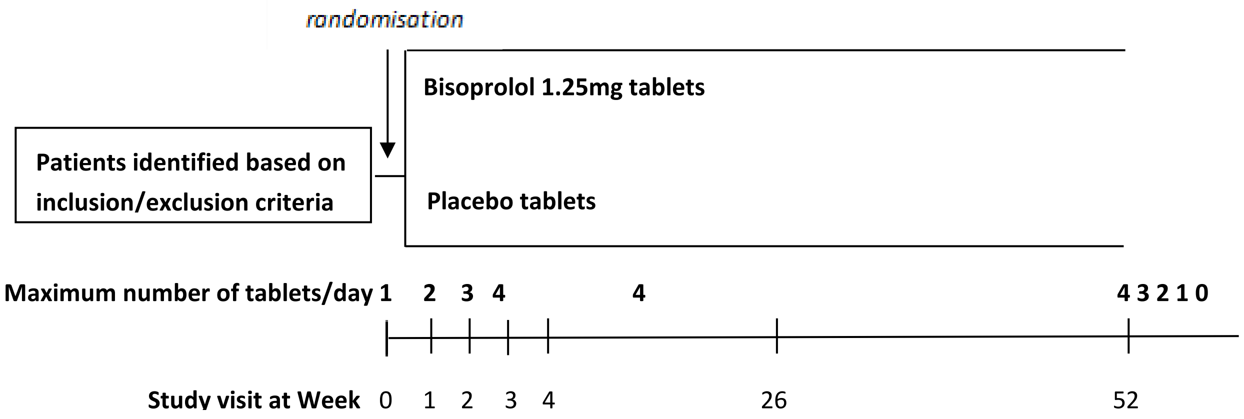


Face-to-face study assessments will be carried out in all subjects at recruitment/baseline, 6, and 12 months, dose titration visits will take place at 1, 2, 3 and 4 weeks as shown in Figure 2. Where face-to-face study assessments (for example, during COVID-19) are not possible, these will be carried out as telephone or video calls.

# 5 TRIAL SETTING

Potential participants will be recruited from both primary and secondary care across the UK.

# 6 PARTICIPANT ELIGIBILITY CRITERIA

## 6.1 Inclusion criteria

Eligibility for inclusion in the trial will be confirmed by a medically qualified person and recorded in the medical notes.

Patients will be enrolled if they meet all of the following criteria:

- Aged ≥ 40 years.
- A smoking history of at least 10 pack years ([average number of cigarettes/day x years smoked]/20).
- An established predominant diagnosis of COPD (NICE Guideline definition: post bronchodilator[1] $FEV_1$<80% predicted, $FEV_1$/FVC<0.7)[2] receiving treatment as per local guidelines' [2][3,4]. Patients with asthma COPD overlap syndrome (ACOS) will also be eligible.
- A history of at least two exacerbations requiring treatment with antibiotics and/or oral corticosteroid use in the previous year, based on patient report OR a history of at least two exacerbations within 12 months of each other requiring treatment with antibiotics and/or oral corticosteroid since March 2019[5].
- Clinically stable with no COPD exacerbation for at least 4 weeks.
- Able to swallow study medication.
- Able and willing to give informed consent to participate.
- Able and willing to participate in the study procedures, complete study questionnaire.
- Able and willing to undergo spirometric assessment, able to perform an $FEV_1$ manoeuvre as a minimum[6]. During the COVID-19 pandemic, measurement of $FEV_1$ is not required as part of the protocol, and therefore this inclusion criteria does not need to be met.

[1] "post-bronchodilator" it is anticipated that participants will have used their own LABA within 8 hours or their own SABA within 2 hours. If the patient has not taken their LABA within 8 hours or their SABA within 2 hours, lung function should be measured 15 minutes after administration of the participant's own SABA. If the patient is not bronchodilated and does not have their own SABA with them, they should not be given any clinical supply of SABA. Spirometry can be done without bronchodilation.

[2.] During the COVID-19 pandemic, we will use historic spirometry to confirm the diagnosis of COPD. Spirometry is an aerosol generating procedure, so should not be done during the COVID-19 pandemic for research purposes within BICS. The inclusion criteria $FEV_1$<80% predicted, $FEV_1$/FVC<0.7) should be assessed based on any historical spirometry documented in the medical notes.

[3] For those participants who do not fulfil the $FEV_1$/FVC <0.7 at the time of testing despite a diagnosis of COPD, it is acceptable to conduct a slow vital capacity manoeuvre and to accept $FEV_1$/SVC <0.7. If this criterion is not met historical evidence of, $FEV_1$/FVC<0.7 will be acceptable (however FEV1<80% predicted needs to be demonstrated at baseline – but see note 2 above, this is not a requirement during the COVID-19 pandemic).

[4] Spirometry equipment used should be maintained and serviced in line with manufacturer's guidelines.

[5] People with COPD were advised to shield in March 2020 due to COVID 19. These shielding arrangements remain in place to a greater or lesser degree, and many people are still partially shielding. Anecdotal evidence suggests that shielding reduced the number of exacerbations experienced by people with COPD. The ECLIPSE study has demonstrated that the frequent exacerbator phenotype is stable for at least 3 years[57]. The modification of the inclusion criteria to include ≥2 exacerbations in any 12 month period since March 2019 enables the identification of the people at high risk of exacerbation during the time of reduced exacerbation risk during lockdown by combining exacerbations before and after the lockdown.

[6] There are a number of surgical procedures and other conditions after which spirometry is not recommended for a period of time. The table below is a pragmatic conservative adaptation of the recommendations of Cooper [44]. Other clinical or personal circumstances may preclude spirometry in individual cases. Such cases should be discussed with the study team. In cases where spirometry is contraindicated, entry into the trial should be delayed until the participant is able to perform spirometry.

| Surgical procedure/condition | Notes about spirometry |
|---|---|
| Thoracic/abdominal surgery | 3 months post surgery |
| Brain, eye, ear, ENT surgery | 3 months post surgery |
| Pneumothorax | 3 months post resolution |
| Myocardial infarction | 3 months |
| Ascending aortic aneurysm | 3 months post repair |
| Haemoptysis | 1 month post free of haemoptysis |
| Pulmonary embolism | Safe if on anticoagulation |
| Angina | Safe if stable |
| Severe hypertension (systolic >200 mm Hg, diastolic >120 mm Hg): | Measure blood pressure if suspected |

## 6.2 Exclusion criteria

- A current sole respiratory diagnosis of asthma.
- Any diagnosis of asthma before the age of 40 years.
- A predominant respiratory disease other than COPD.
- Any significant disease/disorder which, in the investigator's opinion, either puts the patient at risk because of study participation or may influence the results of the study or the patient's ability to participate in the study.
- Previous allocation of a randomisation code in the study or current participation in the active intervention phase of another interventional study (CTIMP or non-CTIMP). [*as reported by the participant or documented in the medical notes*]
- Already taking beta-blocker.
- Known or suspected hypersensitivity to beta-blocker.
- For women, current pregnancy or breast-feeding, or planned pregnancy during the study.
- Unable to perform spirometry ($FEV_1$ manoeuvre).
- Current resting (5 minutes sitting) heart rate <60 bpm.
- Current resting (5 minutes sitting) systolic blood pressure <100mmHg.
- $2^{nd}$, $3^{rd}$ degree heart block (unless pacemaker *in situ*).
- Conditions for which beta-blocker use is a guideline recommendation, i.e. heart failure, or within the last year: myocardial infarction, acute coronary syndrome.
- Current tachyarrythmia or bradyarrhythmia (including sick sinus syndrome, sinoatrial block) requiring treatment.
- Current treatment with interacting drugs:
    - heart rate limiting drug such as calcium channel blockers (diltiazem, verapamil), ivabradine,
    - class-I antiarrhythmic drugs (e.g. quinidine, disopyramide; lidocaine, phenytoin; flecainide, propafenone),
    - centrally-acting antihypertensive drugs (e.g. clonidine methyldopa, moxonidine, rilmenidine) [35].
- Severe peripheral arterial occlusive disease, severe forms of Raynauds syndrome.
- Conditions that are known to be triggered by beta-blockers or beta-blocker withdrawal including myasthenia gravis, periodic hypokalaemic paralysis, pheochromocytoma, thyrotoxicosis and psoriasis/history of psoriasis.
- People without capacity.

Long term oxygen therapy is not an exclusion criterion.

# 7 TRIAL PROCEDURES

## 7.1 Participant identification and Recruitment

Potential participants will be recruited from both primary and secondary care across the UK; however it is envisaged that the majority of participants (>50%) will be recruited within primary care. Recruitment strategies will differ between centres depending on local geographic and NHS organisational factors.

***Primary care***

In England recruitment from General Practices will be conducted in conjunction with the NIHR Clinical Research Network (CRN) at both the national and local level.

For General Practices acting as Participant Identification Centres (PICs), the local CRN/collaborating recruitment site/Trial Office will liaise directly with practice managers/GPs who will perform a database search (based on search criteria including one exacerbation treated with oral corticosteroids in previous year, interacting medications) to identify potential participants. Potentially suitable participants will be sent an invitation letter on practice headed paper and a short patient information leaflet (PIL). The letter will provide a range of methods for interested potential participants to contact the local trial team (telephone, text, e-mail, reply paid envelope) for more information (including a more comprehensive PIL) and to arrange a recruitment assessment should the potential participant be agreeable. General Practices can act as PICs for secondary care sites or for primary care sites. Where possible, GP records can be screened on two occasions at least a year apart. PIC activity (with face-to-face recruitment) will only be used within the study at times when there are no local travel restrictions in place. At other times, PIC activity will only be used where recruitment and consent is done without a face-to-face visit (see section 7.2 (B)).

For General Practices acting as independent study sites, the trial office/local CRN/ will liaise directly with GP practice managers/GPs who will perform a database search (based on search criteria described above) to identify potential participants. Potentially suitable participants will be sent an invitation letter on practice headed paper and a PIL informing them of the trial aims and level of participation required. The letter will provide a range of methods for interested potential participants to contact the local general practice based trial team (telephone, text, e-mail, reply paid envelope) for more information and to arrange a recruitment assessment should the potential participant be agreeable. Where possible, GP records can be re-screened.

In Scotland the Scottish Primary Care Research Network will mirror the role undertaken by the English CRN by identifying potential participants in primary care.

In some centres COPD Community Matrons and other Integrated/Intermediate Care services for patients with COPD are available. Potentially suitable participants will be sent an invitation letter on headed paper and a PIL informing them of the trial aims and level of participation required. The letter will provide a range of methods for interested potential participants to contact the local trial team (for example telephone, text, e-mail, reply paid envelope) for more information and to arrange a recruitment visit should the potential participant be agreeable.

Patients with COPD attending or who have attended Pulmonary Rehabilitation classes will be sent an invitation letter on headed paper and a PIL informing them of the trial aims and level of participation required. The letter will provide a range of methods for interested potential participants to contact the local trial team at a recruiting primary or secondary care site (for example telephone, text, e-mail, reply paid envelope) for more information and to arrange a recruitment assessment should the potential participant be agreeable.

Recruitment in primary care will be supplemented by posters located in General Practice waiting areas and Community Pharmacies.

Other potential avenues for identifying eligible patients include smoking cessation clinics, community spirometry clinics and other services provided in primary or secondary care for patients with COPD. As above, potentially eligible patients will be provided with an invitation letter on headed paper and PIL.

***Secondary care***

Potential participants will also be identified from patients who are attending (or who have previously attended) Respiratory Out-Patient appointments or in-patients at the hospitals of the individual recruiting centres. The first contact will be made by a member of the care team within the clinic; who will provide a brief overview of the study, a letter of invitation on headed paper and a PIL informing them of the trial aims and level of participation required. The letter will provide a range of methods for interested potential participants to contact the local trial team (for example telephone, text, e-mail, reply paid envelope) for more information and to arrange a recruitment assessment should the participant be agreeable.

If a trial centre has access to a Volunteer Database/Registry; participants (who meet the essential study eligibility criteria) will be identified and contacted via telephone/letter by a member of the research team. A letter of invitation on headed paper and PIL will be sent to the potential participant informing them of the trial aims and level of participation required. The letter will provide a range of methods for interested potential participants to contact the local trial team (for example telephone, text, e-mail, reply paid envelope) for more information and to arrange a recruitment assessment should the participant be agreeable.

In both primary and secondary care, where reply slips and reply paid envelopes are used for potential participants to get in touch to indicate their interest in taking part, the reply paid envelope may be returned to the trial office in Aberdeen, and the responses then forwarded to the appropriate recruitment site to contact the potential participant.

Again, in both primary and secondary care, docmail (http://www.docmail.co.uk/) can be used to mail invitation letters and study information to potential participants. Docmail is an online hybrid mail toolkit that it is used in the NHS to mail letters and other documents to patients.

### 7.1.2 Screening

Before entry into the trial, potential participants will be screened for the following:

- *Confirmation of COPD diagnosis.* This will require spirometric evidence (detailed section 6.1; see additional notes in this section where spirometry, as an aerosol generating procedure is not advised, with historical recordings being used instead)
- *Patient meets inclusion criteria detailed in section 6.2.*
- *Patient does not fulfil any exclusion criteria detailed in section 6.2*.

A study specific "trial inclusion form" will be used to document the eligibility of participants. This will be completed on paper, and will include space for a medical doctor to sign to confirm eligibility. The data will also be uploaded onto the study website.

Eligibility will be determined through discussion with the participant and information previously documented in medical records. If the participant requires to have spirometry or other examination to confirm eligibility, this should be done after informed consent.

**Ineligible and non-recruited participants**

Brief details of all patients screened at a recruitment appointment will be recorded (including, where known, age, sex and smoking status). If the patient was not eligible, the reason for this will be noted. If the patient was

eligible, but declined to take part, this will be noted. This information will be logged on the study specific "trial inclusion form", and data uploaded onto the study website.

Patients not recruited to the study will remain on their existing treatment.

#### 7.1.3 Payment

Reasonable travel expenses, in line with that awarded within the grant funding, for any visits additional to normal care will be recompensed.

During the COVID pandemic, we would advise use of taxi rather than public transport if the patient does not have their own transport available.

### 7.2 Consent

**A: in a face-to-face setting**

Potential participants who express interest in taking part in the study, and for whom a recruitment appointment is arranged will receive a copy of the long PIL prior to their recruitment appointment. As part of the informed consent process, potential participants should be made aware of all aspects of the study, including the potential risks and their responsibilities, particularly in terms of the safe return or disposal of unused study medication.

Informed consent to participate in the trial will be sought and received according to Good Clinical Practice (GCP) guidelines. Informed signed consent forms will be received from the participants by an appropriately trained individual who is listed on the delegation log. During the COVID-19 pandemic, to reduce footfall in hospitals and GP practices, a face-to-face recruitment visit can be done at home, with appropriate COVID-19 measures in place (PPE, social distancing).

**B: consent without a face-to-face visit**

Potential participants who express interest in taking part in the study will be contacted by telephone or video call (first consultation) and will have an initial discussion of the study. If they remain interested, a second telephone or video consultation will be arranged for them, after they have had opportunity to review the long PIL (which will be sent to them along with a copy of the consent form and pre-paid envelope). During this second telephone or video consultation, as part of the informed consent process, potential participants should be made aware of all aspects of the study, including the potential risks and their responsibilities, particularly in terms of the safe return or disposal of unused study medication. If the patient wishes to proceed with the study, informed consent to participate in the trial will be sought. The patient will be asked to initial the boxes on the consent form, then sign and date it, and return the top copy to the trial team. Once received by the trial team, the person who carried out the informed consent discussion should countersign the consent form.

Once the consent form has been received, a sphygmomanometer is dispatched the participant and a third telephone or video consultation is arranged. [Women of child-bearing potential will also be sent a pregnancy test, see section 9.6.1]. During this third consultation, the participant is told/shown how to use the sphygmomanometer and baseline data, including heart rate and blood pressure measurements are recorded.

Regardless of whether consent is sought in a face-to-face setting or without a face-to-face visit, there is no minimum time that potential participants should be given to decide whether or not to participate in the trial: potential participants will be given sufficient time, and as long as they themselves want, to accept or decline involvement and will be given opportunity to ask questions and to have these answered before giving consent. Before participants consent to the study, they should demonstrate an understanding of the potential risks of the study and their responsibilities in taking part. It will be explained that entry into the trial is entirely voluntary and that treatment and care will not be affected by their decision. It will also be explained that they can withdraw at any time. In the event of their withdrawal it will be explained that their data collected to date

cannot be erased and consent will be sought to use the data in the final analyses where appropriate. This can be documented on the original consent form if the participant is present, or on the eCRF if the agreement was given over the telephone. Participants who cannot give informed consent (e.g. due to their mental state) will not be eligible for participation. The consent will request consent to access to primary, secondary and intermediate/ integrated care records. Consent will also include agreement to pass the participant's name and address to the Central Trials Pharmacy that will dispense the study medication and to the courier who will deliver this.

Patients who are not able to read or write (but who have capacity) can agree to take part in the study. In such cases, the study team will provide them with written literature about the study and read and discuss this information with the potential participant. There should also be a discussion about the support networks that the patient has to facilitate their participation in the study. If the potential participant is fully informed and wishes to take part in the study, they will be asked to sign or make their mark on the consent form. Their agreement to take part in the study should be witnessed by someone independent from the research team.

Eligibility should be checked before consent is sought. However, if the participant requires to have spirometry, or other examination to confirm eligibility, consent to take part in the study should be sought prior to undertaking these study-specific procedures.

Following informed consent, if a participant loses capacity, the consent given when capable remains legally valid. In such circumstances, a decision needs to be made, in conjunction with the participant and any family or carers, in relation to ongoing participation in the study. If the decision is that the participant should cease study medication, then, where possible, the weaning process described in section 8.7.2 should be followed. If participants regain capacity, it will be established whether the participant consents to remain in the study.

A copy of the consent form should be forwarded to the trial office.

### 7.2.1 Additional consent provisions for collection and use of participant data and biological specimens in ancillary studies, if applicable

Potential participants will be asked to consent to receive invitations for ethically approved ancillary studies.

### 7.2.2 Participant safety

After randomisation, the participant's GP will be informed of their participation in the study. When informed of their patient's participation in the trial, GPs will be advised to manage their patient for exacerbations as per normal clinical practice and that they are to assume the participant is taking bisoprolol and the prescription of interacting drugs should be avoided.

During a face-to-face consent visit, participants will be given (and advised to carry) a credit card sized alert card giving brief information about the trial and advice for clinicians, contact details for the local investigator, and the contact details for emergency unblinding. If patients are recruited into the study without a face-to-face recruitment visit, the alert card will be sent to them after randomisation.

If the participant has nominated a "best contact", the best contact will be contacted by post to confirm their willingness to act as such. Best contact information is collected in case it is not possible to contact the participant themselves – for example if they have moved house or are in hospital.

## 7.3 Randomisation

A computerised randomisation system created by CHaRT will allocate participants with equal probability to intervention or control arms, stratified by centre (secondary care site, primary care area), and where participants were identified (primary care, secondary care). This randomisation application will be available 24 hours a day, 7 days a week as an interactive internet based application. Randomisation should only be carried

out once the eligibility has been confirmed by a medical doctor listed on the delegation log, and following consent. The randomisation application will be available to people listed on the delegation log with that responsibility and is not restricted to those who confirm eligibility or take consent.

Participants will be randomised to bisoprolol or placebo. Bisoprolol will be in the form of 1.25mg tablets. Each bottle will contain 168 tablets. The first bottle of study medication (or placebo) will be provided to the participant via the Central Clinical Trials Pharmacy. This first bottle of study medication will be delivered to participants via a courier service (or other signed for delivery service) operated by a third party.

### 7.3.1 Method of implementing the randomisation/allocation sequence

The random allocation sequence will be generated using permuted blocks. This will provide randomly generated blocks of entries of varying sizes permuted for each combination of region and recruitment setting (primary or secondary care).

Each entry will be assigned a treatment according to a randomly generated sequence utilising block sizes of 2 or 4. Each treatment option will be assigned an equal number of times within each block, ensuring that the total entries assigned to each treatment remained balanced. The sequence of blocks will be random, so it will not be possible to determine the next treatment to be allocated based on previous allocations made during the randomisation process. The random permuted blocks that define how treatments will be allocated to participants will be created by the CHaRT Programming team during the system development process. The system built to utilise these permuted blocks will be tested by a run of simulated randomisations that allow the outcomes to be cross-checked and validated. Before the randomisation system goes live, enough blocks will be created to ensure entries exist for the maximum expected number of participants across the maximum expected number of centres. However, the randomisation system is flexible enough to allow the option to add further permuted blocks to the list if more are required during the lifetime of the trial. In such circumstances, randomly generated sequences in blocks of 2 and 4 continue to be utilised.

## 7.4 Blinding

To ensure double blinding, study medication/placebo will be identical in terms of appearance, taste, touch and smell and dispensed in numbered containers of identical appearance and labelling. Labelling for both study medication and placebo will have the same batch number and expiry date.

## 7.5 Emergency unblinding

This section of the protocol has been written to be in line with the Sponsor SOP (NHSG/UoA SOP-QA-35 - Unblinding). The decision to unblind a single case should be made when knowledge of an individual's allocated treatment is required:

- to enable treatment of severe adverse event/s, or
- in the event of an overdose
- to enable reporting of a SUSAR

Where possible, requests for emergency or unplanned unblinding of individuals should be made via the Trial Manager based in CHaRT at the University of Aberdeen. Agreement of the Chief Investigator or a delegate will then be sought. However, in circumstances where there is insufficient time to make this request or for agreement to be sought, the treating clinician should make the decision to unblind immediately. Out of hours requests will be made through the BICS on-call consultant (via BICS mobile number). This contact number will be on the emergency card that participants are given to carry (section 7.2.2).

In addition, details of how a patient can be unblinded will be included in the site file and in the patient's medical records.

Unblinding will be done via the IVRS (telephone or web-based application). In the event of failure of the IVRS (for example if the server is down), unblinding will be conducted by the on-call pharmacist based at Aberdeen Royal Infirmary who can be obtained by the switchboard (0345 456 6000).

All instances of unblinding should be recorded and reported in writing to CHaRT by the local investigator, including the identity of all recipients of the unblinding information. CHaRT will inform the sponsor of all unblindings.

Allocation should not routinely be revealed to CHaRT personnel, the Chief Investigator, or members of the research team at the site. In the event of unblinding to report a SUSAR. The Chief Investigator and a member of the trial office team will take responsibility for unblinding and reporting.

The Data Monitoring Committee have the right to receive reports of all serious adverse events (in real time) with associated unblinding information. The Sponsor has agreed that these unblinding requests do not need individual approval from the Sponsor and therefore Sponsor and CI approval is not required to unblind these cases provided the agreed process is followed. A member of the CHaRT programming team will unblind the cases and provide the allocation to the Data Monitoring Committee. The allocation will not be revealed to the Chief Investigator, the trial office team, or members of the research team at the site.

At the end of the 12-month follow-up period, participants (or their GPs) may request to be unblinded in order to inform ongoing treatment decisions. In such cases, the CI will have a telephone conversation with the participant or GP, to include discussion of the methodological and scientific advantages of maintaining the blind until the data is analysed. If the CI is not available to have this conversation, it will be made by another senior member of the trial team. Following this conversation, if the participant or GP still wishes to be unblinded, a request to Sponsor to permit unblinding will be made. If the Sponsor agrees, the unblinding will be done by a member of the CHaRT programming team such that the CI, trial office team and site research team can remain blind to treatment allocation.

### 7.6 Trial assessments

After obtaining written informed consent, participants will be assessed as follows:

- At the recruitment visit; face to face or via telephone/video call
- At 1 week, face to face* (dose titration)
- At 2 weeks, face to face* (dose titration)
- At 3 weeks, face to face* (dose titration)
- At 4 weeks, face to face* (dose titration)
- At 6 months (26 weeks); face to face*
- At 12 months (52 weeks); face to face*
- 1 week after cessation of study medication by telephone
- Post study; examination of hospital and GP records to obtain primary outcome data.

* Whilst the COVID-19 pandemic is ongoing, the recruitment visit can be done as face-to-face, with appropriate precautions in place (PPE, social distancing, hand hygiene etc). All other visits (titration, and 6/12 month follow-up) will be conducted remotely, via telephone/video call. Following a remote assessment, if there are clinical concerns, the site can arrange a face-to-face visit with appropriate precautions in place.

In the event that a participant is unable to complete a scheduled assessment because of an acute illness e.g. exacerbation of COPD, the assessment will be appropriately postponed, to be conducted when the participant is stable, ideally within 4 weeks of the scheduled assessment. Similarly, participants who are unable to attend a scheduled assessment for another reason will have this rearranged, ideally within 4 weeks of the scheduled assessment. Participants unable to attend for face to face assessment at six and twelve months can be

followed up by telephone, a home visit, or sent the questionnaire to compete at home. Similarly, recruitment of patients who, for example have limited mobility or who live some distance from the study site, can be carried out during a home visit. During the COVID-19 pandemic, to reduce footfall in hospitals and GP practices, a face-to-face recruitment visit can be done at home, with appropriate COVID-19 measures in place (PPE, social distancing, hand hygiene).

The schedule for data collection within the study is outlined in Table 2 (overleaf).

Potential participants will be informed in the PIL that they can raise any issues with the study team who will arrange further examination if indicated. If the participant or nurse have concerns, these will be discussed with a medically qualified local investigator and suitable arrangements made for the participant to be seen at a later date.

*Demographic, clinical data*
Demographic, contact, clinical history and clinical examination data will be captured at the recruitment assessment.

*Drug history*
Regular use of prescription drugs will be recorded at recruitment, and the 6 and 12 month assessments.

*Smoking history*
Smoking history will be recorded at recruitment, and at the 6 and 12 month assessments.

*Height*
Height will be measured using clinic stadeometer at recruitment. If a remote assessment is being conducted, self reported height will be recorded.

*Weight*
Weight will be measured using clinic scales at recruitment. If a remote assessment is being conducted, self reported weight will be recorded.

*Heart rate*
Resting heart rate will be recorded at recruitment and the dose titration visits at week 1, 2, 3, 4 and the 6 and 12 month assessments. If a remote assessment is being conducted, these will be measured by the participant using a supplied digital sphygmomanometer.

*Blood pressure*
Blood pressure will be measured by a sphygmomanometer and recorded at recruitment and the dose titration visits at week 1, 2, 3, 4 and the 6 and 12 month assessments. If a remote assessment is being conducted, these will be measured by the participant using a supplied digital sphygmomanometer.

*Number of COPD exacerbations*
The primary outcome measure of the total number COPD exacerbations requiring antibiotics/oral corticosteroids whilst on study medication will be ascertained at the 6 and 12 month assessment. Attempts will be made to check GP records for all participants who do not attend for follow-up at 12 months and who do not complete the questionnaire. Participants will be encouraged to record any exacerbations on a ‘reminder card’ and to bring this to their follow-up assessments.

The ATS/ERS guideline definition of COPD exacerbation will be used: a worsening of patient’s dyspnoea, cough and/or sputum beyond day-to-day variability sufficient to warrant a change in management [46]. The minimum management change will be treatment with antibiotics and/or oral corticosteroids. A minimum of two weeks

between consecutive hospitalisations/start of new therapy is necessary to consider events as separate. Severity will be ascertained for each exacerbation.

An operational classification of exacerbation severity will be used:

- Level I Increased use of their SABA
- Level II Use of OCS and/or antibiotics for exacerbation
- Level III Care by services to prevent hospitalisation
- Level IV Admitted to hospital

It can be challenging to differentiate between a COPD exacerbation and pneumonia. If the participant has been admitted to hospital and a definitive diagnosis is available, it will be possible to determine whether this is captured in the data set as an exacerbation, or as a hospital admission (with the diagnosis pneumonia).

If a participant is treated in primary care, a definitive diagnosis may not be available, unless the participant was referred for a chest x-ray. These cases will be captured as COPD exacerbations on the case-report form.

*Table 2 Schedule of study assessments*

| **Assessment** | **Recruitment** | **1 week** | **2 weeks** | **3 weeks** | **4 weeks** | **Month 6 (face to face)** | **Month 12 (face to face )** | **Post study GP records** | **Post weaning/ final dose** |
|---|---|---|---|---|---|---|---|---|---|
| Assessment of Eligibility Criteria | ✓ | | | | | | | | |
| For women of child-bearing potential – pregnancy test (see section 9.6.1) | ✓ | | | | ✓ | ✓ | ✓ | | |
| Written informed consent | ✓ | | | | | | | | |
| Confirmation that participant is content to continue in the trial | | ✓ | ✓ | ✓ | ✓ | ✓ | | | |
| Demographic data, contact details | ✓ | | | | | | | | |
| Clinical history | ✓ | | | | | | | | |
| Drug history | ✓ | | | | | ✓ | ✓ | | |
| Smoking status | ✓ | | | | | ✓ | ✓ | | |
| Height* | ✓ | | | | | | | | |
| Weight* | ✓ | | | | | | | | |
| Pulse/heart rate** | ✓ | ✓ | ✓ | ✓ | ✓ | ✓ | ✓ | | |
| Blood pressure** | ✓ | ✓ | ✓ | ✓ | ✓ | ✓ | ✓ | | |
| Total number COPD exacerbations requiring OCS/antibiotics | | | | | | ✓ | ✓ | ✓ | |
| Hospital admissions | | | | | | ✓ | ✓ | ✓ | |
| Health related quality of life | ✓ | | | | | ✓ | ✓ | | |
| Disease related health status (CAT, BDI/TDI dyspnoea) | ✓ | | | | | ✓ | ✓ | | |
| HARQ (some centres only) | ✓ | | | | | ✓ | ✓ | | |
| Post bronchodilator lung function$^{+}$ | ✓ | ✓ | ✓ | ✓ | ✓ | ✓ | ✓ | | |
| Adverse events/drug reactions | | ✓ | ✓ | ✓ | ✓ | ✓ | ✓ | | |
| Major adverse cardiac events | | | | | | ✓ | ✓ | | |
| Health care utilisation | ✓ | | | | | ✓ | ✓ | | |
| Patient Compliance | | ✓ | ✓ | ✓ | ✓ | ✓ | ✓ | | |
| Telephone call to confirm cessation of study drug | | | | | | | | | ✓ |

* If remote assessment being conducted self reported height and weight will be recorded.
** If remote assessment being conducted these will be measured using a supplied digital sphygmomanometer.
[+] During the COVID-19 pandemic, lung function (an aerosol generating procedure) will not be assessed.

*Hospital admissions*
The number of unscheduled hospital admissions whilst on study medication will be ascertained by questionnaire at the 6 and 12 month assessments. Emergency admissions consequent upon COPD will also be identified. Participants will be encouraged to record any hospital admissions on the reminder card, and bring this to their follow-up assessments. Attempts will be made to check GP records for all participants who do not complete the questionnaire and do not attend for follow-up at 12 months.

*Major adverse cardiovascular events (MACE)*
MACE as defined by cardiovascular death, hospitalisation for myocardial infarction, heart failure, or stroke, percutaneous coronary intervention or coronary artery bypass grafting [47], will be ascertained at the 6 and 12 month assessments. Attempts will be made to check GP records for all participants who do not complete the questionnaire and do not attend for follow-up at 12 months.

*Health related quality of life*
Health related quality of life data will be captured at recruitment, and at the 6 and 12 month assessments using EuroQoL 5D-3L (EQ-5D-5L) Index that has been used widely in COPD [48]. EQ-5D-5L was developed as a utility questionnaire and addresses mobility, self-care, usual activities, pain/discomfort and anxiety/depression. The completed instrument can be translated into quality of life utilities suitable for calculation of QALYs through the published UK tariffs.

*Disease related health status*
Disease related health status will be ascertained at recruitment and at the 6 and 12 month assessments by questionnaire using the COPD Assessment Test (CAT) [49-51]. The CAT is an 8-item unidimensional measure of health status impairment in COPD. The score ranges from 0-40; it correlates very closely with health status measured using the St George Respiratory Questionnaire and is reliable and responsive. The CAT score is preferred since it provides a more comprehensive assessment of the symptomatic impact of COPD.

The Baseline Dyspnoea Index (BDI) questionnaire will be included in the recruitment, assessment and the Transitional Dyspnoea Index (TDI) will be administered at the 6 and 12 month assessments [52]. The research nurse will ask open ended questions about breathlessness, the nurse will then interpret the responses and will select a score. BDI and TDI were developed in order to obtain a comprehensive understanding of patients' severity of breathlessness and are based on three components: functional impairment; magnitude of task; and magnitude of effort [52]. BDI is a discriminative instrument used to quantify the severity of dyspnoea at an initial or baseline state, whereas TDI is an evaluative instrument used to quantify the changes in dyspnoea from the initial or baseline state. [53].

In selected centres, the HARQ will be used to assess symptoms not elucidated by the CAT or dyspnoea index. This is a validated self-administered questionnaire which is responsive to treatment effects [54].

*Post bronchodilator lung function*
During the COVID-19 pandemic, lung function (an aerosol generating procedure) will not be assessed.

Outwith pandemics, lung function will be measured at recruitment, titration visits, and 6 and 12 months using spirometry performed to ATS/ERS standards [55]. Spirometry is a routine part of the clinical assessment of people with COPD. Post bronchodilator (LABA within 8 hours, short acting ß2 agonist within 2 hours) $FEV_1$ and FVC will be measured. If necessary, lung function will be measured 15 minutes after administration of the participant's own SABA. Widely used European Coal and Steel Community predictive equations will be used to

compute predicted values for $FEV_1$, FVC [56]. As described in section 6.1, if spirometry is contraindicated, it should not be carried out.

*Health care utilisation*
Health care utilisation during the previous 6 months will be ascertained at recruitment and the 6 and 12 month assessments (see section 10.5).

*Adverse reactions and serious adverse events*
Adverse reactions and serious adverse events whilst on study medication will be ascertained at the 1, 2, 3 and 4 week titration assessments and the 6 and 12 month assessments. Serious adverse events/drug reactions will be subject to the serious adverse events reporting protocol (see section 9 for further details).

*Compliance*
Compliance with study medication will be assessed at the 1, 2, 3 and 4 week titration assessments and the 6 and 12 month assessments. Participants will also be asked to estimate their compliance at each visit. Outwith pandemics participants will be asked to return empty drug bottles and compliance will be calculated by pill counting, however during pandemics this will not be possible. Instead, participants will be asked to return any unused study medication to their local pharmacy and ask them to safely dispose of the medication. In these circumstances, no pill count will be possible.

*Mortality*
Deaths during the follow-up period will be reported as a SAE.

## 7.8 Long term follow-up assessments

Participants will be contacted by telephone at an appropriate number of weeks after the end of the treatment period to confirm that they have successfully weaned off study medication, this will be recorded in the CRF.

- Participants on 4 tablets a day will be contacted after 4 weeks
- Participants on 3 tablets a day will be contacted after 3 weeks
- Participants on 2 tablets a day will be contacted after 2 weeks
- Participants on 1 tablet a day will be contacted after 1 week

## 7.9 Qualitative assessments

Not applicable

## 7.10 Withdrawal criteria

Participants may be withdrawn from treatment for any of the following reasons:

- Participant withdraws consent for treatment.
- Unacceptable adverse effects.
- Intercurrent illness preventing further treatment.
- Development of serious disease preventing further treatment or any change in the patient's condition that justifies the discontinuation of treatment in the clinician's opinion.
- Development or diagnosis of a condition for which beta-blockers are guideline recommended treatment e.g. myocardial infarction, heart failure with ejection fraction <40%, acute coronary syndrome.

If participants are withdrawn from treatment they will be asked whether they would be willing to remain in the trial and be followed up as per trial schedule or, failing this, to allow routine follow-up data to be used for trial purposes (hospital/GP medical records).

Participants who withdraw consent for study treatment will be advised to wean down the study medication as described in section 8.7.2. Participants withdrawn from treatment for other clinical reasons (i.e. side effects) will not be weaned, but advised to stop study treatment with immediate effect. Weaning of beta-blockers is advised when administered for cardiovascular indications, this is not the case in this trial.

Participants who wish to withdraw from study follow-up should be asked whether they would be willing to consent to allow routine follow-up data to be used for trial purposes (hospital/GP medical records).

Sites should encourage participants withdrawing from treatment to return all unused study medication to the local study centre for safe destruction of unused medication via Clinical Trials Pharmacy (in secondary care) or via Disposal of Unused Medication Boxes or Community Pharmacies (in primary care). If participants do not return unused medication to their local study centre, they should be encouraged to dispose of the medication safely, for example at their local Community Pharmacy. Their responsibilities in terms of safe return or disposal of study medication should be made clear to participants as part of the informed consent process.

### 7.11 Storage and analysis of clinical samples

There are no clinical samples.

### 7.12 End of trial

The end of clinical follow-up for each participant is defined as completion of the follow-up visit at 12 months. The end of clinical follow-up is when the last participant completes their follow-up visit at 12 months. The end of the trial is defined as the end of funding (currently 31 January 2023; but this may be extended and if so, a further amendment to protocol will be made).

The Funder, Investigators and/or the Trial Steering Committee have the right at any time to terminate the study for clinical or administrative reasons.

The end of the trial will be reported to the REC and Regulatory Authority within 90 days, or 15 days if the study is terminated prematurely. The Investigators will inform participants and ensure that the appropriate follow up is arranged for all involved.

A summary report of the trial will be provided to the REC and Regulatory Authority within 1 year of the end of the trial. An end of trial report will also be issued to the funders at the end of funding.

## 8 TRIAL TREATMENTS

This is a double-blind, randomised, placebo-controlled trial comparing bisoprolol with placebo.

### 8.1 Name and description of investigational medicinal product(s)

Bisoprolol fumarate, 1.25 mg tablets, packed in bottles of 168 tablets. The tablets are white, film-coated round, biconvex tablets with a diameter of 9mm.

Placebo tablets will manufactured to be identical in terms of appearance, taste, touch and smell and dispensed in numbered containers of identical appearance and labelling. Labelling for both study medication and placebo will have the same batch number and expiry date.

### 8.2 Regulatory status of the drug

Bisoprolol is licensed for the treatment of angina, hypertension and heart failure in the UK. In BICS bisoprolol will be used ‘off label’ to people with COPD.

The finished product (packaged and labelled) will be provided to the Sponsor by Mawdsleys. Tiofarma will manufacture and package both the bisoprolol and placebo tablets. The bottled product will be labelled by Mawdsleys.

## 8.3 Product Characteristics

Bisoprolol is licensed for the treatment of stable chronic heart failure with reduced systolic left ventricular function.

Bisoprolol is being used in this study outwith its licensed indication, i.e. off label.

The expected side effects of bisoprolol are listed in table 3 [35]. Side effects will be minimised by starting the study drug at a low dose and slowly up titrating to 5mg once a day (4 tablets) or the maximal tolerated dose.

*Table 3: Expected side effects of bisoprolol*

| System | Side effect | Incidence |
|---|---|---|
| Cardiac | Bradycardia<br>Worsening of heart failure<br>AV-conduction disturbances | Very common (≥1/10)<br>Common (≥1/100, <1/10)<br>Uncommon (≥1/1,000, <1/100) |
| Nervous system | Dizziness, headache<br>Syncope | Common (≥1/100, <1/10)<br>Rare (≥1/10,000, <1/1,000) |
| Gastrointestinal | Nausea, vomiting, diarrhoea, constipation | Common (≥1/100, <1/10) |
| Vascular | Coldness/ numbness in the extremities.<br>Hypotension. | Common (≥1/100, <1/10)<br>Common (≥1/100, <1/10) |
| General | Asthenia, fatigue. | Common (≥1/100, <1/10) |
| Respiratory | Worsening airflow obstruction<br>Allergic rhinitis | Uncommon (≥1/1,000, <1/100)<br>Rare (≥1/10,000, <1/1,000) |
| Musculoskeletal | Muscle weakness, muscle cramps | Uncommon (≥1/1,000, <1/100) |
| Psychiatric | Sleep disorders, depression<br>Nightmares, hallucinations | Uncommon (≥1/1,000, <1/100)<br>Rare (≥1/10,000, <1/1,000) |
| Eyes | Reduced tear flow<br>Conjunctivitis | Rare (≥1/10,000, <1/1,000)<br>Very rare (<1/10,000) |
| Ears | Hearing disorders | Rare (≥1/10,000, <1/1,000) |
| Skin | Itching, flush, rash.<br>Induce psoriasis-like rash<br>Alopecia | Rare (≥1/10,000, <1/1,000)<br>Very rare (<1/10,000)<br>Very rare (<1/10,000) |
| Hepatobiliary | Hepatitis | Rare (≥1/10,000, <1/1,000) |
| Reproductive | Potency disorders | Rare (≥1/10,000, <1/1,000) |

## 8.4 Drug storage and supply

The storage advice for bisoprolol is to store below 25°C and to protect from light. Stability data from the manufacturer shows that bisoprolol is stable at higher temperatures.

After manufacture, the study drug and placebo will be stored at Mawdsleys and shipped to the central Clinical Trials Pharmacy. There will be no requirement for temperature monitoring whilst the study drug and placebo are in transit to the central Clinical Trials Pharmacy. Drugs stored at Mawdsleys and at the central Clinical Trials Pharmacy will be temperature monitored, with any drug packs subject to temperature deviations reported to the trial office and quarantined immediately. A decision will be taken as to whether affected drug packs are safe to use and can be removed from quarantine, or whether they should be destroyed.

Drug packs will be sent by courier (or other signed for delivery service) to participants, with signature on receipt. Participants will be advised to store their medication below 25°C but there will be no temperature monitoring after dispatch of study medication to participants.

The Sponsor will provide bisoprolol and placebo tablets for the purposes of this study. All medication will be provided to the central Clinical Trials Pharmacy; initial shipments and re-orders will be automatically triggered. The central Clinical Trials Pharmacy will dispatch medication to trial participants. All dispatches will be automatically triggered. The central Clinical Trials Pharmacy will maintain accountability records.

Following a participant's recruitment to the trial, the PI (or a medically qualified delegate) will sign a prescription for the relevant drug pack. This prescription will be transmitted to the BICS trial office and then forwarded to the central Clinical Trials Pharmacy to dispense the medication. It will then be couriered by the BICS trial office to the participant's home address.

During the titration period the dose of study medication will be titrated to 1.25mg, 2.5mg, 3.75mg or 5mg. The CI, PI (or a medically qualified delegate) will be confirm the titrated dose which will trigger further supplies of study medication from the central Clinical Trials Pharmacy. As with the first supply, the BICS trial office will courier the study medication to the participant's home address.

Unused study medication may be returned by trial participants to staff at study recruitment sites. This will be returned to local Clinical Trial Pharmacies for destruction, or safely disposed via appropriate waste streams, Community Pharmacies or in "*disposal of unused medication*" facilities. Where unused study medication is returned to staff at study recruitment sites, destruction should be documented at the recruitment site on the case report form.

Post-trial access to IMP
At the end of the study we will inform the participants and their GPs of the results of the study and their allocation status. However, we do not plan to unblind individual participants at the end of their individual period of clinical follow-up (i.e. 12 months). Thus, participants will not be told whether they were taking bisoprolol at the end of their clinical follow up. Participants will be reminded that bisoprolol is not licensed for the treatment of COPD and we would not advise its use until its therapeutic role is established. However, if a participant wishes to take bisoprolol, the GP can be advised of this by letter. In the letter we will indicate to the GP that this would be outwith the licence for bisoprolol, that the patient may have been on placebo or bisoprolol and that dose-titration would be required.

## 8.5 Preparation and labelling of Investigational Medicinal Product

The Sponsor has entered into a supply agreement with Mawdsleys to supply packaged and labelled bisoprolol fumarate and placebo for the purposes of the BICS study. The product will be provided in tamper proof bottles containing 168 tablets. Prior to release to the Sponsor, the supplier (Mawdsleys) will label the bottles of bisoprolol fumarate and placebo with annex 13 compliant labels.

The tablets are provided ready for use – no preparation is required for the bisoprolol fumarate or placebo.

## 8.6 Dosage schedules

The dose of both the active and control interventions will be that established during the four to seven week period of dose titration (study assessments at randomisation, and at approximately 1, 2, 3 and 4 weeks).

The dose to be taken for the remaining 48 week treatment period will either be bisoprolol 5mg once a day orally (placebo 4 tabs a day) or the maximal tolerated dose of bisoprolol/placebo (whichever is the lower).

Treatment duration is 12 months.

Study drug will be weaned off in the weeks following the final study assessment at 52 weeks.

## 8.7 Dosage modifications

### 8.7.1 Dose titration

The dose titration schedule is a conservative interpretation of the advice provided in Heart Failure guidelines, the SmPC for bisoprolol and NHS Grampian heart failure guidelines designed for use by appropriately trained nurses in primary care settings [34-37]. The dose titration is outlined in Figure 3.

*General considerations:*

- As for heart failure, bisoprolol will be introduced in a 'start low, go slow' manner, with assessment of heart rate, blood pressure, $FEV_1$ and clinical status after each titration. During the COVID-19 pandemic, $FEV_1$ will not be assessed – rather the participants will be asked about any changes in their breathlessness occurring after starting/increasing study drug.
- Ideally dose titration will take place during the first seven weeks of the treatment period. However, as in everyday clinical practice, practical (e.g. time for participant to receive study treatment, participant availability, holidays) and clinical considerations (e.g. side effects, exacerbations) a degree of flexibility and clinical judgement is likely to be needed. The timing of dose titration visits must be 7 days or more apart. In most cases, the dose established by the end of week 7 after randomisation will be considered the dose for the remaining treatment period; but there will be some cases where this period is extended (for example due to frequent exacerbations during this period). The first study bottle containing 168 tablets provides sufficient for up to 14 days between each titration visit.
- Prior to commencing treatment participants will be advised:
  - not to expect immediate improvement and that any benefit is likely to be reflected in reduced exacerbations;
  - temporary symptomatic deterioration (fatigue, tiredness) may occur during the initiation/up titration phase but this can usually be easily managed by adjustment of bisoprolol therapy (usually to previous tolerated dose)
  - not to stop treatment without first consulting their research team.
- It will not be possible to reliably establish in an individual the treatment allocation from heart rate, blood pressure. In studies of bisoprolol in heart failure similar proportions of patients allocated to placebo and bisoprolol were unable to tolerate treatment [38].
- Once established on a treatment dose, no further titration assessments will be required.
- Once established on a specific treatment dose, it may be necessary to reduce dose either temporarily or for the remaining treatment period.
- Treatment should not be abruptly stopped (unless clinically indicated), but weaned off by one tablet a day per week to zero. Successful weaning will be confirmed by a telephone call from local research team.
- If participant exacerbates after commencing the dose titration schedule, their dose will be reduced to the previous tolerated dose prior to exacerbation. Dose titration should resume once exacerbation has resolved. If the exacerbation occurs in the first week of titration, the drug should be stopped and the titration process restarted 4 weeks after recovery from the exacerbation. Participants exacerbating during dose titration should be discussed with CI/Senior Trial Manager as changes in the dates of supply of the study drug may be required.

*Specifics*

Participants will be commenced on bisoprolol 1.25mg (1 tablet) od or placebo (1 tablet) od at the recruitment/randomisation assessment.

The dose of bisoprolol will be increased weekly 1.25mg→2.5mg→3.75mg→5mg resulting in final doses of 1.25mg od (1 tablet), 2.50 mg od (2 tablets), 3.75mg od (3 tablets), or 5mg od (4 tablets) depending on tolerance to bisoprolol up-dosing.

The dose of placebo will be increased weekly 1 tablet→2 tablets→3 tablets→4 tablets resulting in final doses of 1, 2, 3 or 4 tablets a day depending on tolerance to up-dosing.

Approximately one week after starting study treatment, and approximately one week after each dose titration assessment (i.e. at week 1, week 2, week 3, week 4), heart rate, blood pressure, $FEV_1$ (during the COVID-19 pandemic, patient reported changes in breathlessness will be used in place of $FEV_1$) and clinical status (side effects) will be assessed.

**Study drug dose will be increased as per dose titration schedule (1 tablet→2 tablets→3 tablets→4 tablets) if:**
- participant is not complaining of intolerable side effects,
- heart rate is ≥60 bpm,
- systolic BP ≥100mmHg and
- FEV1 has not significantly declined in the absence of an exacerbation. In place of FEV1, during the COVID-19 pandemic, patients will be asked about changes in breathlessness, and if breathlessness has worsened, the dose will not be increased.

**Study drug will NOT be increased in line with dose titration schedule if any of the following occurs:**
1. Participant complains of marked symptoms potentially attributable to bisoprolol e.g. intolerable fatigue: dose will be reduced to the previous dose in the dose titration schedule, i.e. 1.25mg to 0, 2.5mg to 1.25mg, 3.75mg to 2.5mg, 5mg to 3.75mg. Participants will be reviewed approximately one week later as per the dose titration protocol.
2. Participant has a significant decline in $FEV_1$ (defined as drop in post-bronchodilator $FEV_1$ of >15% and 200ml from baseline in the absence of an exacerbation), study drug dose will be reduced to the previous dose in the dose titration schedule, i.e. 1.25mg to 0, 2.5mg to 1.25mg, 3.75mg to 2.5mg, 5mg to 3.75mg. During the COVID-19 pandemic, if participants report worsening breathlessness since the increase in study drug dose (in the absence of an exacerbation), the study drug dose will be reduced. Participants will be reviewed approximately one week later as per dose titration protocol.
3. Heart rate 50-59 bpm and/or systolic BP <100mmHg and asymptomatic (no dizziness, no light headedness etc) then study drug dose will be unchanged.
   Heart rate <50 bpm and/or systolic BP <100mmHg and symptomatic (dizzy, light headed etc), dose will be reduced to the previous dose in the dose titration schedule, i.e. 1.25mg to 0, 2.5mg to 1.25mg, 3.75mg to 2.5mg, 5mg to 3.75mg. If severely symptomatic consideration will be given to stopping study drug. Heart rate <45 bpm then an ECG will be performed to exclude second/third degree heart block. During the COVID-19 these parameters will be measured by the participant using the digital sphygmomanometer supplied by the study.

Participants will be reviewed a week later as per dose titration protocol.

At each titration visit, data on side effects, heart rate, systolic BP and $FEV_1$ (or during the COVID-19 pandemic, breathlessness) will be entered into the eCRF. The website will contain an algorithm to either advise increase dose, maintain current dose or reduce dose. Once the maximum dose is established, or if not established, at the end of week 7 (unless this is extended, see above), the algorithm will generate an order for resupply of study medication. This will be transmitted to the central Clinical Trials Pharmacy and the appropriate medication dispatched to the participant.

### 8.7.2 Dose reduction at end of follow-up

Following completion of the 12 month dosing period participants will be weaned off study drug over the following weeks (3-2-1 tablet(s) od) in order to avoid possible rebound myocardial ischemia. A similar regime will be recommended for participants who wish to discontinue medication during the study.

| *Maintenance dosage:* | *At final visit* | *Subsequent week* 1 | 2 | 3 |
|---|---|---|---|---|
| 1 tab | Stop | | | |
| 2 tab | 1 tab | stop | | |
| 3 tab | 2 tab | 1 tab | stop | |
| 4 tab | 3 tab | 2 tab | 1 tab | stop |

Successful weaning will be confirmed by the research nurse by telephone one week after anticipated cessation of treatment. Successful weaning is defined as reduction of dose as described above prior to stopping. Any adverse effects experienced during weaning will be documented within the CRF.

*Figure 3: Schematic representation of dose titration*

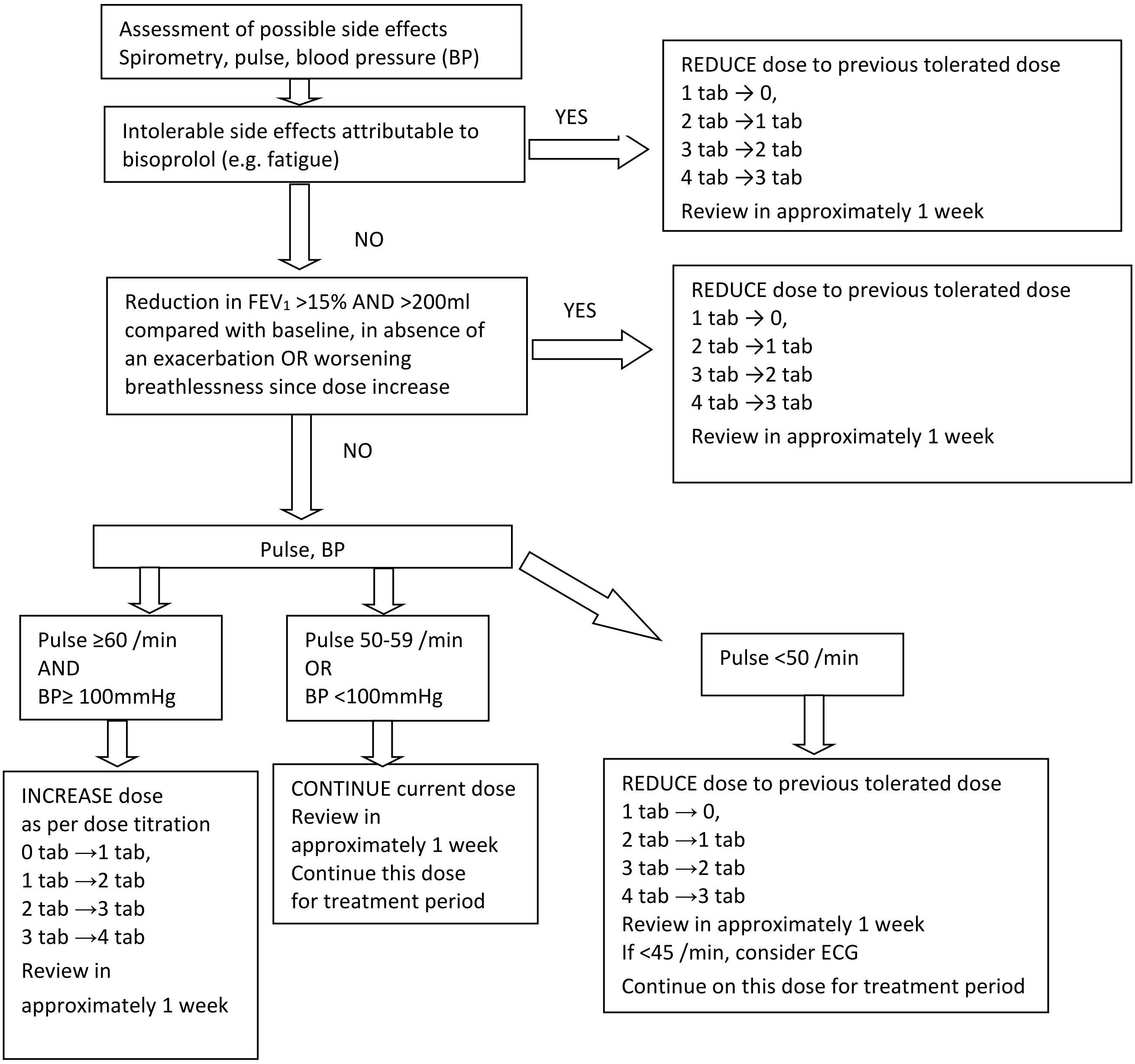

### 8.8 Known drug reactions and interaction with other therapies

A number of drugs are known to interact with beta-blockers to potentiate chronotropic and inotropic effects and are not recommended [35]. There are several medications where caution is advised, due caution will be taken by the use of the 'start low, go slow' dose titration schedule.

Information about medications that interact with bisoprolol is included in the GP letters that are sent out after recruitment and after the six month follow-up appointment. GPs and secondary care physicians are familiar with the use of beta-blockers. The emergency unblinding card that participants are given asks them to show this to any doctor prescribing medication. Advice is included on the card to doctors to try and avoid drugs that interact with bisoprolol, with a link to the BICS study website (which has a list of such medication and what to do if they wish to prescribe an interacting medication).

*Medications known to significantly interact with bisoprolol*

- Calcium antagonists of the verapamil type and to a lesser extent of the diltiazem type: Negative influence on contractility and atrio-ventricular conduction.
- Ivabradine: Negative influence on atrio-ventricular conduction.
- Class I antiarrhythmic drugs (e.g. quinidine, disopyramide; lidocaine, phenytoin; flecainide, propafenone): Effect on atrio-ventricular conduction time may be potentiated and negative inotropic effect increased.
- Centrally acting antihypertensive drugs such as clonidine and others (e.g. methyldopa, moxonodine, rilmenidine): Concomitant use of centrally acting antihypertensive drugs may worsen heart failure by a decrease in the central sympathetic tone (reduction of heart rate and cardiac output, vasodilation). Abrupt withdrawal of the antihypertensive drug, particularly if prior to beta-blocker discontinuation, may increase risk of "rebound hypertension".

These medications are listed in the eligibility criteria. Participants who are known to be taking one or more of these medications at baseline are not eligible for the study and should not be recruited.

If for clinical reasons a beta-blocker or a medication known to significantly interact with bisoprolol is deemed necessary then the study treatment will be discontinued and appropriate treatment instigated by the caring physician, no weaning will be required because of clinical necessity (although weaning can be carried out at the discretion of the treating clinician). Participants will be asked to remain in the study and be followed up in accordance with the trial protocol.

Despite these precautions, if participants are inadvertently prescribed a medication known to significantly interact with bisoprolol whilst taking BICS medication, this will be logged on a study log of interacting medication held by the trial office. If the participant experiences a serious adverse event (e.g. hospitalisation with complete heart block) as a result of the interaction, this will be recorded as (i) a serious adverse event within the study and assessments of relatedness and expectedness made and (ii) as a breach which would be assessed as serious or non-serious according to the definitions.

*Medications that may interact with bisoprolol and to be used with caution.* Participants on these medications at recruitment are eligible for the study, the dose titration will compensate for any interactions.

- Calcium antagonists of the dihydropyridine type such as felodipine and amlodipine: Concomitant use may increase the risk of hypotension.
- Class-III antiarrhythmic drugs (e.g. amiodarone): Effect on atrio-ventricular conduction time may be potentiated.
- Topical beta-blockers (e.g. eye drops for glaucoma treatment) may add to the systemic effects of bisoprolol.
- Parasympathomimetic drugs: Concomitant use may increase atrio-ventricular conduction time and the risk of bradycardia.

- Insulin and oral antidiabetic drugs: Increase of blood sugar lowering effect. Blockade of beta-adrenoreceptors may mask symptoms of hypoglycaemia.
- Anaesthetic agents: Attenuation of the reflex tachycardia and increase of the risk of hypotension.
- Digoxin: Reduction of heart rate, increase of atrio-ventricular conduction time.
- β-Sympathomimetic agents (e.g. isoprenaline, dobutamine): Combination with bisoprolol may reduce the effect of both agents.
- Concomitant use with antihypertensive agents as well as with other drugs with blood pressure lowering potential (e.g. tricyclic antidepressants, barbiturates, phenothiazines) may increase the risk of hypotension.
- Mefloquine: increased risk of bradycardia
- Monoamine oxidase inhibitors (except MAO-B inhibitors): Enhanced hypotensive effect of the beta-blockers but also risk for hypertensive crisis.

Information will be provided to GPs to monitor pulse/BP if these drugs are prescribed whilst on study medication. If pulse and/or BP fall the study drug will be reduced weekly until satisfactory pulse and BP achieved.

If participants are prescribed a medication that may interact with bisoprolol whilst taking BICS medication, this will be logged on a study log of interacting medication held by the trial office. If the participant experiences a serious adverse event (e.g. hospitalisation with complete heart block) as a result of the interaction, this will be recorded as a serious adverse event within the study and assessments of relatedness and expectedness made.

### 8.9 Concomitant medication

Any respiratory medication is permitted. Intervention and control groups will receive usual NHS care: +/- ICS, +/-long acting ß2 agonist (LABA), +/- long acting antimuscarinic.

Exacerbations of COPD will be treated as usual.

### 8.10 Trial restrictions

The trial restrictions in terms of interactions with other therapies are described in section 8.8.

### 8.11 Assessment of compliance with treatment

Compliance with study medication will be assessed at the 1, 2, 3 and 4 week titration assessments and the 6 and 12 month assessments. Outwith pandemics, participants will be asked to return empty drug bottles. Compliance will be calculated by pill counting. During pandemics this will not be possible. Instead, participants will be asked to return any unused study medication to their local pharmacy and ask them to safely dispose of the medication. In these circumstances, no pill count will be possible. Participants will also be asked to estimate their compliance at each visit.

### 8.12 Name and description of each Non-Investigational Medicinal Product (NIMP)

There are no NIMPs within this trial. During follow-up, participants should continue to take any existing medication, and any new medication prescribed for them (8.10) but these are not supplied to the participant within this protocol.

# 9 PHARMACOVIGILANCE

## 9.1 Definitions

| Term | Definition |
|---|---|
| **Adverse Event (AE)** | Any untoward medical occurrence in a participant to whom a medicinal product has been administered, including occurrences which are not necessarily caused by or related to that product. |
| **Adverse Reaction (AR)** | An untoward and unintended response in a participant to an investigational medicinal product which is related to any dose administered to that participant.<br>The phrase "response to an investigational medicinal product" means that a causal relationship between a trial medication and an AE is at least a reasonable possibility, i.e. the relationship cannot be ruled out.<br>All cases judged by either the reporting medically qualified professional or the Sponsor as having a reasonable suspected causal relationship to the trial medication qualify as adverse reactions. It is important to note that this is entirely separate to the known side effects listed in the SmPC. It is specifically a temporal relationship between taking the drug, the half-life, and the time of the event or any valid alternative etiology that would explain the event. |
| **Serious Adverse Event (SAE)** | A serious adverse event is any untoward medical occurrence that:<br>• results in death<br>• is life-threatening<br>• requires inpatient hospitalisation or prolongation of existing hospitalisation<br>• results in persistent or significant disability/incapacity<br>• consists of a congenital anomaly or birth defect<br>Other 'important medical events' may also be considered serious if they jeopardise the participant or require an intervention to prevent one of the above consequences.<br>NOTE: The term "life-threatening" in the definition of "serious" refers to an event in which the participant was at risk of death at the time of the event; it does not refer to an event which hypothetically might have caused death if it were more severe. |
| **Serious Adverse Reaction (SAR)** | An adverse event that is both serious and, in the opinion of the reporting Investigator, believed with reasonable probability to be due to one of the trial treatments, based on the information provided. |
| **Suspected Unexpected Serious Adverse Reaction (SUSAR)** | A serious adverse reaction, the nature and severity of which is not consistent with the information about the medicinal product in question set out in the reference safety information:<br>• in the case of a product with a marketing authorisation, this could be in the summary of product characteristics (SmPC) for that product, so long as it is being used within its licence. If it is being used off label an assessment of the SmPCs suitability will need to be undertaken.<br>• in the case of any other investigational medicinal product, in the investigator's brochure (IB) relating to the trial in question |

NB: to avoid confusion or misunderstanding of the difference between the terms "serious" and "severe", the following note of clarification is provided: "Severe" is often used to describe intensity of a specific event, which may be of relatively minor medical significance. "Seriousness" is the regulatory definition supplied above.

The severity of SAEs will be assessed using the following definitions;

- **Mild:** an event that is easily tolerated by the trial participant, causing minimal discomfort and not interfering with everyday activities.
- **Moderate:** an event that is sufficiently discomforting to interfere with normal everyday activities.
- **Severe:** an event that prevents normal everyday activities.

## 9.2 Operational definitions for (S)AEs

In this trial:

- SAEs will be recorded from the time a participant consents to join the study until the end of the weaning period after the 12 month follow-up (see notes in section 8.7.2). Participants who withdraw from taking the study drug during the 12 month follow-up period will have SAEs recorded from the time the participant consents to join the study until 28 days after ceasing study medication. Participants who never start taking the study drug will have SAEs recorded for 28 days from the time they consent to join the study. All adverse reactions will be recorded for the same period of time described above.
- Hospitalisations for treatment planned prior to randomisation and hospitalisation for elective treatment of a pre-existing condition will **not** be considered or recorded or reported as an SAE. Complications occurring during such hospitalisation will also not be considered, recorded or reported as an SAE – unless there is a possibility that the complication arose because of the study medication (i.e. a possible adverse reaction).
- Exacerbations of COPD or hospital admissions as a consequence of exacerbations of COPD will **not** be considered as AEs or SAEs because they are primary and secondary outcomes for the trial. These will be recorded as part of the trial outcomes, but will not be considered or recorded or reported as AEs or SAEs. Confirmed cases of pneumonia should be captured as SAEs.

All other SAEs (including SARs) should be reported to the trial office.

## 9.3 Recording and reporting of SAEs, SARs AND SUSARs

The time-frame over which ARs and SAEs are recorded is described in section 9.2.

ARs are recorded on the case-report forms. If the AR is serious, it will also be captured on the SAE form. SAEs not related to the study medication will also be captured on the SAE form. When an SAE or SAR occurs, it is the responsibility of the Investigator to review all documentation (e.g. hospital notes, laboratory and diagnostic reports) related to the event. The Investigator should then record all relevant information on the SAE form. The SAE will collect information on diagnosis, dose, type of event, onset date, Investigator assessment of severity and causality, date of resolution, as well as any treatment required, action taken, investigations needed and outcome.

Once the Investigator becomes aware that an SAE has occurred in a study participant, they must report the information to the Trial Office within 24 hours of becoming aware of the event, the Trial Office will report to the Sponsor within 24 hours of becoming aware of the event as per the current Sponsor SOP (SOP-QA-22).

The SAE form must be completed as thoroughly as possible (either as a hard copy CRF or electronically on the trial website) with all available details of the event, and signed by the Investigator or designee. The completed SAE form should then be faxed or emailed to the study office.

If all the required information is not available at the time of reporting, the Investigator must ensure that any missing information is provided as soon as this becomes available. It should be indicated on the report that this information is follow-up information of a previously reported event.

For the purposes of this study, the Reference Safety Information used in the assessment of expectedness is section 4.8 of the SmPC for bisoprolol. This is can be found on the study website.

All SAEs assigned by the PI or delegate (or following central review) as both suspected to be related to IMP-treatment and unexpected will be classified as SUSARs and will be subject to expedited reporting to the

Medicines and Healthcare Products Regulatory Agency (MHRA). The sponsor will inform the MHRA, the REC and Marketing Authorisation Holder (if not the sponsor) of SUSARs within the required expedited reporting timescales.

### 9.4 Responsibilities

Principal Investigator (PI):

- Checking for ARs and SAEs when participants attend for treatment / follow-up. This may be delegated to other members of the research team at site.
- Using medical judgement in assigning seriousness, causality and whether the event/reaction was anticipated using the Reference Safety Information approved for the trial.
- Ensuring that all SAEs and SARs are recorded and reported to the trial office within 24 hours of becoming aware of the event and provide further follow-up information as soon as available.
- Ensuring that SAEs and SARs are recorded and reported to the sponsor in line with the requirements of the protocol.

Chief Investigator (CI) / delegate or independent clinical reviewer:

- Clinical oversight of the safety of patients participating in the trial, including an ongoing review of the risk / benefit.
- Using medical judgement in assigning the SAEs seriousness, causality and whether the event was anticipated (in line with the Reference Safety Information) where it has not been possible to obtain local medical assessment.
- Immediate review of all SUSARs.
- Assigning Medical Dictionary for Regulatory Activities (MedDRA) or Body System coding to all SAEs and SARs.
- Preparing the clinical sections and final sign off of the Development Safety Update Report (DSUR).

Trial manager

- Central data collection of ARs, SAEs, SARs and SUSARs according to the trial protocol onto a database.
- Reporting safety information to the CI, delegate or independent clinical reviewer for the ongoing assessment of the risk / benefit.
- Reporting safety information to the independent oversight committees identified for the trial (Data Monitoring Committee (DMC) and / or Trial Steering Committee (TSC)).
- Expedited reporting of SUSARs to the Competent Authority (MHRA in UK) and REC within required timelines.
- Notifying Investigators of SUSARs that occur within the trial.
- The unblinding of a participant for the purpose of expedited SUSAR reporting.
- Checking for updates to the Reference Safety Information for the trial.
- Preparing standard tables and other relevant information for the DSUR in collaboration with the CI and ensuring timely submission to the MHRA and REC.

Trial Steering Committee (TSC):

In accordance with the Terms of Reference for the TSC, periodically reviewing blinded safety data and liaising with the DMC regarding safety issues.

Data Monitoring Committee (DMC):

In accordance with the Terms of Reference for the DMC, periodically reviewing overall safety data to determine patterns and trends of events, or to identify safety issues, which would not be apparent on an individual case basis. The DMC may review safety data in an unblinded fashion.

## 9.5 Notification of deaths

All deaths will be reported to the sponsor irrespective of whether the death is related to disease progression, the IMP, or an unrelated event. The standard SAE form should be used to report deaths. Once the Investigator becomes aware that a death has occurred in a study participant, they must report the information to the Trial Office within 24 hours of becoming aware of the event, the Trial Office will report to the Sponsor within 24 hours of becoming aware of the event as per the current Sponsor SOP (SOP-QA-22).

## 9.6 Pregnancy reporting

The SmPC for bisoprolol states that it should not be used during pregnancy or whilst lactating unless clearly necessary. Current (or planned pregnancy during the study) or current breastfeeding are exclusion criteria, although given the age profile of people with COPD this is unlikely to be a major issue (see section 9.6.1 for contraceptive requirements and pregnancy testing).

Pregnancy is not considered an SAE however the investigator must collect pregnancy information for female trial subjects. The Investigator should record the information on a Pregnancy Notification Form and submit this to the Trial Office within 14 days of being made aware of the pregnancy. In such cases, trial medication should be stopped. Any pregnancy that occurs in a trial subject during a trial should be followed to outcome. In some circumstances, it may be necessary to monitor the development of the newborn for an appropriate period post delivery. Should the trial subject not wish for the pregnancy to followed to outcome or beyond, this should be noted in the CRF and medical notes as appropriate.

### 9.6.1 Contraceptive requirements and pregnancy testing

Women who are eligible to take part in the trial and are of child bearing potential (i.e. are not post-menopausal (defined as 12 months with no menses without an alternative medical cause) or permanently sterilised) will be advised to use two forms of highly effective birth control (i.e. results in a less than 1% per year failure rate) and continue use until at least 28 days after last dose of trial medication. Acceptable forms of contraception include:-

- Established use of oral, transdermal, injected or implanted hormonal methods of contraception.
- Placement of an intrauterine device (IUD) or intrauterine system (IUS).
- Barrier methods of contraception: Condom or Occlusive cap (diaphragm or cervical/vault caps) plus a spermicidal foam/gel/film/cream/suppository.
- Male partner is sterile (with the appropriate post-vasectomy documentation of the absence of sperm in the ejaculate) prior to female entry onto the trial and is the sole partner of the female participant.
- True abstinence: When this is in line with the preferred and usual lifestyle of the subject. [Periodic abstinence (e.g., calendar, ovulation, symptothermal, post-ovulation methods) and withdrawal are not acceptable methods of contraception for trial purposes].

Women who are of child bearing potential should have a pregnancy test seven days or fewer before the first dose of study medication. For women who attend a face-to-face recruitment visit, the pregnancy test can be conducted then. For women recruited without a face-to-face recruitment visit, a pregnancy test can be sent at the same time as the sphygmomanometer and women will be asked to report the results of the pregnancy test during the recruitment assessment. Women of child bearing potential should also have a pregnancy test at the end of titration and at the 6 and 12 month assessments. Women will be sent the pregnancy test around the time of the visit and be asked to report the results of the pregnancy test to the research nurse.

The SmPC for bisoprolol does not warn against the use of bisoprolol in men because of teratogenic effects [35]. No contraception measures are needed for male subjects with pregnant or non-pregnant partner.

### 9.7 Overdose

To date a few cases of overdose (maximum: 2000 mg as a single dose (equivalent to 1600 1.25mg tablets) with bisoprolol have been reported in patients suffering from hypertension and/or coronary heart disease showing bradycardia and/or hypotension; all patients recovered [35]. The maximum total dose of bisoprolol sent out to participants at any one time is 840mg (672 1.25mg tablets).

With overdose (e.g. daily dose of 15 mg instead of 5 mg) third degree AV-block, bradycardia, and dizziness have been reported. In general, the most common signs expected with overdose of a beta-blocking agent are bradycardia, hypotension, bronchospasm, acute cardiac insufficiency and hypoglycaemia.

In general, if overdose occurs, bisoprolol treatment should be stopped and supportive and symptomatic treatment should be provided. Limited data suggest that bisoprolol is hardly dialysable. Based on the expected pharmacologic actions and recommendations for other beta-blocking agents, the following general measures should be considered when clinically warranted.

> Bradycardia: Administer intravenous atropine. If the response is inadequate, isoprenaline or another agent with positive chronotropic properties may be given cautiously. Under some circumstances, temporary pacing may be necessary.
>
> Hypotension: Intravenous fluids and vasopressors should be administered. Intravenous glucagon may be useful.
>
> AV block (second or third degree): Patients should be carefully monitored and treated with isoprenaline infusion or temporary pacing may be necessary.
>
> Heart failure: Administer i.v. diuretics, inotropic agents, vasodilating agents.
>
> Bronchospasm: Administer bronchodilator therapy such as isoprenaline, $beta_2$-sympathomimetic medicinal products and/or aminophylline.
>
> Hypoglycaemia: Administer i.v. glucose.

All overdoses will be reported to the sponsor, overdoses will be evaluated on a case by case basis and decisions to withdraw/retain in the trial cases will be made depending on the degree of unblinding/individual circumstances/participant safety.

### 9.8 Reporting urgent safety measures

If any urgent safety measures are taken the CI/Sponsor shall telephone the MHRA's Clinical Trial Unit as soon as possible after the action is implemented and discuss the issue. Within three days of the date the measures are taken, the CI/Sponsor will give written notice to the MHRA and the relevant REC of the measures taken and the circumstances giving rise to those measures.

### 9.9 The type and duration of the follow-up of participants after adverse reactions.

After initially recording and reporting an AR or an SAE, the Investigator is required to follow each participant as indicated by clinical practice. Follow up information on an SAE should be reported to the Sponsor as per the current SOP (SOP-QA-22).

Section 9.2 of the protocol describes the time-frame over which adverse reactions and SAEs need to be recorded.

### 9.10 Development safety update reports

An annual Development Safety Update Report (DSUR) will be submitted to the MHRA and the main REC listing all SARs and SUSARs. The Chief Investigator is responsible for preparing and submitting annual DSURs to the MHRA and the main REC on the anniversary of the Clinical Trial Authorisation approval.

### 9.11 Incidental findings

Any incidental findings that are clinically important will be communicated to the participant's GP and to the participant themselves.

## 10 STATISTICS AND DATA ANALYSIS

### 10.1 Sample size calculation

The multicentre Evaluation of COPD Longitudinally to Identify Predictive Surrogate Endpoints (ECLIPSE) study reported the frequency of COPD exacerbation in 2138 patients [57]. For patients identical to our target population (≥2 self reported COPD exacerbations in a year requiring antibiotics and/or OCSs), the mean (SD) number of COPD exacerbations within the subsequent one year was 2.22 (1.86), (Dr Nick Locantore, ECLIPSE statistician, personal communication). The Cardiac Insufficiency Bisoprolol Study II (CIBIS -II) reported that the proportion of participants who stop taking study medication in a trial of bisoprolol vs placebo to be about 15% and this is comparable with what has happened in TWICS [38].

Assuming a similar rate in the placebo arm 669 participants are needed in each arm of the trial to detect a clinically important reduction in COPD exacerbations of 15% (i.e. from an average of 2.22 to 1.89) with 90% power at the two-sided 5% significance level. Allowing for an estimated 15% withdrawal from study treatment the aim will therefore be to recruit 787 participants per arm (i.e. 1,574 in total).

### 10.2 Planned recruitment rate

The recruitment period is 36 months. The recruitment projection is shown below (figure 4). The pause in recruitment has impacted on recruitment. The figure below will be updated (at the next protocol amendment) once we have re-established recruitment.

*Figure 4: recruitment projection*

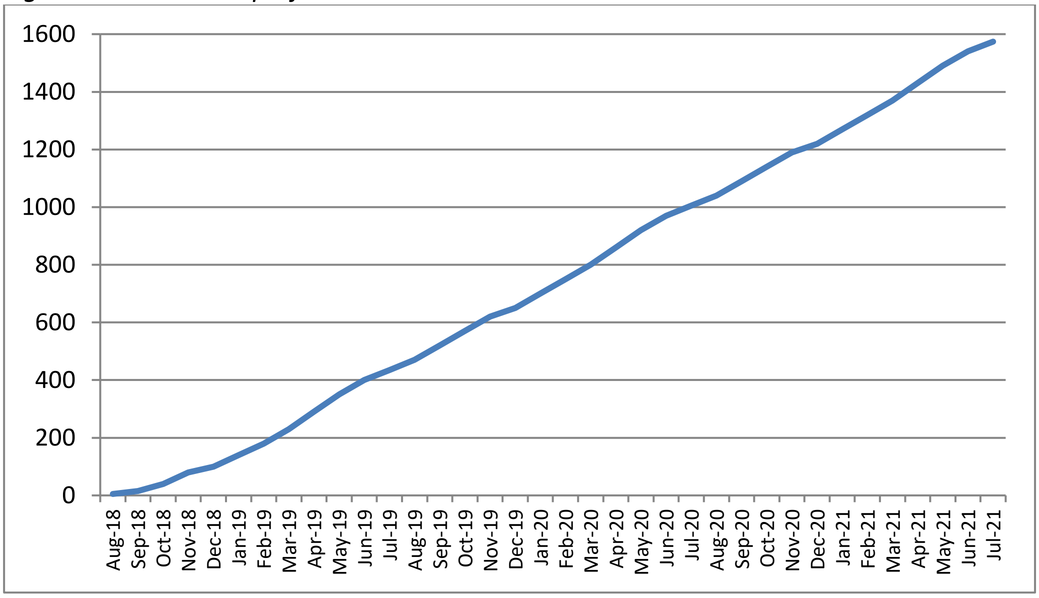

### 10.3 Statistical analysis plan

Statistical analyses will be according to the intention to treat principle with a per protocol analysis performed as a sensitivity. The per protocol analysis will exclude participants who were not compliant (at less than 70%) with their study medication. All analyses will be governed by this comprehensive SAP which will be agreed by the Trial Steering Committee (TSC) and approved by the independent Data Monitoring Committee (DMC) prior to any analyses being undertaken. Unless pre-specified, a 5% two-sided significance level will be used to denote statistical significance throughout. There will be no interim analyses undertaken. In line with other recent COPD trials it is not envisaged that the DMC will be monitoring the study in order to terminate it prematurely on finding evidence of overwhelming benefit – nor will they terminate it for futility.

The primary outcome - number of COPD exacerbations requiring antibiotics and/or oral corticosteroids in the 12 months after randomisation - will be compared between randomised groups using negative binomial regression with length of time in the study as an offset. Estimates will then be adjusted for centre and other baseline covariates that are known to be strongly related to outcome (e.g. age, smoking, COPD hospitalisations in year prior to study – these will be pre-specified in the SAP). Despite the patients being followed-up for a single year, some will withdraw early and, given their chronic illness, some will inevitably die. Use of an overdispersion parameter should improve the model estimates as it takes between patient variability in exacerbation rate into account. The negative binomial model assumes that each patient has their own underlying exacerbation rate which follows a Poisson distribution, but the expected rate is allowed to vary across patients according to a gamma distribution. The shape parameter from the gamma distribution explicitly represents the variability between patients. The negative binomial model coincides with the Poisson model when this dispersion parameter is zero. Assumptions of this model are that exacerbation counts are assumed to vary about means that differ for each patient, with means varying across the population. Hence, the negative binomial model more effectively accounts for increased exacerbation events amongst patients withdrawing early.

To further assess the impact of death (estimated at around 6% of the randomised subjects in the first 12 months after randomisation) on our potential treatment effect, a sensitivity analysis will be undertaken by excluding those subjects who have died. If there is any indication of a differential effect on deaths by treatment, models may be considered that allow the censoring to be informative. For participants that are lost to follow up at some time during the 12 month follow-up (estimated to be around 15% of those randomised), their information will be included in the statistical models up to the point that they are lost to follow up. Sensitivity analyses will be undertaken using multiple imputation (assuming data are missing at random), and, if necessary, and the data permit, specify the mechanism of missing data via a pattern mixture model assuming informative missingness.

The secondary outcomes - total number of COPD exacerbations requiring hospital admission and total number of emergency hospital admissions (all causes) - will each be analysed in the same way as for the primary outcome described above.

Disease related health status (measured using the COPD Assessment Test (CAT)), generic health-related quality of life (EQ5D-5L) and Hull Airways Reflux Questionnaire (HARQ), $FEV_1$ and FVC are each measured at baseline, and at 6 and 12 month follow-up. A mixed effects model will be used to compare each outcome by randomisation group unadjusted and adjusted for centre, patient characteristics and/or baseline clinical variables. Mixed effects models assess rates of change with allowance for the correlation structure of the repeated measures data. Fixed effects will include assessment, treatment, centre and other relevant patient related variables and patient will be fitted as a random effect. A treatment-time interaction will be included to assess the differential treatment effect on rate of change in outcome. Alternative correlation structures will be considered and the most appropriate selected based on the log-likelihood and Akaike's Information Criterion (AIC) of the model. All randomised patients with at least one valid measurement will be included in the analysis and missing outcome data assumed to be missing at random.

As sensitivity analyses, (a) the mixed effects models will be repeated on those patients who survive the 12 month follow-up only (b) where appropriate to do so a pre-specified value will be imputed for those patients who died during follow-up (e.g. zero value for EQ-5D-5L) (c) multiple imputation will be undertaken assuming data are missing at random (d) if missing data are thought to be informative, then consideration will be given to the use of appropriate models such as the pattern mixture model.

Descriptive statistics for health care utilisation and the occurrence of adverse events will be produced and, if appropriate, a comparison will be made between randomised groups using the chi-squared test, t-test etc. Poisson regression may be used to compare multiple events of the same type between randomisation groups. All-cause mortality rate and time to first COPD exacerbation, will be compared between randomised group using a log-rank test and Kaplan-Meier survival curves. Adjustment for potential covariates will be undertaken using Cox proportional hazards regression.

Compliance will be assessed by pill counting of the returned containers at each of the follow up visits and the proportion of compliant patients compared between randomisation groups using the chi-squared test. Major adverse cardiovascular events (MACE) in randomisation groups will be compared using the chi-squared test.

### 10.4 Interim analysis and criteria for the premature termination of the trial

There will be no interim analyses undertaken.

### 10.5 Economic evaluation

An NHS economic evaluation will be conducted in two stages. Firstly, the cost-effectiveness of treatment will be calculated for the within trial period based on observed data. Secondly, the results of the trial will be extrapolated to patient lifetimes using cost-effectiveness modelling.

The within trial analysis will make use of the health care resource use data (translated to a cost-per-patient using unit costs standard reference sources), the exacerbation rate associated with the treatment arms, and the quality of life effects estimated from the EQ-5D-5L combined with utility data to calculate QALYs. Non-parametric bootstrapping will be used to capture sampling uncertainty in the observed data and results will be presented as cost-per-exacerbation avoided and cost-per-QALY gained within the trial period with accompanying confidence intervals (or cost-effectiveness acceptability curves if more appropriate).

The extrapolation analysis will make use of regression estimates of exacerbation on cost and quality of life from the trial, as well as previously published models of COPD, to guide the extrapolation to patient lifetimes. In addition to sampling uncertainty, extensive sensitivity, analysis will be performed to understand the importance of alternative modelling assumptions for the extrapolated results.

*Collection of data:* Data collection from the trial will focus on estimating the use of primary or secondary NHS health care resources.

*Participant Costs:* Direct health care costs falling on the participant costs will not be included. It is not anticipated that these costs will be substantial and, in any case, fall outside of the health service reference case for this analysis.

*NHS Costs of Health Services Used:* Health care utilisation during the previous 6 months will be ascertained at recruitment and the 6 and 12 month assessments by a modified version of the Client Service Receipt Inventory (CSRI) [58]. The CSRI is a research questionnaire for retrospectively collecting cost-related information about participant's use of health and social care services.

*Cost Effectiveness:* Following a method previously used in large international trial for COPD treatments [59],

the within trial analysis will use regression techniques to simultaneously capture heterogeneity of cost-effectiveness among the patients studied, while adjusting for any missing data.

# 11 DATA MANAGEMENT

## 11.1 Data collection tools and source document identification

***Source data***

The primary outcome is exacerbations of COPD necessitating changes in management as reported by the participant. For many of our secondary outcomes (total number of COPD admissions requiring hospital admission, episodes of pneumonia, emergency hospital admissions (all causes), serious adverse events, adverse reactions, MACE, utilisation of primary or secondary health care for respiratory events) the primary source will also be patient-report, although additional details may be available from other sources (including medical records). The health status outcomes are patient reported.

At baseline and at follow-up, study data can be collected on hard copy case report form or entered directly into the study website.

- If hard copy case report forms are completed, these are considered to be the source document. These will then be entered by the local study team onto the study website.
- If the data is entered directly into the study website, the electronic record is considered to be the source document. In order to maintain a copy of the data that is independent from the sponsor copy, sites will be encouraged to print or save a copy of the electronic data. The study website will provide this facility.

Each website user will have their own user account and password. These must not be shared. The study website has a full audit trail and every data entry made (or changed) is logged to the specific user.

For all case report forms, there is an electronic record (as part of the study website) which indicates whether the case report form was completed online (no paper copy) or not. This will allow identification of the source document.

Participants will complete questionnaires at baseline and at 6 and 12 month follow-up. The hard copy of these questionnaires will be considered the source document.

***Case report forms***

The Trial Inclusion Form will be completed as a paper CRF before entering onto the study website. This permits signature from a medical doctor to confirm eligibility of the participant. For other case report forms, site staff can complete paper CRF before entry onto the eCRF or bypass the paper CRF and enter data directly onto the eCRF.

## 11.2 Data handling and record keeping

The electronic data capture system (eCRF) is validated, maintains a full audit trail of data changes, is secure (requiring unique usernames and passwords), and has regular back-up. The system safeguards the blinding of trial data. Participants have a unique participant identification number that allows identification of all data reported for each participant.

The Trial Office will raise data queries with sites. These should be resolved by the site in a timely fashion.

### 11.3 Access to Data

Access to the study websites where data is held is password protected. Site staff with access to the study website can only access the records for their own participants. Staff in the trial office can access records for all participants.

Direct access to records will be granted to authorised representatives from the Sponsor, host institution and the regulatory authorities to permit trial-related monitoring, audits and inspections, in line with participant consent.

### 11.4 Archiving

All study documentation will be kept for at least 25 years after publication of the study data. Copies of consent forms will be forwarded to the BICS trial office on a regular basis. At the end of each participant's follow-up, case report forms and questionnaires will be returned for archiving in Aberdeen. The site files will be archived at each site.

Data will be archived by CHaRT for at least 25 years after publication of the study data.

## 12 MONITORING, AUDIT & INSPECTION

The Sponsor will prepare a Sponsor Monitoring Plan at the outset of the study. A monitor, designated by the Sponsor, or an appointed local monitor will visit the Aberdeen study site prior to the start of the study, during the course of the study and will undertake a close down visit at study end.

The Trial Office will carry out remote central monitoring of accumulating data and consent forms from all sites. As part of the monitoring of consent forms, checks will ensure that the person seeking consent is listed in the delegation log for that site. Anomalies may trigger on-site monitoring at any of the study sites.

A Trial Office Monitoring Plan will be developed and agreed by the Trial Management Group (TMG), TSC and CI based on the trial risk assessment. This will be dependent on a documented risk assessment of the trial. A copy of the monitoring plan will be filed in the Trial Master File.

In addition, the Trial Office will facilitate monitoring by local R&D departments at each of the sties, should this be requested.

## 13 ETHICAL AND REGULATORY CONSIDERATIONS

### 13.1 Research Ethics Committee (REC) review & reports

The study will be conducted in accordance with the principles of Good Clinical Practice (GCP).

A favourable ethical opinion (for the protocol and associated documents) will be obtained from the appropriate REC will be obtained prior to commencement of the study. Sites will also be required to issue local NHS R&D approval/permission or confirm capability and capacity for the study.

Any changes in research activity, except those necessary to remove an apparent, immediate hazard to the participant, must be reviewed and approved by the Sponsor. Amendments to the protocol must be submitted in writing to the appropriate REC, Regulatory Authority and local R&D for approval prior to participants being enrolled into an amended protocol. Substantial amendments that require review by REC will not be implemented until the REC grants a favourable opinion for the trial (note that amendments may also need to be reviewed and accepted by the MHRA and/or NHS R&D departments before they can be implemented in practice at sites).

Amendments to other documentation should not be implemented until appropriate approvals are in place.

Any change to the version of the SmPC used as the Reference Safety Information for the trial will be subject to approval via a substantial amendment.

All correspondence with the REC will be retained in the Trial Master File. An annual progress report (APR) will be submitted to the REC within 30 days of the anniversary date on which the favourable opinion was given, and annually until the trial is declared ended. The Chief Investigator will notify the REC of the end of the trial, including if it is ended prematurely. Within one year after the end of the trial, the Chief Investigator will submit a final report with the results, including any publications/abstracts, to the REC

## 13.2 Peer review

This trial has undergone extensive internal and external peer review as part of the funding process, the protocol has been subject to internal peer review.

All reports of work arising from the BICS trial including conference abstracts should be peer reviewed by the Project Management Group prior to submission – for further details see Appendix 4.

## 13.3 Public and Patient Involvement

Chest Heart and Stroke Scotland (CHSS) and the British Lung Foundation (BLF) actively contributed to the funding application. Before they passed away, a person with COPD was one of the grant holders and a member of the TSC. At the outset of the study, the Voices Scotland Lead for CHSS established a virtual panel of 15 people with COPD who scrutinised participant facing documentation and recommended many changes that have been implemented and returned to the panel. The Voices Scotland Lead for CHSS has left the organisation. The Voices lead for Scotland and a person with COPD were members of the TSC. We are looking to replace their expertise on the committee.

CHSS and BLF will assist in promoting and dissemination of the trial.

## 13.4 Regulatory Compliance

The study will not commence until a Clinical Trial Authorisation (CTA) is obtained from the appropriate Regulatory Authority. The protocol and study conduct will comply with the Medicines for Human Use (Clinical Trials) Regulations 2004, and any relevant amendments.

## 13.5 Protocol compliance

The Investigator should not implement any deviation from the protocol without agreement from the Chief Investigator and appropriate REC, Regulatory Authority and R&D approvals except where necessary to eliminate an immediate hazard to trial participants.

In the event that an Investigator needs to deviate from the protocol, the nature of and reasons for the deviation should be recorded in the CRF. If this necessitates a subsequent protocol amendment, this should be submitted to the REC, Regulatory Authority and local R&D for review and approval if appropriate. Accidental protocol deviations can happen at any time. They must be adequately documented on the relevant forms and reported to the Trial Office within 24 hours. Deviations from the protocol which are found to frequently recur are not acceptable, will require immediate action and could potentially be classified as a serious breach.

## 13.6 Notification of Serious Breaches to GCP and/or the protocol

Any breaches that occur at a trial site should be reported by the trial team at the site to the trial office within 24 hours using the breach report form. The trial office will inform the Chief Investigator and Sponsor within 24 hours.

A “serious breach” is a breach which is likely to effect to a significant degree –

(a) the safety or physical or mental integrity of the participants of the trial; or
(b) the scientific value of the trial

Where the breach is assessed as serious, the Sponsor will notify the MHRA and REC of the serious breach.

### 13.7 Data protection and patient confidentiality

All Investigators and study site staff involved with this study must comply with the requirements of the General Data Protection Regulation (or subsequent legislation), with regard to the collection, storage, processing and disclosure of personal information and will uphold the Act's core principles.

Computers used to collate the data will have limited access measures via usernames and passwords. The study website has limited access measures via usernames and passwords. Staff at sites only have access to participant data for participants at their site. Within the study website, identifiable data is stored with a strong encryption algorithm (currently the key used is AES_256). Participants will be identified using a unique participant identifier.

Data will be stored for at least 25 years after publication of the trial results. The data custodian is the Chief Investigator.

Published results will not contain any personal data that could allow identification of individual participants.

### 13.8 Financial and other competing interests for the chief investigator, PIs at each site and committee members for the overall trial management

The study is funded by the NIHR Health Technology Assessment. The Chief Investigator and study grant holders have no financial or competing interests.

### 13.9 Indemnity

The following arrangements are in place:

| | |
|---|---|
| Arrangements for insurance and/or indemnity to meet the potential legal liability of the sponsor(s) for harm to participants arising from the management of the research | The University of Aberdeen holds and maintains insurance policies, which will provide appropriate compensation. This policy covers Principals, Partners, Directors and employees of the University. |
| Arrangements for insurance and/ or indemnity to meet the potential legal liability of investigators/collaborators arising from harm to participants in the conduct of the research | The University of Aberdeen holds and maintains insurance policies, which will provide appropriate compensation. This policy covers Principals, Partners, Directors and employees of the University. |
| Arrangements for payment of compensation in the event of harm to the research participants where no legal liability arises | The University of Aberdeen holds and maintains a "no fault" insurance policy, which will provide appropriate compensation to research participants up to a limit of £5,000,000. This policy covers Principals, Partners, Directors and employees of the University. |

Digital sphygmomanometers will be provided to sites for participants to assess their heart rate and blood pressure at home.

### 13.10 Amendments

Any changes in research activity, except those necessary to remove an apparent, immediate hazard to the participant, must be reviewed and approved by the Sponsor. Amendments to the protocol must be submitted

in writing to the appropriate REC, Regulatory Authority and local R&D for approval(s) prior to participants being enrolled into an amended protocol.

Amendments to other documentation should not be implemented until appropriate approvals are in place.

Any change to the version of the SmPC used as the Reference Safety Information for the trial will be subject to approval via a substantial amendment.

### 13.11 Post trial care

At the end of the study participants and their GPs will be informed of the results of the study and their allocation status. However, there are no plans to unblind individual participants at the end of their individual period of clinical follow-up (i.e. 12 months). Thus, participants will not be told whether they were taking bisoprolol at the end of their clinical follow up. Participants will be reminded that bisoprolol is not licensed for the treatment of COPD and we would not advise its use until its therapeutic role is established. However, if a participant wishes to take bisoprolol, the GP can be advised of this by letter. In the letter we will indicate to the GP that this would be outwith the licence for bisoprolol, that the patient may have been on placebo or bisoprolol and that dose-titration would be required.

### 13.12 Access to the final trial dataset

At the end of the trial, the statistician and the health economist will have access to the full dataset to permit analysis. Requests for other access to the full dataset will be considered by the Trial Steering Committee.

## 14 DISSEMINATION POLICY

### 14.1 Dissemination policy

The clinical study report will be used for publication and presentation at scientific meetings. Investigators have the right to publish orally or in writing the results of the study. Summaries of results will also be made available to Investigators.

A summary of the study findings will be sent to surviving trial participants (unless they request otherwise).

### 14.2 Authorship eligibility guidelines and any intended use of professional writers

Ownership of the data arising from this study resides with the study team. On completion of the study, the study data will be analysed and tabulated. A detailed report for the funder will be prepared. The publication policy is included in Appendix 4.

## 15 REFERENCES

1. Department of Health. An Outcomes Strategy for COPD and Asthma. (2012). https://www.gov.uk/government/uploads/system/uploads/attachment_data/file/216531/dh_134001.pdf
2. Chronic obstructive pulmonary disease in over 16s: diagnosis and management. https://nice.org.uk/guidance/cg101
3. British Lung Foundation (2015). Health statistics. http://statistics.blf.org.uk/copd
4. Mannino DM, Thorn D, Swensen A, Holguin F. Prevalence and outcomes of diabetes, hypertension and cardiovascular disease in COPD. Eur Respir J 2008; 32: 962–969
5. Holguin F, Folch E, Redd SC, Mannino DM. Comorbidity and mortality in COPD-related hospitalizations in the United States, 1979 to 2001. Chest 2005; 128: 2005–2011.
6. Antonelli-Incalzi R, Fuso L, DeRosa M, et al. Co-morbidity contributes to predict mortality of patients with chronic obstructive pulmonary disease. Eur Respir J 1997; 10: 2794–2800.
7. Rana JS, Mittleman MA, Sheikh J, et al. Chronic obstructive pulmonary disease, asthma, and risk of type 2 diabetes in women. Diabetes Care 2004; 27: 2478–2484.
8. Sin DD, Man JP, Man SF. The risk of osteoporosis in Caucasian men and women with obstructive airways disease. Am J Med 2003; 114: 10–14.

9. Di Marco F, Verga M, Reggente M, et al. Anxiety and depression in COPD patients: the roles of gender and disease severity. Respir Med 2006; 100: 1767–1774.
10. Mannino DM, Doherty D, Aguayo SM, et al. Low lung function and incident lung cancer in the United States: data from the first National Health and Nutrition Examination Survey follow-up. Arch Intern Med 2003; 163: 1475–1480.
11. Mannino DM, Thorn D, Swensen A, Holguin F. Prevalence and outcomes of diabetes, hypertension and cardiovascular disease in COPD. Eur Respir J 2008; 32: 962–969
12. Rutten FH, Cramer MJ, Grobbee DE, et al. Unrecognized heart failure in elderly patients with stable chronic obstructive pulmonary disease. Eur Heart J 2005;26:1887–1894.
13. Macchia A, Rodriguez Moncalvo JJ, Kleinert M, et al. Unrecognised ventricular dysfunction in COPD. Eur Respir J 2012;39:51–58.
14. Donaldson GC, Seemungal TA, Bhowmik A et al. Relationship between exacerbation frequency and lung function decline in chronic obstructive pulmonary disease. Thorax 2002;57:847-52.
15. Donaldson GC, Wilkinson TM, Hurst JR et al. Exacerbations and time spent outdoors in chronic obstructive pulmonary disease. Am J Resp Crit Care Med 2005;171:446-52.
16. Seemungal TA, Donaldson GC, Paul EA et al. Effect of exacerbation on quality of life in patients with chronic obstructive pulmonary disease. Am J Resp Crit Care Med 1998;157:1418-22.
17. Soler-Cataluna JJ, Martinez-Garcia MA, Roman Sanchez P et al. Severe acute exacerbations and mortality in patients with chronic obstructive pulmonary disease. Thorax 2005;60:925-31.
18. McAllister DA, Maclay JD, Mills NL, et al. Diagnosis of myocardial infarction following hospitalisation for exacerbation of COPD. Eur Respir J. 2012;39:1097-1103.
19. Britton M. The burden of COPD in the U.K.: results from the Confronting COPD survey. Respir Med 2003;97:S71-9.
20. Hospital episode statistics (2016). http://www.hscic.gov.uk/searchcatalogue?q=title%3A%22hospital+episode+statistics%22&size=10
21. Du Q, Sun Y, Ding N, et al. Beta-Blockers Reduced the Risk of Mortality and Exacerbation in patients with COPD: A Meta-Analysis of Observational Studies. PLoS ONE 2014; 9(11): e113048. doi:10.1371/journal.pone.0113048
22. Rutten FH, Zuithoff NP, Hak E, et al. Beta-blockers may reduce mortality and risk of exacerbations in patients with chronic obstructive pulmonary disease. Arch Intern Med. 2010;170:880-7.
23. Bhatt SP, Wells JM, Kinney GL, et al. β-Blockers are associated with a reduction in COPD exacerbations. Thorax 2016;71:8-14.
24. Gottlieb SS, McCarter RJ, Vogel RA. Effect of beta-blockade on mortality among high-risk and low-risk patients after myocardial infarction. The New England journal of medicine. 1998;339(8):489-97.
25. Au DH, Bryson CL, Fan VS, et al. Beta-blockers as single-agent therapy for hypertension and the risk of mortality among patients with chronic obstructive pulmonary disease. The American journal of medicine. 2004;117(12):925-31.
26. Short PM, Lipworth SI, Elder DH, et al. Effect of beta blockers in treatment of chronic obstructive pulmonary disease: a retrospective cohort study. Br Med J. 2011;342:d2549.
27. Quint JK, Herrett E, Bhaskaran K, et al. Effect of beta blockers on mortality after myocardial infarction in adults with COPD: population based cohort study of UK electronic healthcare records. Bmj. 2013;347:f6650.
28. Andreas A, Anker SD, Scanlon PD, Somers VK. Neurohumoral activation as a link to systemic manifestations of chronic lung disease. Chest 2005; 128:3618–3624
29. Andreas S, Haarmann H, Klarner S, et al. Increased sympathetic nerve activity in COPD is associated with morbidity and mortality. Lung 2014; 192:235–241
30. Garlichs CD, Zhang H, Mugge A, Daniel WG. Beta-blockers reduce the release and synthesis of endothelin-1 in human endothelial cells. Eur J Clin Invest 1999; 29: 12–16.
31. Dunzendorfer S, Wiedermann CJ. Modulation of neutrophil migration and superoxide anion release by metoprolol. J Molec Cell Cardiol 2000; 32: 915–924.
32. Ghoorah K, De Soyza A, Kunadian V. Increased cardiovascular risk in patients with chronic obstructive pulmonary disease and the potential mechanisms linking the two conditions. Cardiology in Review 2012;21:196–202.
33. Baker JG. The selectivity of beta-adrenoceptor antagonists at the human beta1, beta2 and beta3 adrenoceptors. Br J Pharmacol. 2005;144:317-22.
34. Chronic heart failure in adults: management. Clinical guideline 2010. https://nice.org.uk/guidance/cg108
35. Bisoprolol SmPC 2015. https://www.medicines.org.uk/emc/medicine/25983/SPC/Bisoprolol+2.5mg+5mg+10mg+film+coated+tablet/
36. Scottish Intercollegiate Guidelines Network (SIGN). Management of chronic heart failure. Edinburgh: SIGN; 2016. (SIGN publication no. 147). [March 2016]. Available from URL: http://www.sign.ac.uk
37. NHS Grampian medical Treatment guidelines for chronic heart failure (CHF) (2006). http://www.nhsgchdmcn.scot.nhs.uk/wp-content/heartfailure_full_silo.pdf

38. CIBIS-II Investigators and Committees. The Cardiac Insufficiency Bisoprolol Study II (CIBIS-II): a randomised trial. Lancet 1999; 353: 9–13.
39. Lipworth BJ, Irvine NA, McDevitt DG. A dose-ranging study to evaluate the beta 1-adrenoceptor selectivity of bisoprolol. Eur J Clin Pharmacol. 1991;40(2):135-9.
40. British Thoracic Society/Scottish Intercollegiate Guidelines Network. British guideline on the management of asthma (2014). https://www.brit-thoracic.org.uk/document-library/clinical-information/asthma/btssign-asthma-guideline-2014/
41. Salpeter SR, Ormiston TM, Salpeter EE. Cardioselective beta-blockers for chronic obstructive pulmonary disease. Cochrane Database of Systematic Reviews 2005, Issue 4. Art. No.: CD003566. DOI: 10.1002/14651858.CD003566.pub2
42. Jabbal S, Anderson WJ, Short PM, Morrison AE, Manoharan A, Lipworth BJ. Cardio-pulmonary interactions of beta-blockers and long acting bronchodilators in COPD. (personal communication)
43. Devereux G, Cotton C, Barnes P, et al. Use of low-dose oral theophylline as an adjunct to inhaled corticosteroids in preventing exacerbations of chronic obstructive pulmonary disease: study protocol for a randomised controlled trial. Trials 2015; DOI 10.1186/s13063-015-0782-2
44. Cooper BG. An update on contraindications for lung function testing. Thorax 2011;66:714-723.
45. Quint JK, Donaldson GC, Hurst JR et al. Predictive accuracy of patient-reported exacerbation frequency in COPD. Eur Resp J 2011;37:501-7.
46. Celli BR, MacNee W, ATS/ERS Task Force. Standards for the diagnosis and treatment of patients with COPD: a summary of the ATS/ERS position paper. Eur Resp J 2004;23:932-46.
47. Skali H, Pfeffer MA, Lubsen J, Solomon SD. Variable impact of combining fatal and nonfatal end points in heart failure trials. Circulation 2006; 114: 2298–2304.
48. The EuroQol Group. EuroQol--a new facility for the measurement of health-related quality of life. Health Policy 1990;16:199-208.
49. COPD Assessment Test. http://www.catestonline.org/
50. Dodd JW, Hogg L, Nolan J, et al. The COPD assessment test (CAT): response to pulmonary rehabilitation. A multicentre, prospective study. Thorax 2011;66:425-9.
51. Jones PW, Harding G, Berry P, et al. Development and first validation of the COPD Assessment Test. Eur Resp J 2009;34:648-54.
52. Mahler DA, Weinberg DH, Wells CK, Feinstein AR. The measurement of dyspnea. Contents, interobserver agreement, and physiologic correlates of two new clinical indexes. Chest 1984; 85: 751–758.
53. Witek Jr TJ, Mahler DA. Minimal important difference of the transition dyspnoea index in a multinational clinical trial. Eur Respir J 2003; 21: 267–272.
54. Morice AH, Faruqi S, Wright CE, Thompson R, Bland JM: Cough hypersensitivity syndrome: a distinct clinical entity. Lung 2011, 189:73-79.
55. Miller MR, Hankinson J, Brusasco V, et al. ATS/ERS Task Force. Standardisation of spirometry. Eur Resp J 2005;26 (2):319-38.
56. Quanjer PH, et al. Lung volumes and forced ventilatory flows. Report Working Party Standardization of Lung Function Tests, European Community for Steel and Coal. Official Statement of the European Respiratory Society. Eur Resp J Suppl 1993;16:5-40.
57. Hurst JR, Vestbo, J Anzueto A et al. Evaluation of COPD Longitudinally to Identify Predictive, Surrogate Endpoints. Susceptibility to exacerbation in chronic obstructive pulmonary disease. N Engl J Med 2010;363:1128-38.
58. Barrett B, Byford S. Collecting service use data for economic evaluation in DSPD populations. Br J Psychiatry, 2007, 190 (sup 49), s75-s78.
59. Briggs AH, Glick HA, Lozano-Ortega G et al. Is treatment with ICS and LABA cost effective for COPD? Multinational economic analysis of the TORCH study. Eur Respir J 2010; 35: 532-9.

## APPENDICIES

### Appendix 1 – Risk

Risks associated with trial interventions

☐ A ≡ Comparable to the risk of standard medical care
☑ B ≡ Somewhat higher than the risk of standard medical care
☐ C ≡ Markedly higher than the risk of standard medical care

Bisoprolol is licensed for the treatment of angina, hypertension and heart failure. In BICS bisoprolol will be used 'off label' to people with COPD. Heart failure guidelines state that for people with heart failure and COPD it is safe to use cardiac specific beta-blockers such as bisoprolol to treat heart failure as long as the initiating dose is low and there is a gradual dose titration. Systematic reviews demonstrate that cardiac specific beta blockers such as bisoprolol have no significant effect on lung function, symptoms or response to beta2 agonists when administered to people with COPD.

The main concern is that the administration of bisoprolol will induce bronchospasm (worsening of lung function). The risk of this has been minimised by excluding persons with a sole diagnosis of asthma, a diagnosis of asthma before the age of 40, moreover the dose of bisoprolol will be slowly titrated as recommended for heart failure and the maximum dose (5mg) is less than that recommended for heart failure.

It is anticipated that the side effect profile for bisoprolol will reflect that for use in people with heart disease. In heart disease it is recommended that any cessation of beta-blockers should be slow to prevent relapse/exacerbation of cardiac symptoms, in this trial the dose of bisoprolol will be slowly weaned to zero at the end of the treatment period. For participants with intolerable side effects, wherever possible the dose of bisoprolol will be weaned down to zero wherever possible, if not the study drug will be stopped immediately, this is deemed acceptable as these people have not been commenced on bisoprolol for cardiac disease.

The Chief Investigator, Clinical Trial pharmacist and Sponsor oversight committee have undertaken a review of the licensed vaccines and have confirmed that they would not expect any interaction between the COVID vaccines and the study IMP.

| **What are the key risks related to therapeutic interventions you plan to monitor in this trial?** | | **How will these risks be minimised?** | | |
|---|---|---|---|---|
| IMP/Intervention | Body system/Hazard | Activity | Frequency | Comments |
| Bisoprolol | Respiratory (increasing dyspnoea) | Exclusion criteria<br>Dose titration | When each participant is recruited | |

**Outline any other processes that have been put in place to mitigate risks to participant safety (e.g. DMC, independent data review, etc.)**

All adverse reactions will be captured in the eCRF. In addition all SAEs and SARs will be captured. An independent DMC will review accumulating unblinded safety data.

**Outline any processes (e.g. IMP labelling +/- accountability +/- trial specific temperature monitoring) that have been simplified based on the risk adapted approach.**

IMP will be labelled with Annex 13 compliant labelling. Unused medication that is returned by participants to their study team will be subject to accountability checks. If participants do not return unused medication to their study team, they will be advised to dispose of safely by returning to a pharmacy for destruction.

## Appendix 2 – Authorisation of participating sites

***Required documentation***

In addition to the approvals required (described in section 13), the following documentation is required from sites:

- a signed site agreement
- copies of CVs for relevant members of the site research team;
- copies of GCP certificates for relevant members of the site research team;
- a copy of the trial delegation log for the site.

***Procedure for initiating/opening a new site***

Prior to opening a site, site initiation training will be provided for members of the site team. If not all members of the team can attend the site initiation training, the training can be cascaded by those who have been trained to the rest of the team.

Site initiation training will be carried out by a trial manager or a member of the team (for example a research nurse or member of the primary care network) who has received training from a trial manager and has demonstrated that they can deliver the training competently.

Once training has been carried out and all required documentation is in place, the site-specific greenlight checklist will be completed by a trial manager. The trial manager will open the site on the study website which will permit the site to randomise participants and collect baseline data.

There is no release of drug to participating sites (all drug will be dispatched to trial participants from the Central Clinical Trials Pharmacy).

***Principal Investigator responsibilities***

The PI is responsible for the activity at the site. The PI is required to sign the PI responsibilities within the site agreement. It is expected that the PI is in attendance for at least part of the site initiation training. They are responsible for completing the delegation log.

## Appendix 3 – Safety Reporting Flow Chart

**A member of the local research team becomes aware of a potential SAE**

↓

**Consider whether it needs to be recorded as an SAE within BICS (protocol section 9.2)**

- All deaths should be recorded as an SAE.
- All serious adverse events within the specified timeframe should be recorded, regardless of whether or not they are related to the participant's treatment. However, within BICS, the following do NOT need to be recorded or reported as SAEs:
  - Hospitalisations for treatment planned prior to randomisation and hospitalisation for elective treatment of a pre-existing condition;
  - Complications occurring during such hospitalisation – unless there is a possibility that the complication arose because of the study medication (i.e. a possible adverse reaction);
  - Exacerbations of COPD or pneumonia or hospital admissions as a consequence of exacerbations of COPD or pneumonia

***If you are not sure if an event is an SAE, contact the trial office for advice: 01224 438178***

↓

**If the event is an SAE that needs to be recorded within BICS, complete an initial SAE report**

- either on a hard copy SAE form (copy in site file or download from downloads section of website, or request a copy from the trial office); **or**
- by entering the information directly into the SAE form on the study website, exporting this as a pdf and printing a copy.

↓

**Assessment and signature**

The PI (or consultant level delegate) should record assessment of seriousness, relationship to study medication and expectedness on page 1 of the SAE form, and sign the final page of the SAE form.

If the PI or delegate is not available to make the assessment and/or sign the SAE form, DO NOT delay reporting it to the trial office. It can be forwarded without assessment or signature.

↓

**Report to the trial office**

- Within 24 hours of the local research team becoming aware of a potential SAE, email a copy of the signed SAE form to bics@abdn.ac.uk, or fax a copy of the signed SAE form to 01224 438165.
- If you do not receive an acknowledgement, resend or contact the trial team.

↓

**On receipt, the trial office will:**

- Notify the SAE to the sponsor
- Forward the sponsor assessment of the SAE to you, together with any requests made by sponsor (e.g. for clarification of information provided or the need for a follow-up SAE form)
- Carry out any onward reporting that is required.
- Enter the SAE into the trial database (if the original was completed by hand rather than on the database, and notify the site when this has been done).

## Appendix 4 – Authorship policy for the BICS study

(based on version 3 of the CHaRT authorship policy, dated January 2017)

### 1. DEFINING AUTHORSHIP

Authorship of published or presented papers is based on the following criteria[1]:

i. Substantial contributions to the conception or design of the work; or the acquisition, analysis, or interpretation of data for the work; AND
ii. Drafting the work or revising it critically for important intellectual content; AND
iii. Final approval of the version to be published; AND
iv. Agreement to be accountable for all aspects of the work in ensuring that questions related to the accuracy or integrity of any part of the work are appropriately investigated and resolved.

### 2. PRINCIPLES OF AUTHORSHIP

The following principles of authorship have been derived from editorial publications from leading journals[2,3] and are in accordance with the rules of the International Committee of Medical Journal Editors (ICMJE)[1].

All contributors must fulfil the criteria detailed in section 1: DEFINING AUTHORSHIP in order to qualify for authorship.

Contributors who meet fewer than all four of the criteria for authorship listed above should not be listed as authors, but they should be acknowledged. For example, participation solely in the acquisition of funding, collection of data or technical editing, language editing or proofreading the article is insufficient by itself to justify authorship[1]. Those persons may be acknowledged and their contribution described. See section 3: ACKNOWLEDGEMENTS.

#### a. Preferred CHaRT authorship

Where possible, all CHaRT studies should publish using all the named contributors who qualify for authorship in the byline i.e. Jane Doe, John Doe, John Smith and Ann Other.

However, there may be situations where this is not possible, for example if the journal limits the number of authors. In such circumstance, group authorship may be appropriate using bylines similar to “The BICS trial group” or “Jane Doe, John Doe, John Smith, Ann Other and the BICS trial group”. The article should carry a footnote of the names of the people (and their institutions) represented by the corporate title.

Group authorship may also be appropriate for publications where one or more authors take responsibility for a group, in which case the other group members are not authors but may be listed in the acknowledgement (the byline would read 'Jane Doe *for* the Trial Group')[2]. Again, the article should carry a footnote of the names of the people (and their institutions) represented by the corporate title.

#### b. Determining authorship

These authorship criteria are intended to reserve the status of authorship for those who deserve credit and can take responsibility for the work. The criteria are not intended for use as a means to disqualify colleagues from authorship who otherwise meet authorship criteria by denying them the opportunity to meet criterion

numbers (ii) or (iii). Therefore, all individuals who meet the first criterion should have the opportunity to participate in the review, drafting, and final approval of the manuscript[1].

Tentative decisions on authorship should be made as early as possible[3]. These should be justified to, and agreed by, the Project Management Group. Any difficulties or disagreements will be resolved by the Trial Steering Committee (TSC).

**c. Ordering of authors**

The following rules may help with the ordering of authors, particularly for publications with individual authorship:

i. The person who has taken the lead in writing may be the first author.
ii. The senior author may wish to be the last named author.
iii. Those who have made a major contribution to analysis or writing (i.e. have done more than commenting in detail on successive drafts) may follow the first author immediately; where there is a clear difference in the size of these contributions, this should be reflected in the order of these authors.
iv. All others who fulfil the four authorship criteria described in Section 1: DEFINING AUTHORSHIP may complete the list in alphabetical order of their surnames.

## 3. ACKNOWLEDGEMENTS

All those who make a contribution to a publication, but who do not fulfil the criteria for authorship, such as interviewers, data processors, staff at the recruiting sites, secretaries and funding bodies, should be acknowledged by name, usually in an 'Acknowledgements' section specifying their contributions. Because acknowledgment may imply endorsement by acknowledged individuals of a study's data and conclusions, authors are advised to obtain written permission to be acknowledged from all acknowledged individuals[1].

## 4. DISCLAIMERS

All papers arising from CHaRT must include the full title of the Health Services Research Unit (HSRU) and the appropriate disclaimer specified by the Chief Scientist Office (CSO). For the current disclaimer please see Q-Pulse.

Authors should also ensure they include the study funder's disclaimer: refer to the funders website for details. Be aware that other disclaimers may also be required.

## 5. QUALITY ASSURANCE

Ensuring quality assurance is essential to the good name of the trial group. All reports of work arising from the BICS trial, including conference abstracts, should be peer reviewed by the Project Management Group. The Project Management Group will be responsible for decisions about submission following internal peer review. Submission may be delayed or vetoed if there are serious concerns about the scientific quality of the report. If individual members of the group are dissatisfied by decisions, the matter may be referred to the TSC.

It is hoped that the adoption and dissemination of this policy will prevent disputes that cannot be resolved by informal discussion. However, any member off the study team with a concern about authorship should discuss it with the relevant Chief Investigator, TSC, Line Manager or Programme Director as appropriate.

## REFERENCES

1. Recommendations for the Conduct, Reporting, Editing, and Publication of Scholarly Work in Medical Journals. Developed by members of the ICMJE over the period 2011 to 2013. (www.icmje.org/#authors)
2. Huth EJ (1986). Guidelines on authorship of medical papers. *Annals of Internal Medicine*, **104**, 269-274.
3. Glass RM (1992). New information for authors and readers. Group authorship, acknowledgements and rejected manuscripts. *Journal of the American Medical Association*, **268**, 99.

## Appendix 5 – Amendment History

| Amendment number | Protocol version number. | Date | Author(s) of changes | Details of changes made |
|---|---|---|---|---|
| | 1 (new document) | 12 February 2018 | | |
| n/a – changes required by REC | 2 | 30 April 2018 | Seonaidh Cotton | **Changes required by REC:**<br>• Addition of people without capacity to the exclusion criteria |
| n/a – changes required by MHRA | 3 | 25 May 2018 | Seonaidh Cotton | **Changes required by MHRA:**<br>• Addition of pregnancy testing for women of childbearing age<br>• Addition of section on contraceptive requirements<br>• Clarification of process for reporting urgent safety measures |
| Amendment 1 | 4 | 30 May 2018 | Seonaidh Cotton | • Section 6.2 - removal of requirement to have ECG (amended throughout protocol)<br>• Section 7.2 – consent received<br>• Section 7.6 – confirmation (at each visit) that participant is content to continue in the trial<br>• Section 8.4 – clarification of process for drug disposal<br>• Section 9.11 – clarification in relation to incidental findings<br>• Section 11.4 – amendment to the archiving period (25 years)<br>• Appendix 6 – addition of BICS Heart Study protocol |
| Amendments 2-10 | n/a | n/a | n/a | • Addition of sites/changes to PIs. No revision to protocol or other documentation. |
| Amendment 11 | 5 | 23 March 2020 | Seonaidh Cotton | • Appendix 7 – addition of continency arrangements in response to COVID-19 pandemic. |
| Amendment 12 | n/a | 24 June 2020 | n/a | • Changes to PIs at three sites. No revision to protocol or other documentation. |
| Amendment 13 | 6 | 1 December 2020 | Seonaidh Cotton | **COVID-19 related**<br>• Summary of the three main protocol changes to allow recruitment to restart and the justification for this (Appendix 8) |

| | | | | |
|---|---|---|---|---|
| | | | | • Revisions to inclusion criteria (synopsis, scientific summary; trial flow-chart; section 6.1, 7.1.2<br>• Revisions to the titration (synopsis, lay summary, scientific summary, trial flow-chart, section 8.7.1, 8.7.2,<br>• Reduction in number of face-to-face study visits and associated processes (lay summary, scientific summary, section 4, section 7.1.1, section 7.1.3, section 7.2, 7.2.2, section 7.6, section 9.6.1)<br>• Update to secondary outcomes (synopsis, scientific summary, trial flow-chart; 3.2; 3.4<br>• Mitigation of risk (section 2.1)<br><br>**Non-COVID related**<br>• Signature page – addition of clinical trials pharmacist signature<br>• Updates to membership/contact details for TSC<br>• Section 7.2 – addition of text in relation to patients who cannot read and/or write<br>• Section 7.5 – addition of text in relation to unblinding of the DMC in relation to SAEs<br>• Section 8.4 – reference to stability data at temperatures higher than 25°C<br>• Sections 8.6 and 8.7 – relaxation of the timing around dose titration to accommodate the pragmatic nature of the study<br>• Section 9.2 – confirmation that pneumonia should be captured as an SAE<br>• Appendix 6 - relaxation of the timing around the cardiac sub-study to accommodate the pragmatic nature of the study |
| Amendment 14 | 7 | 14 May 2021 | Seonaidh Cotton | • Section 8.11 – confirmation that during pandemics participants should return unused medication to a local pharmacy for destruction.<br>• Section 13.9 – confirmation that digital sphygmomanometers will be provided to sites for participants to |

| | | | | |
|---|---|---|---|---|
| | | | | assess their heart rate and blood pressure at home<br>• Appendix 1 – addition of statement confirming no interaction between COVID vaccines and the study IMP.<br>• Appendix 6 – addition of high sensitivity C-reactive protein (hs-CRP) and Galectin to the blood tests that will be done<br>• Correction of minor typographical errors and inconsistencies within the protocol. |

## APPENDIX 6: Protocol for a cardiac sub-study embedded within BICS

# THE BICS Heart Study - A study within BICS to see if any effects of beta-blockers in COPD are restricted to those at risk of heart disease

| | |
|---|---|
| Key additional Contributors | Dr Dana Dawson<br>Reader in Cardiovascular Medicine and Consultant Cardiologist<br>University of Aberdeen<br>Division of Applied Medicine<br>Polwarth Building<br>Foresterhill<br>Aberdeen<br>AB25 2ZD<br><br>Tel: 01224 437969<br>Email: dana.dawson@abdn.ac.uk<br><br>Prof Chim Lang<br>Professor of Cardiology<br>University of Dundee<br>Ninewells Hospital and Medical School<br>Mailbox 2<br>Dundee<br>DD1 9SY<br><br>Tel: 01382 383013<br>Email: C.C.Lang@dundee.ac.uk<br><br>Professor Nicholas Mills<br>Professor of Cardiology<br>University of Edinburgh<br>SU.226 Chancellor's Building<br>49 Little France Crescent<br>Edinburgh<br>EH16 4SB<br><br>Tel: 0131 242 6517<br>Email: Nick.Mills@ed.ac.uk<br><br>Professor Mark Petrie<br>Professor of Cardiology<br>University of Glasgow<br>Glasgow Cardiovascular Research Centre<br>Byres Road<br>Glasgow<br><br>Tel: 01412114833<br>Email: Mark.Petrie@glasgow.ac.uk |

**ABSTRACT**
The Bisoprolol in COPD Study (BICS) is a pragmatic randomised double-blind placebo-controlled trial investigating the effectiveness of the beta-blocker Bisoprolol in reducing exacerbations in people with Chronic Obstructive Pulmonary Disease (COPD) who do not currently fulfil criteria for beta-blocker use. A cardiac sub-study nested within BICS will test the hypothesis that the beneficial effects of beta-blockers on COPD exacerbations are restricted to COPD patients at high risk of occult, previously undiagnosed heart disease. To do this, in the first 12 weeks during their involvement in BICS, participants recruited in secondary care sites will be invited to participate in the sub-study comprising an echocardiogram at the start of the one year treatment period and blood samples for measurement of biomarkers: N-terminal pro-Brain Natriuretic Peptide (NT-proBNP), high sensitivity Troponin I (hs TnI), high sensitivity C-reactive protein (hs-CRP) and Galectin at the start and end of the one year treatment period. There are no changes to the main BICS protocol. These investigations will be used to further analyse the effects of beta-blockers on COPD exacerbations by risk stratifying participants for heart disease. This sub-study will establish if beta-blocker therapy is beneficial in all COPD patients (via a yet to be established mechanism) or in a sub-group of COPD patients with occult heart disease (via cardiac protection).

**SYNOPSIS**

| | |
|---|---|
| Title | Cardiac sub-study embedded within: A randomised, double-blind placebo controlled trial to test if a beneficial effect of beta-blockers on exacerbations of chronic obstructive pulmonary disease (COPD). |
| Short title & Acronym | BICS Heart Study |
| Rationale | The Bisoprolol in COPD Study (BICS) is investigating the effectiveness of the beta-blocker Bisoprolol in reducing exacerbations in people with Chronic Obstructive Pulmonary Disease (COPD) who do not currently fulfil criteria for beta-blocker use. The BICS sub-study will risk stratify participants in BICS for heart disease to investigate whether any beneficial effect of beta-blockers is restricted to those at high risk of occult, previously undiagnosed heart disease. |
| Objectives | To determine whether beneficial effects of beta-blockers on COPD exacerbations are restricted to people with COPD at high risk of occult, previously undiagnosed heart disease. |
| Setting | As per BICS |
| Sub-study sample size estimate | BICS participants enrolled in secondary care sites will be invited to participate. We envisage about 900 participants. |
| Sub-study eligibility criteria | BICS participants who have consented to be approached for further studies are eligible. |
| Sub-study assessment | Participants will be invited to take part in the BICS sub-study after they have agreed to take part in the main BICS study. This may be during their BICS recruitment visit or during the titration period. Those who agree will be invited to:<br>undergo echocardiography after commencing study medication (within 12 weeks of randomisation).<br>Participants will be invited to provide a blood sample for measurement of NT-proBNP, hs-TnI, hs-CRP and Galectin soon after commencing study medication (within 12 weeks of randomisation) and at the end of the study treatment period.<br><br>Baseline blood sampling and echocardiography should not be performed within 4 weeks of recovery from an exacerbation of COPD. If the participant experiences prolonged period(s) of exacerbation before starting study medication or during titration, the 12 week window can be extended to permit the participant to take part in the sub-study. If the participant has an exacerbation when the blood/echocardiography are due, the 12 week window can be extended to permit the participant to take part in the sub-study. The 12 month bloods will be taken at 12 month visit (if face-to-face) or during the down-titration of the study medication.<br><br>Note: if sites are unable to offer echocardiography, they can undertake the blood component of the cardiac sub-study. This will be explained to potential participants when they are invited to take part in the cardiac sub-study. If echocardiography becomes available at the site before the participants complete their follow-up within BICS, a late echocardiography could offered to participants. |
| Outcome measures | Primary and secondary outcomes as per BICS. |

| | |
|---|---|
| | The sub-study outcome measures will be NT-proBNP, hs-TnI, hs-CRP, Galectin and echocardiography parameters. |
| Statistical methods | Participants will be risk stratified for occult heart disease and analysed as sub-groups within the main BICS analysis. |

## BACKGROUND

As described in the main protocol the Bisoprolol in COPD Study (BICS) is a pragmatic multicentre randomised double-blind placebo-controlled trial investigating the clinical and cost-effectiveness of adding bisoprolol to usual COPD therapies in patients with COPD at high risk of exacerbation.

Whilst BICS will definitively address the question of the clinical effectiveness of beta-blockers in reducing exacerbations in all patients with COPD who do not currently fulfil the criteria for beta-blocker therapy, there remains the important question as to the mechanism by which beta-blockers reduce exacerbations and whether beta-blocker therapy should be targeted at a readily identifiable sub-group of COPD patients.

The aim of the cardiac investigation sub-study is to risk stratify BICS participants for undiagnosed and clinically relevant cardio-vascular pathology in order to test the hypothesis that any benefits of beta-blocker therapy on COPD exacerbations are restricted to those COPD patients at high risk of occult undiagnosed heart disease.

## SUB-STUDY OBJECTIVES

### Primary Objective

To determine if any clinical success of beta-blocker therapy in COPD patients is restricted to those COPD patients at high risk of occult undiagnosed heart disease.

### Secondary Objectives

To determine the best combination of biomarkers±echocardiography-derived parameters that reliably identifies the subgroup of COPD patients who will benefit from beta-blocker therapy for the purposes of COPD treatment.

## SUB-STUDY DESIGN

Participants in BICS will adhere to the main BICS study protocol. Within the sub-study, a limited number of additional investigations will be performed during BICS assessment visits to risk stratify participants for occult undiagnosed heart disease.

Participants enrolled into BICS in sites that have agreed to participate in the sub-study and who have agreed to be contacted about further studies, will be invited to take part in the sub-study after they have agreed to take part in the main BICS study. This may be during their BICS recruitment visit or during the dose titration phase. After providing written informed consent participants will undergo echocardiography at a mutually convenient time, ideally during one of the dose titration assessments, but up to 12 weeks following randomisation is acceptable (and can be delayed if the participant exacerbates during this period). Participants will also be invited to provide two 15ml blood samples, one ideally during the dose titration phase, but up to 12 weeks following randomisation is acceptable (and can be delayed if the participant exacerbates during this period) and one at the final assessment visit at the 12 month visit (if face-to-face) or during the down-titration of the study medication.

Note: if sites are unable to offer echocardiography, they can undertake the blood component of the cardiac sub-study. This will be explained to potential participants when they are invited to take part in the cardiac sub-study. If echocardiography becomes available at the site before the participants complete their follow-up within BICS, a late echocardiography could be offered to participants.

## STUDY POPULATION

### Number of participants

Participants enrolled into BICS in sub-study sites will be invited to take part, the aim is to recruit about 900 participants into the cardiac sub-study.

**Inclusion criteria**
Participants in enrolled in BICS who have consented to be approached for further studies.

**Exclusion criteria**
There are no exclusion criteria.

**Identification of participants**
Participants enrolled into BICS in secondary care sites will be invited to take part during the dose titration phase. They will be invited by a member of the BICS local study team. If they are potentially interested in takin part, they will be given the patient information leaflet for the BICS Heart Study.

**Consenting participants**
Written and verbal explanation of the study aims and protocol will be provided detailing the nature of the study, the procedures involved, the known possible side effects and any risks involved in taking part. It will be clearly stated that the individual is free to withdraw from the study at any time for any reason without any prejudice to future care or their participation in the main BICS study, and with no obligation to give the reason for withdrawal.

**STUDY ASSESSMENTS**
Participants will adhere to the main BICS assessment regimen. After providing written informed consent for the Heart Study, participants will undergo echocardiography and provide two blood samples at mutually convenient times.

**Blood assays:**
15 ml blood samples will be taken by a member of the BICS research team at the site:
1) Within 12 weeks of randomisation, however, this may be delayed if the participant exacerbates after joining the study
2) At the final study assessment (approximately week 52) (if taking study treatment), however this may be delayed by exacerbation.

The 15ml sample will be processed into a serum (plain) tube (10ml) and an EDTA tube (5ml).

The tubes will be centrifuged at 1000-2000g for 10 minutes, and the plasma and serum stored in ~1ml aliquots) at -70$^{o}$C ideally within 1 hour of collection. Samples will be stored locally and sent in batches to Aberdeen. All samples will be stored at -70$^{o}$C. Samples will be identified by study number and not by the participant's name.

One aliquot per participant per time point will be analysed for N-terminal pro-Brain Natriuretic Peptide (NT-proBNP and Galectin, analysis by Prof Lang in Dundee).

One aliquot per participant per time point will be analysed for high sensitive Troponin I and hs-CRP (hs TnI, analysis by Prof Mills in Edinburgh).

All samples will be analysed in the two core labs mentioned above. Any residual material will be retained in Aberdeen (HRA approved tissue bank - NHS Grampian Biorepository).

**Echocardiography:**
Echocardiography. The echocardiography will be arranged at a convenient time for the participant and the imaging centre – it will either be arranged by a member of the cardiology research team or a member of the BICS research team. Echocardiography would be ideally timed with the initial blood investigations, within 12 weeks from randomisation, however echocardiography may be delayed by exacerbations if the participant exacerbates after joining the study. Echocardiography will be performed according to standard protocols on GE or Philips systems, inclusive of diastolic function, speckle and tissue Doppler imaging for off line analysis. Apical 2,3 and 4 CV will be acquired for accurate computation of EF and atrial volumes. Left ventricular wall thickness derived from Echocardiography will only be used in the detection algorithm for heart failure with preserved ejection fraction[1]. In addition, an estimated Pulmonary artery pressure, tissue Doppler imaging of the mitral and tricuspid annuli and longitudinal, circumferential and radial strain analysis will be derived. Echocardiographs will be identified by study number and, to facilitate any clinical reporting, the participant's name. Echocardiographs will be sent to Aberdeen for analysis by a member of the cardiology research team. Analysis of echocardiographs will only be undertaken after the participant has completed 12 month follow-up (ie after

participant involvement in the trial has finished). Any significant or clinically relevant findings that are likely to significantly impact on the patient's health or future prognosis will be provided to the participant's GP (with the participant's agreement).

Timing of blood samples and echocardiography in patients who are exacerbating
Baseline blood sampling and echocardiography should not be performed within 4 weeks of recovery from an exacerbation of COPD. If the participant experiences prolonged period(s) of exacerbation during titration, the 12 week window can be extended to permit the participant to take part in the sub-study. If the participant has an exacerbation when the blood/echocardiography are due, the 12 week window can be extended to permit the participant to take part in the sub-study.

**RISKS**
Some participants may experience some discomfort from having the blood samples collected and there may be some bruising that will resolve usually within a few days. The risks to the participant will be minimised by having this procedure performed by suitably trained members of staff. We are not aware of any risks of echocardiography.

The echocardiography and blood samples (soon after recruitment) and blood samples (around the 12 month follow-up) will require an additional two face-to-face visits for participants. Appropriate COVID-19 precautions will be taken (PPE, social distancing, hand hygiene).

**ETHICAL CONSIDERATIONS**
In the BICS participant information sheet potential participants will be made aware that they might be approached for a further study, written informed consent will be obtained for this.

Participants will be approached to take part in the sub-study during the dose titration phase by a member of the BICS local study team and provided with a sub-study participant information sheet. Participants will be invited to take part in the BICS sub-study after they have agreed to take part in the main BICS study. Research nurses will be allowed discretion as to the exact timing of the approach which may be during their BICS recruitment visit or during the titration period. Participants expressing an interest (during ensuing week or at subsequent assessment) will be invited to provide written informed consent. If participants wish to give consent at the time they are provided with information about the sub-study, they can provide written informed consent at that time if they have read the information. The same arrangements for patients who cannot read and/or write as described in section 7.2 will apply to the cardiac sub-study.

Participants will be made aware that their blood samples will be sent to Edinburgh/Dundee for analysis. With consent, surplus samples will be stored.

The echocardiogram and blood tests (NT-proBNP, hs-TNI hs-CRP and Galectin) are not part of the routine care of people with COPD. To preserve the pragmatic nature of the BICS trial and to prevent the introduction of bias, all samples/images will be analysed a minimum of 12 months after randomisation when the participant has ceased involvement in the trial. If an immediately life threatening abnormality is obvious to the echocardiographer this will be discussed on an individual basis with the study team. Participants will be informed of this in the participant information sheet. Relevant results from the echocardiography will be issued as clinical reports to the participant's GP and/or local cardiology team.

**DATA COLLECTION**
Echocardiographic and blood data collected during the study will be stored in the main BICS study database.

**PARTICIPANT WITHDRAWAL**
Participants who decline further contact within the main BICS study will not be contacted further about the sub-study. Participants who decline to attend follow-up appointments within the main BICS study will not be contacted further about the sub-study.

Participants who cease study medication, or who fix their dose on 0 tablets are eligible for, and may continue in the sub-study. For these participants, there is no change to the timing of the sub-study assessments.

## STATISTICS AND DATA ANALYSIS

### Sample size

The sample size of 1574 for BICS is based on a reported mean (SD) rate of exacerbation for patients with COPD with ≥2 exacerbations in the previous year of 2.22 (1.86) in the subsequent year[2]. BICS will be able to detect a 15% reduction in COPD exacerbations with 90% power at 5% significance level. In a review of the literature, Rutten et al[3] reported that the prevalence of left ventricular systolic dysfunction in people with COPD varies considerably, with the highest prevalence (46%) amongst those with an exacerbation.

Recruitment of 900 BICS participants to the cardiac sub-study will have 80% power at the 5% level of significance to detect the following differences in rate of exacerbation in bisoprolol and placebo arms depending on the proportion of participants with occult heart disease. The power calculations factor in 10% of participants stopping study medication.

| % high risk occult heart disease | Exacerbation rate: placebo arm | Exacerbation rate: bisoprolol arm | Absolute difference exacerbation rate: bisoprolol vs placebo | % difference in exacerbation rate: bisoprolol vs placebo |
|---|---|---|---|---|
| 50% | 2.22 | 1.70 | 0.52 | 23% |
| 40% | 2.22 | 1.64 | 0.58 | 26% |
| 30% | 2.22 | 1.55 | 0.67 | 30% |
| 20% | 2.22 | 1.40 | 0.82 | 37% |

The two possible extreme situations are that the sub-study:
a). Will have 80% power at the 5% level of significance to detect a 23% reduction in the rate of exacerbation (2.22/yr vs 1.70/yr) if 50% of participants have occult heart disease.
b). Will have 80% power at the 5% level of significance to detect a 37% reduction in the rate of exacerbation with bisoprolol (2.22/yr vs 1.40/yr) if only 20% of participants have occult heart disease.

The systematic review of Du et al[4] reported that the observational studies of beta blockers in people with COPD is associated with a 37% reduction in exacerbations (95%CI 29-43%).

## PROPOSED ANALYSES

Predefined cutoffs will be used to risk stratify sub-study participants for occult, previously undiagnosed heart disease:

Hs TnI levels will be analysed using cutoffs that we have shown to be highly predictive in detecting subjects at high risk of silent cardiac target organ damage[5].

Pre-determined imaging cut-offs for in defining “occult, previously undiagnosed heart disease:
1. Echo bi-plane LV EF<40% with dilated or non-dilated LV[1].
2. Heart failure with mid-range EF (40-49%) for as per most recent ESC guidelines[1]
3. Heart failure with preserved EF (≥50%) who additionally have, as per ESC guidelines[1]:
    i. either NT-proBNP≥125 pg/ml
    ii. or with evidence of diastolic dysfunction (defined as abnormal LV relaxation by Doppler imaging, ie E/E’>13 or echo-enhanced left atrial volume>34 ml/m2 or left ventricular hypertrophy diagnosed as LV mass index ≥115 g/m$^2$ for males and ≥95 g/m$^2$ for females. If current guidelines are superseded during the course of the study, the diagnostic criteria described in these will be used.

Statistical analyses will be according to the intention to treat principle. The primary outcome of number of COPD exacerbations requiring antibiotics and/or oral corticosteroids in the 12 month study period will be compared between randomised groups overall and in each subgroup (high and low risk occult heart disease) using negative binominal regression with length of time in study as an offset. To assess which biomarkers, their optimal cut-offs and/or which imaging outcome is the best method of identifying those most likely to benefit we will use ROC analysis along with sensitivity and specificity of the specific biomarker and imaging outcomes in identifying those which will show benefit. Benefit will be defined as no exacerbations during follow-up. We may

use logistic regression to assess which biomarkers/imaging measurements are best predictive alone or in combination of a beneficial outcome in terms of a reduction in COPD exacerbations.

## REFERENCES

1. Ponikowski P, Voors AA, Anker SD, Bueno H, Cleland JG, Coats AJ, et al. ESC Guidelines for the diagnosis and treatment of acute and chronic heart failure: The Task Force for the diagnosis and treatment of acute and chronic heart failure of the European Society of Cardiology (ESC). Developed with the special contribution of the Heart Failure Association (HFA) of the ESC. *Eur. J Heart Fail*.2016;18:891-975.
2. Hurst JR, Vestbo J, Anzueto A, Locantore N, Mullerova H, Tal-Singer R, et al. Susceptibility to exacerbation in chronic obstructive pulmonary disease.N. Engl. J Med.2010;363:1128-1138.
3. Rutten FH, Cramer MJ, Grobbee DE, Sachs AP, Kirkels JH, Lammers JW, Hoes AW. Unrecognized heart failure in elderly patients with stable chronic obstructive pulmonary disease.Eur. Heart J.2005;26:1887-1894.
4. Du Q, Sun Y, Ding N, Lu L, Chen Y. Beta-blockers reduced the risk of mortality and exacerbation in patients with COPD: a meta-analysis of observational studies.*PLoS. One.*2014;9:e113048
5. Nadir MA, Rekhraj S, Wei L, Lim TK, Davidson J, MacDonald TM, et al. Improving the primary prevention of cardiovascular events by using biomarkers to identify individuals with silent heart disease.J Am Coll Cardiol.2012;60:960-968.

## APPENDIX 7: Contingency arrangements for COVID-19 (March 2020)

These arrangements are put in place to minimise the risk to participants during the COVID-19 pandemic. These contingency arrangements will remain in force until advised by the Sponsor, at which point we will revert to the main study protocol.

Face-to-face contact with study participants for study purposes should cease (as described below). This change does not increase the risks for participants within BICS. There are currently no other changes to other processes within the study.

**Study participants who are in titration**

Assessment of symptoms and participant preference should be made using a telephone assessment with a decision to fix on the current tolerated dose or the previously tolerated dose. There should be no up-titration in these situations unless the participant has previously tolerated a higher dose (and has down titrated due to an exacerbation of their COPD).

If a participant has a home blood pressure monitoring system, data from this can also be collected during the telephone assessment (along with symptoms and patient preference) and used in decision making. If information on heart rate and blood pressure is available and heart rate is 60 bpm or more and systolic BP 100mmHg or more, up-titration can be carried out. If this is done, the participant must be followed up with further telephone contact approximately one week later to confirm tolerance of the dose.

Guidance on the titration of individual participants can be sought from the CI via the trial office team.

During this period, study participants may not titrate to their maximum tolerated dose. They will not be asked to up-titrate at a later date. We will keep records of the participants affected and consider whether additional statistical analysis should be undertaken, for example a sensitivity analysis excluding the affected participants – if so, this will be reflected in the Statistical Analysis Plan.

**Six and twelve month follow-up**

Follow-up will be by telephone rather than face-to-face.

## APPENDIX 8: Contingency arrangements for COVID-19 (restart of recruitment)

We are proposing three main changes to the protocol to allow recruitment to restart.

**1. Reducing the number of face-to-face visits**
We are reducing the number of face-to-face visits to one (recruitment), with other visits carried out by telephone or video call. If sites wish, the recruitment visit can also be done by telephone or video call. Video calls should be done using technologies approved for use in the local area by the NHS. [amendments to section 4, 7.2, 7.2.2, 7.6, 9.6.1.

PIC activity (with face-to-face recruitment) will only be used within the study at times when there are no local travel restrictions in place. At other times, PIC activity will only be used where recruitment and consent is done without a face-to-face visit (see section 7.2 (B)). [amendment to section 7.1]

**2. Amending the inclusion criteria**
We are amending the inclusion criteria. Firstly, we are amending the exacerbation criteria to: A history of at least two exacerbations requiring treatment with antibiotics and/or oral corticosteroid use in the previous year, based on patient report **OR A history of at least two exacerbations within 12 months of each other requiring treatment with antibiotics and/or oral corticosteroid since March 2019**.

Secondly, we are removing the requirement for spirometry at recruitment. The inclusion criteria will be based on an established predominant diagnosis of COPD, based on historic $FEV_1/FVC<0.7$ and $FEV_1<80\%$ predicted. [amendments to section 6.1, 7.1.2]

*Justification for this change:*
*There is anecdotal evidence that shielding has reduced the number of exacerbations experienced by people with COPD. The ECLIPSE study has demonstrated that the frequent exacerbator phenotype is stable for at least 3 years. The modification of the inclusion criteria to include ≥2 exacerbations in any 12 month period since March 2019 enables the identification of the people at high risk of exacerbation during the time of reduced exacerbation risk during lockdown by combining exacerbations before and after the lockdown.*

*Spirometry is an aerosol generating procedure and therefore is currently not being done within the NHS unless in exceptional circumstances, with the highest levels of PPE. The use of current or historical evidence of* FEV1/FVC<0.7 *has previously been permitted within the inclusion criteria within the study protocol. Previously, FEV1<80% predicted was required to be demonstrated at baseline. Going forward during the COVID-19 pandemic, historical evidence will be used for both $FEV_1<80\%$ predicted and $FEV_1/FVC<0.7$.*

**3. Amending the titration schedule**
We are amending the titration schedule to remove the criteria based on $FEV_1$. Instead, participants will be asked about changes in breathlessness since recruitment to the study – and dose will be reduced if breathlessness has worsened [*section Figure 1, 8.7.1, 8.7.2*].

*Justification for this change:*
*The original BICS protocol requires the dose of study medication be increased over a period of 4-7 weeks to a maximum of 5mg bisoprolol (placebo equivalent 4 tablets). Decisions to increase, fix or reduce the dose of study drug at each dose titration visit are determined by the presence of intolerable side effects (e.g. fatigue), pulse rate, systolic blood pressure and lung function ($FEV_1$). To aid decision making an online algorithm has been developed that uses these data to recommend dosing decisions.*

*It will be possible to determine the presence of intolerable symptoms during a phone/video dose titration 'visit' and the provision of a digital sphygmomanometer to each participant will enable the measurement of pulse rate and systolic blood pressure. We have investigated the feasibility of participants performing spirometry during the phone/video call dose titration 'visits' but have concluded that this is not a viable option, principally because participant technique is likely to be suboptimal and the need to calibrate the equipment before use (spirometer calibration syringes are prohibitively expensive - £300 each).*

*Digital sphygmomanometers can be purchased for between £30 and £40, and we will use savings on travel expenses to purchase these.*

*As outlined previously in the protocol, the use of cardio-selective betablockers such as bisoprolol in people with COPD is safe. A systematic review of RCTs studying effects of cardio-selective blockers on lung function and respiratory symptoms in people with COPD demonstrated that the acute and chronic use of cardio-selective betablockers does not significantly reduce lung function, increase respiratory symptoms or reduce response to inhaled beta*$_2$ *agonists* [5]*. Moreover, since BICS started recruitment, a placebo controlled randomised clinical trial (BLOCK COPD) has reported that a year's treatment with the cardio-selective betablocker metoprolol (n=268) had no effect on lung function (*$FEV_1$*) when compared with placebo (n=264)*[4]*. We have reviewed the dose titration data for BICS (maintaining the study blind): of the 429 participants recruited before recruitment was suspended, 49 had their dose of study medication reduced during dose titration because of a reduction in lung function (*$FEV_1$*), with no significant difference between the two randomised study groups (9% vs 13%). In addition, for those who had the dose of study drug reduced during dose titration because of a reduction in* $FEV_1$*, there was no difference in the absolute or relative reduction in* $FEV_1$ *between the two randomised groups and only two of the 49 participants complained of intolerable breathlessness. It would appear that reductions in* $FEV_1$ *during dose titration in BICS appear to be similar in magnitude and frequency between bisoprolol and placebo groups and do not manifest as increased symptoms.*

*It is therefore proposed, during phone/video dose titration, that the measurement of lung function be replaced by asking the participant whether their breathing has deteriorated since starting/ increasing study medication and that the online dosing algorithm be changed to include the answer to this question rather than changes in* $FEV_1$*. If the study team have concerns about potential side effects in a participant during titration (or later on in follow-up), the participant can be seen at a face-to-face visit with appropriate COVID-19 measures in place. We note that national heart failure guidelines do not recommend spirometry during the dose titration of betablockers in people with heart failure (many of whom have COPD)* [3]*. The current dose titration algorithm for BICS is not routine clinical practice for people with heart failure and COPD and the measurement of lung function was included in the current protocol with the deliberate intent of being conservative. The proposed use of the symptom of increased breathlessness is more in keeping with routine clinical practice and will improve the generalisability of the study findings.*

In addition, we are proposing the following:
**4. Participants recruited at sites offering the cardiac sub-study** and who agree to take part in the cardiac sub-study will be asked to participate in an additional two face-to-face visits. The echocardiography and blood samples (soon after recruitment) and blood samples (around the 12 month follow-up) will require an additional two face-to-face visits for participants. Appropriate COVID-19 precautions will be taken (PPE, social distancing, hand hygiene).

BICS

Bisoprolol in COPD Study

# Patient information leaflet

## A study of the beta-blocker bisoprolol in Chronic Obstructive Pulmonary Disease (COPD).

## Bisoprolol in COPD study (BICS).

We would like to invite you to take part in a research study into the treatment of COPD. In this study we are trying to find out if flare-ups of COPD can be prevented by using a tablet called bisoprolol. Bisoprolol is a beta-blocker that has been widely used for a long time to treat heart problems, but it now seems that bisoprolol might also reduce flare ups of COPD regardless of heart problems.

We believe that you might be eligible to take part in this study because you may have had two or more flare ups of your COPD in the last few years.

- Before you decide whether you would like to take part, it is important for you to understand why the research is being done and what it will involve.
- Please take time to read the following information carefully and discuss it with others if you wish.
- Ask us if there is anything that is not clear or if you would like more information.
- Take time to decide whether you wish to take part. If you are interested in taking part, you can make contact with us (our contact details are at the end of this leaflet).
- Thank you for reading this.

**What is the purpose of the study?**
COPD is a common lung condition and one of the features of COPD is that it can suddenly get worse. These ‘flare ups’ are known as exacerbations and are usually treated with antibiotics and steroids. Often flare ups result in people being admitted to hospital.

One of the aims of our research into COPD is to prevent flare ups occurring. Our study is trying to find out if the beta-blocker bisoprolol reduces the number of COPD flare ups. Beta-blockers have been used to treat heart conditions for over 30 years. There is now evidence that people taking beta-blockers who happen to have COPD are less likely to have flare-ups. In our study, we want to investigate whether people with COPD who are started on bisoprolol, are less likely to have flare ups.

In the past, doctors have been reluctant to use beta-blockers in people with COPD because of worries about narrowing the lung airways. The newer more targeted beta blockers such as bisoprolol are now known to be safe in COPD and have been widely used to treat heart conditions in people with COPD.

**Why have I been chosen?**
We are approaching you because we understand that you have COPD. We are approaching people with COPD who:
- Attend certain General Practices.
- Have been admitted to hospital with COPD.
- Attend chest clinics.
- Have been cared for in the community.
- Have attended pulmonary rehabilitation classes.

We are aiming to recruit 1574 people with COPD who have had two or more flare ups in the last year or so. In order to get 1574 people with COPD, the study is taking place in many hospitals and GP practices across the UK.

**Do I have to take part?**
No. It is up to you to decide whether to take part. If you decide to take part, you are still free to withdraw at any time and without giving a reason. A decision to withdraw at any time, or a decision not to take part, will not affect the NHS care you normally receive.

**What will happen to me if I take part?**
To find out if bisoprolol reduces the number of flare ups we are comparing the effects of bisoprolol against the effects of a placebo 'dummy treatment', which looks like the genuine medicine but contains no active ingredient.

To try to make sure the bisoprolol and placebo groups are the same to start with, each person is put into a group selected by chance (randomly by a computer). Half of the 1574 people will take bisoprolol and half will take the placebo for a year (as well as your usual medicines). To make sure that the true effects of bisoprolol are being studied neither you nor your doctor will know which treatment group you are in (although, if your doctor needs to find out he/she can do so).

To work out the best dose of bisoprolol (and placebo) for you we will slowly build up the dose over the first few weeks whilst checking each week how you feel, how your breathing is, and what your pulse and blood pressure is. You can read more about this below. This slow increase in dose is routine for beta blockers such as bisoprolol and ensures safety.

If you are interested in taking part, we will make an appointment to discuss the study with you at a time convenient to you.

<u>The first appointment will take up to an hour.</u> It can be done as a face-to-face appointment at your local GP practice or local hospital, or it can be done by telephone or video call.

<u>During the appointment</u> we will ask you:

- To sign a consent form saying that you are willing to take part in the study.
- To answer some questions about how COPD is affecting your life.
- To let us know what medicines and inhalers you are taking.
- If you come to a face-to-face appointment, we will check your pulse (at wrist) & blood pressure (inflatable cuff around arm). We will also give you a machine that measures pulse and blood pressure and show you how to use this. You can then take this home with you so that you can measure your pulse and blood pressure at home before your next appointment.
- If your first appointment is by telephone, we will send you a pulse and blood pressure monitor before the appointment so that you can measure your pulse and blood pressure during the appointment. This will come with instructions and we can tell you how to use it.

*We will ask you to continue taking all of your usual medicines and tablets* and if you have a flare up/ exacerbation of your COPD needing treatment that you make a note of the dates and treatment. You will be able to write these details down in a ‘flare up/exacerbation’ diary.

*We will ask you to take one study tablet once a day until we speak to you again about one week later*. There is a 50:50 chance that you will be taking bisoprolol or placebo 'dummy treatment.' It is important to realise that no immediate improvement in daily symptoms is likely to occur. Any improvement is likely to be in a reduction in the number of exacerbations.

After this appointment, we will arrange for about 2 months supply of study tablets to be delivered to your house by courier.

We will give you a card to carry that explains that you are taking part in a study. You can show this card to any doctor or nurse that is treating you.

<u>At the second appointment 1-2 weeks later</u>, we will ask you:

- How you are feeling.
- If your breathing has changed.
- To check your pulse and blood pressure using the monitor we gave you.

*Depending how you are feeling and on your breathing, pulse and blood pressure, we may ask you to take two study tablets once a day until we speak to you again about a week later.*

The second, third, fourth and fifth appointments will take less than 30 minutes. It may not be necessary to have all these appointments.

At the third appointment 1-2 weeks later, we will ask you:

- How you are feeling.
- If your breathing has changed.
- To check your pulse and blood pressure using the monitor we gave you.

*Depending how you are feeling, and, on your breathing, pulse and blood pressure, we may ask you to take three study tablets once a day until we speak to you again about a week later*.

At the fourth appointment 1-2 weeks later, we will ask you:

- How you are feeling.
- If your breathing has changed.
- To check your pulse and blood pressure using the monitor we gave you.

*Depending how you are feeling, and, on your breathing, pulse and blood pressure, we may ask you to take four study tablets once a day until we speak to you again about a week later.*

At the fifth appointment 1-2 weeks later, we will ask you:

- How you are feeling.
- If your breathing has changed
- To check your pulse and blood pressure using the monitor we gave you.

*Depending how you are feeling and, on your breathing, pulse and blood pressure, we will either ask you to keep taking four study tablets once a day for a year or to reduce the dose of study tablets for a year*.

*If, during the slow increase in dose over the second to fifth appointments, you start to have side effects then we will reduce the daily dose of tablets and you will remain on the reduced dose for a year.*

*If during the slow increase in dose you feel well but there are changes in breathing, pulse or blood pressure we will not increase the dose further and you will stay on this dose for a year. You can contact the study team at any time if you have questions about your dose of study tablets.*

After 2 months and again after 5 months of treatment you will receive further supplies of study tablets delivered to your house by courier.

After 6 months of treatment we will arrange a telephone or video call at a time convenient to you and ask you:

- how you are getting on with the tablets.
- if you have had any flare ups of your COPD.
- how COPD is affecting your life.
- what medicines and inhalers you are taking.
- To check your pulse and blood pressure using the monitor we gave you.
  This appointment will take about an hour.

After 12 months of treatment we will arrange a telephone or video call at a time convenient to you and ask you:

- how you are getting on with the tablets.
- if you have had any flare ups of your COPD.
- how COPD is affecting your life.
- what medicines and inhalers you are taking.
- To check your pulse and blood pressure using the monitor we gave you.
  This appointment will take about an hour.

We will also tell you how to slowly reduce the dose of study tablets over the next 3 weeks by one tablet a week until you are taking no study tablets at all. Your research nurse will make contact to make sure you have successfully stopped the study tablets.

At the end of the study, we will ask you to take any unused study tablets to your local pharmacy and asking them if they can dispose of them safely.

**Expenses and payments**

If your first appointment is face-to-face, you can receive travel expenses to attend your local study centre. Please ask your research nurse for information about how to claim travel expenses.

**What will I have to do?**

We ask that you:

- Take the study tablet(s) once a day as directed, for a year.
- Participate in up to seven study appointments (first appointment, and then at about 1 week, 2 weeks, 3 weeks, 4 weeks, 6 months, and at 12 months) – most, if not all the appointments will be by telephone or video call.
- Make a record of any flare ups of your COPD whilst on the study tablets.
- Carry a card explaining you are in the study.
- Do not stop the study drug suddenly. People who stop the study drug suddenly can experience a reaction to this. If you do wish to stop, please contact your research nurse who can advise you how to stop more slowly.

Otherwise you should continue on your normal medicines and inhalers and carry on as normal. If you need to go to

your GP for treatment you should go, any flare ups/exacerbations of your COPD can be treated as they normally would.

**What is the drug that is being tested?**
The drug being tested is bisoprolol. Bisoprolol is licensed for people with COPD and heart disease but not for people who have COPD without heart disease. The maximum dose of bisoprolol in this study (5mg a day) is less than the dose usually used to treat heart conditions (10mg a day).

There are some drugs used mainly to treat heart conditions that are known to interact with bisoprolol.

Although we are obliged to tell you about this, you do not need to remember them because:

- We would check whether you are already taking any of these drugs.
- We would let your GP know that you are in the study, which drugs to avoid and what to do if (s)he wants to put you on one of these drugs.
- We would give you a small card to show to nurses or doctors, this will direct them to a list of the drugs to avoid and tell them what to do if they want to put you on one of these drugs.
- If your doctor wants to start you on one of these drugs, then they will tell you to stop taking the study tablets whilst on these drugs. This may mean that you have to stop taking the study tablets permanently.

For reference, the list of drugs to avoid is:

- *Blood pressure/angina tablets called calcium antagonists*; verapamil and diltiazem.
- *Angina tablet*: Ivabradine.

- *Heart rhythm drugs*: quinidine, disopyramide; phenytoin; flecainide, propafenone.
- *Certain blood pressure tablets*: clonidine, methyldopa, moxonodine, rilmenidine.

**What are the side effects of any treatment received when taking part?**

Although most people tolerate bisoprolol well, some people can develop side effects. *We hope to reduce these by slowly increasing the dose of study tablets at the start of the study to find the best dose for you.* If side effects do develop then they should stop when the dose of study tablets is reduced or stopped.

Side effects of bisoprolol

*Common (affect between 1 in 10 to 1 in 100 people)*

Dizziness, headache, nausea, vomiting, diarrhoea, constipation, cold hands & feet, tiredness.

*Uncommon (affect between 1 in 100 to 1 in 1000 people)*

Breathlessness, muscle weakness & cramps, unsettled sleep, low mood.

*Rare (affect between 1 in 1000 to 1 in 10,000 people)*

Blackouts, congested nose, nightmares, hallucinations, dry eyes, reduced hearing, itchy rash, inflamed liver, erectile dysfunction.

*Very rare (affect less than 1 in 10,000 people)*

conjunctivitis, psoriasis, hair loss.

If you decide to take part in our study, we will give you another information leaflet with your medicine that will tell you more about the study tablets, how to take them, and the

possible side effects.  Please read all the information contained in this leaflet.

If you feel unwell at any point, please seek medical help as you would do usually.  If you are concerned that you may have developed side effects, you should contact your local study centre, or your GP and they will provide help and advice.

**What are the possible disadvantages of taking part?**
Other than the potential side effects described above, we do not think there are other risks associated with taking part in BICS.  Taking part may affect your travel insurance or private health insurance – there is more information about this below.  The study involves extra appointments as described in the section "What will happen to me if I take part?", but most (if not all) of these can be done by telephone or video call.

**What are the possible benefits of taking part?**
We cannot promise the study will help you but we expect the information we get from this study will help improve the treatment of people with COPD, reduce the need for antibiotics and steroids and reduce hospital admissions for COPD.

We will investigate whether bisoprolol reduces the number of flare ups in people with COPD, and if it does, whether it reduces the need for antibiotics and steroids and reduce hospital admissions for COPD.

**Travel and private health insurance**
Taking part in a clinical research study such as BICS may affect any travel insurance or private health insurance cover you have.  If your insurer asks you, you need to tell them that you are taking part in a clinical trial.  You must answer all their questions as fully and accurately as you can.  If you do not, your insurer may refuse to pay any claims and could cancel your policy.  We can provide more information to your insurer.

**What happens when the research study stops?**
When you have stopped the study tablets in the weeks after the 12-month appointment, we will not give you any more study tablets.  We would not advise taking bisoprolol for your COPD until we know whether bisoprolol makes a difference in COPD.  We will know this in 2023 when the study has finished.  If, at the end of your 12 months in the study, you feel that the study tablets have improved your COPD we can let your GP know and advise that the dose of bisoprolol would need to be slowly increased. You would need to discuss this with your GP though.

**Involvement of the General Practitioner (GP)**
We will ask your permission to let your GP know that you are taking part in the study.  At the end of the study, again with your permission, we may ask your GP if we can look at your GP records to count up the number of times you have visited your GP and the number of flare ups you have had whilst on the year of study treatment.

**What if relevant new information becomes available?**
If we get any important new information about the use of bisoprolol in people with COPD, your local study team will tell you and discuss with you whether you should continue in the study. If you decide not to carry on, arrangements will be made for your usual NHS care to continue. This may be with your GP or may be with the chest clinic at the hospital. If you decide to continue in the study, you may be asked to sign an updated consent form.

**What will happen if I don't want to carry on with the study?**
You are still free to withdraw at any time and without giving a reason and this will not affect the NHS care you normally receive. Study information collected up to that point may still be used.

If you do wish to stop the study tablets, please contact your local study team and they will advise on how to reduce the dose safely to zero. We will ask you to take any unused study tablets to your local pharmacy and asking them if they can dispose of them safely.

If you stop taking the study tablets you can still have the study appointments at 6 and 12 months.

**What if there is a problem?**
If you believe that you have been harmed in any way by taking part in this study, you have the right to pursue a complaint and seek compensation through the research sponsors of the study - the University of Aberdeen and NHS Grampian.  Contact details for both research sponsors are available through the research team.

As a patient of the NHS if you are harmed due to someone's negligence, then you may have grounds for a legal action, but you may have to pay for it. Regardless of this, if you wish to complain, or have any concerns about any aspect of the way you have been approached or treated during the course of this study, the normal National Health Service complaints mechanisms would be available to you.

If you have a concern about any aspect of this study, you should ask to speak to the study doctors who will answer your questions (contact details are at the end of this information leaflet). If you remain unhappy and wish to complain formally, you can do this through the NHS Complaints mechanisms (or Private Institution). Contact details can be obtained from your local hospital. In addition to this, you may contact the chairman of the BICS Trial Steering Committee (who is independent from the study) through the BICS study office.

**Will my taking part in the study be kept confidential?**
Yes. We will follow ethical and legal practice and all information about you will be handled in confidence. All information which is collected about you during the research will be kept strictly confidential and will be held securely for 25 years in accordance with the latest Data Protection legislation.

The University of Aberdeen will be the data controller for this study and is responsible for looking after your information and using it properly. You can find more about this at www.abdn.ac.uk/privacy.

The local research team at the hospital or GP practice that you were recruited in will have access to your information. They will use your name and contact details to contact you about the study, to make sure that relevant information about the study is recorded for your care, and to oversee the quality of the study.

The study is being co-ordinated by the Centre for Healthcare Randomised Trials (CHaRT), a registered clinical trials unit at the University of Aberdeen.  The study team in CHaRT will have access to your information and will keep this confidential.  They will have access to your information to contact you about the study and for quality control purposes.

We will ask you to give us the name and address of someone that you know who we can contact if we cannot get in touch with you.  This might be a family member, friend or neighbour.  Again, with your permission, we will tell this person that you are taking part in the study.

With your permission we will need to pass on your name and address to Clinical Trials Pharmacy who will organise the study tablets for you and the courier company who will deliver the study tablets to your home.  These details will be kept strictly confidential and be used only to send you the tablets.

We will keep the study data for at least 25 years.  It will be kept confidential at all times.

The statistical analysis of the study is being conducted at the University of Aberdeen, and to maintain confidentiality, the statistical team will only analyse completely anonymous data.  (Anonymous data does not include names or addresses, and it is not possible to identify individual participants from anonymous data).  Any reports or publications arising from the study will contain totally anonymous data so that you cannot be recognised from it.

Other researchers may wish to access anonymous data from this study in the future.  If this is the case, they would be expected to follow legal, data protection and ethical guidelines.  It will not be possible to identify you from this data.

If you join the study, the data collected for the study, together with any relevant medical records, may be looked at by authorised persons from the University of Aberdeen, the Research and Development Department of your local NHS Organisation and the Regulatory Authorities to check that the study is being carried out correctly. All will have a duty of confidentiality to you as a research participant.

We will ask you whether you would like to be approached about other research studies.  If you agree to this, we would not share your name or address with the researchers who are running these studies.  Instead, a member of the BICS research team would tell you about the other study and give you details of how to contact the study team if you wanted to take part.  If you do not want to be approached about other research studies, please tell us.

Your rights to access, change or move your information are limited, as we need to manage your information in specific ways in order for the research to be reliable and accurate.

**What will happen to the results of the research study?**

The results of the study will be published in scientific journals and presented at scientific meetings. In addition, with the help of the British Lung Foundation and Chest Heart & Stroke Scotland we will pass on the results to other people with chest disease. It will not be possible to identify you from the results of the study. We will also send you a summary of the results. This will be when all the people taking part in the study have completed their follow-up in the study (this may be later than when you would finish your follow-up in the study) and all the information collected in the study has been analysed. When we send you the results of the study, we can let you know whether you were taking the beta-blocker tablets or the placebo tablets. But we will not tell you this at the end of your follow-up unless you or your doctor need to know this information to plan future treatment.

**Who is organising and funding the research?**

This study is being organised by the University of Aberdeen and chest doctors in Aberdeen, Birmingham, Dundee, Edinburgh, Glasgow, Hull, Liverpool, London, Manchester, Newcastle, and Norwich. The research is being funded by the National Institute for Health Research, Health Technology Assessment programme: NIHR-HTA.

The study is being co-ordinated by the Centre for Healthcare Randomised Trials (CHaRT), a registered clinical trials unit at the University of Aberdeen.

**Who has reviewed the study?**
The study has been reviewed by an NHS Research Ethics Committee, which has responsibility for scrutinising proposals for medical research on humans. In this case, the reviewing committee was Scotland A REC, who have raised no objections to the study.

In addition, the study has also been reviewed and approved by the Medicines and Healthcare Products Regulatory Agency. The Research and Development Department of your local hospital and/or local primary care organisation has also reviewed and approved the study.

**Contact for further information**
If you have any questions or would like more information, please contact us. Our contact details are below.

If you are interested in taking part in this research, please let us know. You can telephone us, email us, or use the reply slip and freepost envelope. Our contact details are below.

<<insert local contact details>>

**Thank you for taking the time to read this information leaflet. We hope that you have found it useful in deciding whether or not to take part in the BICS study.**

**Participant study number**

| | | | | | |
|---|---|---|---|---|---|
| | | | | | |

# Consent form

BICS
Bisoprolol in COPD Study

**Please initial**

**By initialling each box and signing this form, I confirm that:**

1. I have read and understand the information leaflet Version No: …………. Date: ……………….. for the above study. I have had the opportunity to consider the information, ask questions and have had these answered satisfactorily.

2. I understand that my participation is voluntary and that I am free to withdraw at any time, without giving any reason, without my medical care or legal rights being affected. Data collected up until the point of withdrawal may still be used in analysis.

3. I understand that relevant sections of my medical notes and data collected during the study may be looked at by individuals from the University of Aberdeen, from regulatory authorities if appropriate, or from the NHS Board/Trust, where it is relevant to my taking part in this research. I give permission for these individuals to have access to my records.

4. I agree that my GP, my hospital consultant and the person I have nominated as my best contact may be told that I am taking part in this study.

5. I agree that I may be contacted by the study team for future ethically approved studies. I understand identifiable contact information will be kept after the end of this study and this information will be held in accordance with the data protection act and its replacement, the general data protection regulation.

6. I understand that relevant data collected for the purpose of the trial together with personal contact details will be confidentially and securely stored by the University of Aberdeen for no less than 25 years. I agree for my information to be stored on computer servers maintained by the University of Aberdeen. I understand that my contact details will be shared with the central Clinical Trials Pharmacy and the company that will deliver the study medication to my home. I agree that the study co-ordinators can use my contact details to send me study questionnaires and to contact me by telephone or post or email or text.

7. I understand that the information collected about me may be shared anonymously with other researchers to support future research

8. I agree to take part in the above study.

| **Name of participant** | **Date** | **Signature** |
|---|---|---|
| **Name of researcher** | **Date** | **Signature** |

Copies: Original for research site file; copies for participant, medical notes and trial office

BICS study office, Centre for Healthcare Randomised Trials (CHaRT), Health Services Research Unit, University of Aberdeen, Scotland AB25 2ZD; Tel 01224 438178; 01224 438165; bics@abdn.ac.uk.
